# Proceedings of AllerGen 2019 Research Conference

**DOI:** 10.1186/s13223-019-0330-9

**Published:** 2019-04-10

## Poster Competition Abstracts

### A1 Cord blood hemopoietic progenitor cell surface receptor expression in atopic sensitization and lung function

#### Loubna Akhabir^1,7,#^, Elli Rosenberg^1,#^, Delia Heroux^1^, Jyoti Balhara^1^, Kelly M. McNagny^2^, Malcolm R. Sears^1^, Diana L. Lefebvre^1^, Allan B. Becker^3^, Stuart E. Turvey^4^, Piush J. Mandhane^5^, Theo J. Moraes^6^, Padmaja Subbarao^6^, Guillaume Paré^7,8,9^, Judah A Denburg^1^

##### ^1^Department of Medicine, McMaster University, Hamilton, ON, Canada; ^2^Biomedical Research Centre, The University of British Columbia, Vancouver, BC, Canada; ^3^Department of Pediatrics and Child Health, University of Manitoba, Children’s Hospital Research Institute of Manitoba, Winnipeg, MB, Canada; ^4^Department of Pediatrics, BC Children’s Hospital, University of British Columbia, Vancouver, BC, Canada; ^5^Department of Pediatrics, University of Alberta, Edmonton, AB, Canada; ^6^Department of Pediatrics, Hospital for Sick Children, University of Toronto, Toronto, ON, Canada; ^7^Population Health Research Institute, McMaster University, Hamilton, ON, Canada; ^8^Thrombosis and Atherosclerosis Research Institute, McMaster University, Hamilton Health Sciences, Hamilton, ON, Canada; ^9^Department of Health Research Methods, Evidence, and Impact, McMaster University, Hamilton, ON, Canada

###### **Correspondence:** Judah A. Denburg (denburg@mcmaster.ca)

^#^These authors contributed equally to the work.

*Allergy, Asthma & Clinical Immunology* 2019, **15**(**Suppl 2**):A1

**Background:** Hemopoietic progenitor cells (HPC), both in the bone marrow and in peripheral tissues, differentiate into inflammatory effector cells and, thus, can modulate central and peripheral inflammation. There is growing evidence for the involvement of hemopoietic processes in the pathogenesis of atopy and asthma from pre-conception and birth. This is the basis for the “bone marrow” hypothesis of allergic disease, arguing that a perinatal environmental challenge leads to the skewed production and mobilization of HPC, regulating central and peripheral production of cell types that perpetuate allergic responses.The objective of this study was to assess the association of cell surface receptor profiles of cord blood (CB) HPC with atopy and allergic disease development and lung function at 1- and 3-years in the Canadian Healthy Infant Longitudinal Development (CHILD) Study.

**Methods:** We used flow cytometry to compare cytokine and toll-like receptor expression levels in CB HPC from infants who developed atopic sensitization (as assessed by positive skin prick test) at 1 and 3 years of age with healthy controls. We also compared the CB HPC receptor expression in relation to lung function as measured by lung clearance index (LCI) in the CHILD Study infants.

**Results:** We found a significant increase in IL5RA-expressing HPC populations in the CB of cases at 1 and a trend towards increased IL17RB-expressing HPC in the CB of atopics at 1-year of age. Conversely, GM-CSFR- and ST2-expressing CB HPC were decreased in atopics both at 1- and 3-years. Additionally, there was evidence of infants with poor lung function at 3-years exhibiting higher IL5RA and IL17RB expression on the surface of CB HPC.

**Conclusions:** This study provides evidence of pre-existing cellular alterations in infants’ CB progenitors at birth, which herald the development of atopy/allergic disease and, potentially, future asthma. The observed pattern of receptor expression suggests Th2 skewing of CB HPC before the onset of allergic disease or measurable lung function deficits. Our results suggest that measurable immune cellular patterns at birth could be utilised to develop novel strategies for atopic/allergic disease interception in infants before disease onset.

**Acknowledgements:** This study was supported by grants from The Allergy, Genes & Environment (AllerGen) Network of Centres of Excellence, and the Canadian Institutes of Health Research.

### A2 Metabolomic profiling of asthmatic children: a promising approach for improving asthma control

#### Mays Al-Dulaymi^1^, Chun Che^1^, Joan Dietz^1^, Anas El-Aneed^2^, Darryl Adamko^1^

##### ^1^Department of Pediatrics, College of Medicine, University of Saskatchewan, Saskatoon, SK, Canada; ^2^College of Pharmacy and Nutrition, University of Saskatchewan, Saskatoon, SK, Canada

###### **Correspondence:** Darryl Adamko (darryl.adamko@usask.ca)

*Allergy, Asthma & Clinical Immunology* 2019, **15**(**Suppl 2**):A2

**Background:** Pediatric asthma management can be a challenge in a typical primary care setting where we often lack objective tests for asthma diagnosis and severity. Metabolomics is the study of small molecules created by cellular metabolic activity [1, 2]. We have demonstrated that asthma has a different metabolomic profile compared to healthy children [3]. We have identified 50 urinary metabolites as potential diagnostic biomarkers for asthma [4]. Recently, we developed targeted mass spectrometry (MS)-based methods to accurately quantify these biomarkers in urine [5]. For this project, we hypothesize that our novel MS-based method will differentiate healthy children from those with asthma. We also expect that we will see changes in the urine of children with asthma depending on whether they have well controlled asthma versus uncontrolled asthma.

**Methods:** To determine asthma severity, we recruited children with atopic asthma (n = 18) and followed them monthly (July–November). An Asthma Control Questionnaire, Mini Pediatric Asthma Quality of Life Questionnaire and Asthma Control Test were filled at each visit. Pulmonary function tests were performed on all children 6 years of age or older. Urine samples were collected from all children during their appointments. The urine samples were analyzed using our targeted liquid chromatography tandem mass spectrometric platform and value were normalized to creatinine. Partial least squares discriminant analysis (PLS-DA, SIMCA) was used on these data to create models of separation. To diagnose asthma in young children, we also have obtained urine samples from healthy children or those with asthma (n = 100 each) diagnosed at 5 years of age from the CHILD birth cohort. Similar methods are being applied and the data is expected for the January meeting.

**Results:** There were 21 instances where patients’ asthma control worsened and 30 instances where their asthma control improved. Preliminary PLS-DA modelling generated a separation model of controlled and uncontrolled asthma with an R2 value of 0.72 and a Q2 value of 0.631.

**Conclusion:** Positive results were attained in the differentiation of asthma severity. Blinded analysis will be done using a separate cohort of children. Urine samples from the CHILD study are being analyzed and will ready for presentation at the January AllerGen meeting. We expect that further assessment of the urinary biomarkers will create a metabolomic signature that could diagnose asthma in pre-school children and predict the development of uncontrolled asthma.

**References**

1. Nobakht M. Gh BF, Aliannejad R, Rezaei-Tavirani M, Taheri S, Oskouie AA. The metabolomics of airway diseases, including COPD, asthma and cystic fibrosis. Biomarkers. 2015;20:5–16.
2. Zhang A, Sun H, Wu X, Wang X. Urine metabolomics. Clin Chim Acta. 2012;414:65–9.
3. Skappak CD, Regush S, Cook K, Ben-Zvi A, Becker A, Moqbel R, et al. Metabolomic profiling of asthma: diagnostic utility of urine nuclear magnetic resonance spectroscopy. J Allergy Clin Immunol. 2011;127:757–64.e6.
4. Adamko DJ, Nair P, Mayers I, Tsuyuki RT, Regush S, Rowe BH. Metabolomic profiling of asthma and chronic obstructive pulmonary disease: a pilot study differentiating diseases. J Allergy Clin Immunol. 2015;136:571–80.e3.
5. Khamis MM, Adamko DJ, El-Aneed A. Development of a validated LC–MS/MS method for the quantification of 19 endogenous asthma/COPD potential urinary biomarkers. Anal Chim Acta. 2017;989:45–58.

### A3 Genomic variants correlated with cognitive behavioral development in the Canadian Healthy Infant Longitudinal Development (CHILD) Study

#### Amirthagowri Ambalavanan^1^, Jihoon Choi^1^, Amel Lamri^2^, Sukhpreet K. Tamana^3^, Sonia S. Anand^3^, Diana L. Lefebvre^2^, Malcolm R. Sears^2^, Meghan B. Azad^4^, Allan B. Becker^4^, Stuart E. Turvey^5^, Theo J. Moraes^6,7^, Padmaja Subbarao^6,7^, Piush J. Mandhane^3^, Qingling Duan^1,8^

##### ^1^Department of Biomedical and Molecular Sciences, Queens University, Kingston, Ontario, Canada; ^2^Dept of Medicine, McMaster University, Hamilton, Ontario, Canada; ^3^Department of Pediatrics, University of Alberta, Edmonton, Alberta, Canada; ^4^Department of Pediatrics & Child Health and Community Health Sciences, University of Manitoba, Winnipeg, MB, Canada; ^5^Department of Pediatrics, University of British Columbia, Vancouver, British Columbia, Canada; ^6^Department of Paediatrics, University of Toronto, Toronto, Ontario, Canada; ^7^The Hospital for Sick Children, Toronto, Ontario, Canada; ^8^School of Computing, Queen’s University, Kingston, Ontario, Canada

###### **Correspondence:** Qingling Duan (qingling.duan@queensu.ca)

*Allergy, Asthma & Clinical Immunology* 2019, **15**(**Suppl 2**):A3

**Background:** Sleep disordered breathing (SDB), a collective term for chronic conditions including habitual snoring and obstructive sleep apnea, affects up to 10% of children between 2 and 8 years old. Earlier studies have shown that SDB in children is associated with neurobehavioral functions related to executive functioning, behavior development and attention deficit hyperactivity disorder (ADHD) [1]. We hypothesize that preschool SDB may share a common genetic predisposition with neurobehavioral functions during early childhood.

**Methods:** In this study, genomics data were ascertained from the Canadian Healthy Infant Longitudinal Development (CHILD) Study using the Illumina HumanCore Exome BeadChip. A total of 2048 Caucasian subjects had available SDB variables derived from parent-reported sleep-related breathing disorder subscale (age 5) in addition to cognitive behaviour assessments (i.e. Child Behavior Checklist (CBCL) internalizing and externalizing scores, which are associated with anxiety and aggressive behaviours, respectively). We selected 108 loci for a candidate gene analysis of both SDB and CBCL scores that included variants previously associated with schizophrenia in a genome-wide association study by the Psychiatric Genomics Consortium (PGC) [2]. In addition to main genetic effects, we investigated the potential for genetic interactions with exposures such as exclusive breastfeeding until 3 months.

**Results:** Single variant associations with CBCL externalizing score identified 14 significant variants located at chromosome 6q12 (p < 3.63 × 10^−5^). In addition, we identified 2 variants significantly associated with SDB at chromosome 6p22.1 (p = 1.8 × 10^−5^). Moreover, we identified an interaction effect between genetic variants at chromosome16q21 and exclusive breastfeeding at 3 months for CBCL externalizing score.

**Conclusion:** Our study identified that genetic variants associated with schizophrenia in adults may contribute to cognitive behavioural traits and SDB among children during early childhood. These results suggest a common genetic predisposition that can be detected early in childhood and is modifiable by environmental exposures such as breastfeeding. On-going analyses include genetic risk score analysis and gene-set association tests of rare variants. Furthermore, we will explore gene-environmental interactions using additional exposures such as parental SDB, sleep duration, apnea–hypopnea index, sleep habits, and physical activity.

**Acknowledgements:** Imputation of markers for CHILD subjects was performed by G. Pare and colleagues at McMaster University. Genomic analysis was performed with support provided by the Centre for Advanced Computing (CAC) at Queen’s University in Kingston, Ontario. The CAC is funded by: The Canada Foundation for Innovation, the Government of Ontario, and Queen’s University.

The CHILD Study was primarily funded by the Allergy, Genes and Environment (AllerGen) Network of Centres of Excellence and the Canadian Institutes of Health Research.

**References**

1. Tamana SK, Smithson L, Lau A, Mariasine J, Young R, Chikuma J, Lefebvre DL, Subbarao P, Becker AB, Turvey SE, Sears MR, CHILD Study Investigators, Pei J, Mandhane PJ. Parent-reported symptoms of sleep-disordered breathing are associated with increased behavioral problems at 2 years of age: The Canadian Healthy Infant Longitudinal Development Birth Cohort Study. Sleep. 2018;41(1).
2. Schizophrenia Working Group of the Psychiatric Genomics Consortium, Biological insights from 108 schizophrenia-associated genetic loci. Nature. 2014;511(7510):421–7.

### A4 Allergic immune dysregulation caused by a *JAK1* gain-of-function mutation

#### Catherine M. Biggs^1,2^, Kate L. Del Bel^1,2^, Robert J. Ragotte^1,2^, Aabida Saferali^1,2^, Felix Orben^1,2^, Margaret L. McKinnon^2,3^, Sara Mostafavi^3,4^, Stuart E. Turvey^1,2^

##### ^1^Department of Pediatrics, University of British Columbia, Vancouver, BC, Canada; ^2^BC Children’s Hospital Research Institute, Vancouver, BC, Canada; ^3^Department of Medical Genetics, University of British Columbia, Vancouver, BC, Canada; ^4^Department of Statistics, University of British Columbia, Vancouver, BC, Canada

###### **Correspondence:** Catherine M Biggs (cbiggs@bcchr.ca)

*Allergy, Asthma & Clinical Immunology* 2019, **15**(**Suppl 2**):A4

**Background:** Primary atopic disorders are a group of genetic conditions characterized by excessive allergic inflammation irrespective of sensitization [1]. Studying the single gene defects that cause these disorders has led to fundamental discoveries in basic and clinical immunology [2, 3]. This, in turn, has created new therapeutic targets for allergic diseases, which carry an immense health and economic burden in Canada [4, 5]. Germline gain-of-function (GOF) mutations in *JAK1* are a newly described cause of primary atopic disorder [6]. The clinical phenotype of the first and only described affected family includes severe atopic dermatitis, markedly elevated peripheral blood eosinophil counts, hepatosplenomegaly, autoimmunity, and failure to thrive [6]. We sought to characterize the mechanisms behind JAK1-mediated atopic immune dysregulation, focusing on its effect on T cell activation and differentiation.

**Methods:** Peripheral blood mononuclear cells from healthy controls and patients carrying the *JAK1* GOF mutation were evaluated by flow cytometry. T cell activation status and T helper cell subset distribution were analyzed using extracellular surface marker and intracellular cytokine staining by flow cytometry. Gene expression of RNA extracted from whole blood samples of affected patients and healthy controls was analyzed using next-generation RNA sequencing. Gene signatures corresponding to differentially expressed genes found in T helper subsets were compared between healthy controls and *JAK1* GOF patients before and after treatment with ruxolitinib, an approved JAK1/2 inhibitor.

**Results:** T cell immunophenotypic analysis by flow cytometry revealed a decreased proportion of naïve CD4^+^ T cells, in keeping with increased T cell activation. Elevated percentages of polarized T helper subsets were seen in comparison to healthy controls, with particular skewing towards the Th2 subset. RNA sequencing analysis revealed increased expression of Th1, Th2 and Th17 gene signatures in patients compared to healthy controls, with post-ruxolitinib treatment analysis showing a decline in Th2 and Th17-associated gene expression.

**Conclusions:** Hyperactive JAK1 signaling appears to enhance T cell differentiation, in particular towards T helper subsets implicated in allergic inflammation. Medications that inhibit JAK1 may have benefits in treating disorders associated with allergic immune dysregulation, such as atopic dermatitis, asthma and eosinophilia.

**References**

1. Lyons JJ, Milner JD. Primary atopic disorders. J Exp Med. 2018;215:1009–22.
2. Bennett CL, Christie J, Ramsdell F, Brunkow ME, Ferguson PJ, Whitesell L, Kelly TE, Saulsbury FT, Chance PF, Ochs HD. The immune dysregulation, polyendocrinopathy, enteropathy, X-linked syndrome (IPEX) is caused by mutations of FOXP3. Nat Genet. 2001;27:20–1.
3. Fontenot JD, Gavin MA, Rudensky AY. Foxp3 programs the development and function of CD4+CD25+ regulatory T cells. Nat Immunol. 2003;4:330–6.
4. Pawankar R. Allergic diseases and asthma: a global public health concern and a call to action. World Allergy Organ J. 2014;7:12.
5. Royce D. Knowledge translation opportunities in allergic disease and asthma. Allergy Asthma Clin Immunol. 2010;6.
6. Del Bel KL, Ragotte RJ, Saferali A, Lee S, Vercauteren SM, Mostafavi SA, Schreiber RA, Prendiville JS, Phang MS, Halparin J, et al. JAK1 gain-of-function causes an autosomal dominant immune dysregulatory and hypereosinophilic syndrome. J Allergy Clin Immunol. 2017; 139:2016–20.e2015.

### A5 Influence of simulated obstructive sleep apnea on thoracic fluid volume and airways resistance in healthy subjects

#### Xiaoshu Cao^1,2^, T. Douglas Bradley^2,3^, Swati Bhatawadekar^2^, Bojan Gavrilovic^2^, Shumit Saha^1,2^, Cristina O. Francisco^2^, Azadeh Yadollahi^1,2^

##### ^1^Institute of Biomaterials & Biomedical Engineering, University of Toronto, Toronto, Ontario, Canada; ^2^University Health Network-Toronto Rehabilitation Institute, Toronto, Ontario, Canada; ^3^Department of Medicine, University of Toronto, Toronto, Ontario, Canada

###### **Correspondence:** Xiaoshu Cao (Xiaoshu.cao@mail.utoronto.ca)

*Allergy, Asthma & Clinical Immunology* 2019, **15**(**Suppl 2**):A5

**Background:** Obstructive sleep apnea (OSA) is common in asthmatics, with a significant overlap of 12–50% [1]. OSA prevalence increases with increasing asthma severity, suggesting a pathophysiological link between the two [2]. OSA is also a risk factor for frequent nocturnal asthma exacerbations [3]. Recently, overnight rostral fluid shift (fluid displacement out of the legs into the neck and peripharyngeal tissues) was found to increase upper airway resistance and severity of OSA [4]. Recently, we have shown that in asthmatics while supine, fluid shifts from the legs into the thorax increases lower airways resistance (R_LA_) [5, 6]. During an obstructive apnea, intrathoracic pressure decreases dramatically, which could increase blood pooling into the thorax and increase thoracic fluid volume (TFV). Our objective is to determine whether in asthmatics, generation of negative pleural pressure by co-existing OSA could draw fluid into the thorax and therefore exacerbate lower airways narrowing and increase R_LA_. We hypothesized that negative pleural pressure generated by inspiratory efforts against an occluded upper airway will increase TFV. If true, the excess fluid in the thorax may increase R_LA_, worsening asthma during sleep.

**Methods:** Healthy subjects laid supine for 30 min and were randomized to a control or intervention study arm with a 1-h seated washout period between the study arms. In the control arm, subjects breathed normally. In the intervention arm, subjects performed Mueller maneuvers (MM) by breathing against occluded mouth and nose to simulate OSA. 25 MMs were performed for 15 s each at esophageal pressure of − 40 cmH_2_O followed by 15 s of normal breathing. TFV and R_LA_ were monitored continuously. Changes in TFV and R_LA_ from 0 min to 30 min were calculated and compared between study arms using repeated measures ANOVA.

**Results:** In six healthy subjects, MMs caused a significantly greater increase in TFV of 124 ml than the control arm (P = 0.0007, Fig. 1). However, R_LA_ did not change significantly during MMs compared to control (0.3 ± 1.4 vs − 0.9 ± 1.3, P = 0.16).

**Fig. 1:**
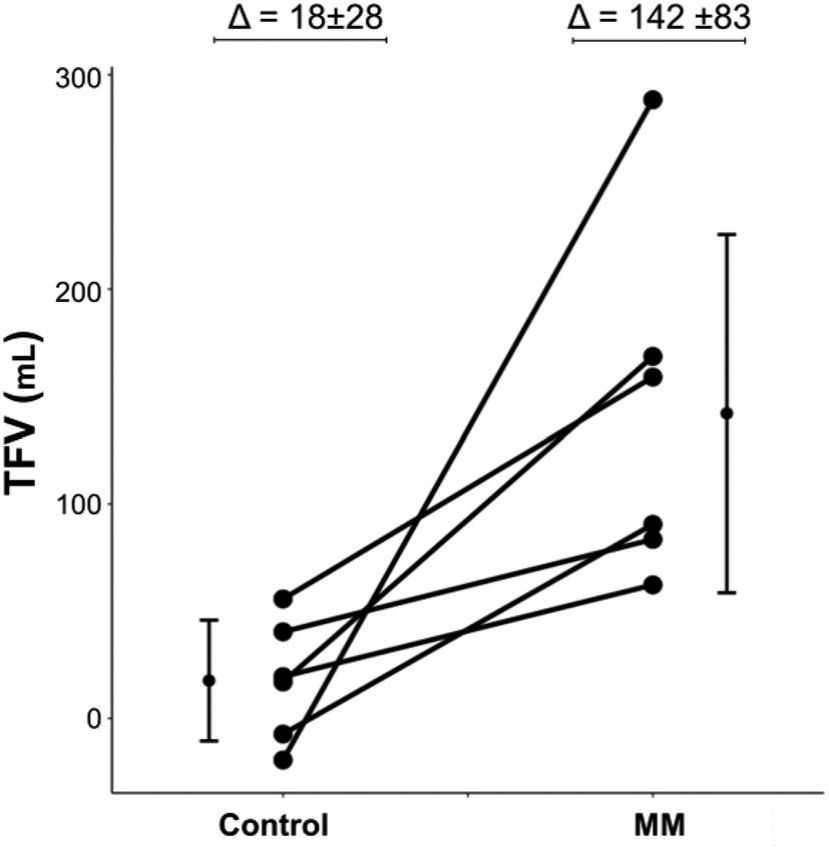
Changes in TFV during MMs and control study arms (n = 6)

**Conclusion:** The results provide strong evidence that MMs draw fluid into the thorax. However, in healthy subjects this did not affect R_LA._ Based on our previous findings that induced rostral fluid shift of 53 ml provokes increases in R_LA_ in asthmatics [5, 6], it is likely that MMs will have similar effects in asthmatics. Accordingly, we plan to test this hypothesis in asthmatics.

**References**

1. Kong DL, Qin Z, Shen H, Jin HY, Wang W, Wang ZF. Association of obstructive sleep apnea with asthma: a meta-analysis. Sci Rep. 2017;7:4008.
2. Julien JY, Martin JG, Ernst P, Olivenstein R, Hamid Q, Lemie`re C, Pepe C, Naor N, Olha A, Kimoff RJ. Prevalence of obstructive sleep apnea–hypopnea in severe versus moderate asthma. J Allergy Clin Immunol. 2009;124(2):371–6.
3. Chan CS, Woolcock AJ, Sullivan CE. Nocturnal asthma: role of snoring and obstructive sleep apnea. Am Rev Respir Dis. 1988;137:1502–4.
4. Redolfi S, Yumino D, Ruttanaumpawan P, Yau B, Su MC, Lam J, Bradley TD. Relationship between overnight rostral fluid shift and obstructive sleep apnea in nonobese men. Am J Respir Crit Care Med. 2009;179:241–6.
5. Bhatawadekar SA, Inman MD, Fredberg JJ, Tarlo SM, Lyons OD, Keller G, Yadollah A. Contribution of rostral fluid shift to intrathoracic airway narrowing in asthma. J Appl Physiol. 2017;122(4):809–16.
6. Bhatawadekar SA, et al. Reduced baseline airway caliber relates to larger airway sensitivity to rostral fluid shift in asthma. Front Physiol. 2017;8(1012).

### A6 Genome-wide association study of human milk oligosaccharides in the CHILD Study

#### Le Chang^1,2^, Amirthagowri Ambalavanan^2^, Amel Lamri^3^, Bianca Robertson^4^, Chloe Yonemitsu^4^, Stuart E. Turvey^5,6^, Piushkumar J. Mandhane^7^, Allan B. Becker^8,9^, Theo J. Moraes^10^, Sonia S. Anand^11^, Guillaume Paré^12^, Diana L. Lefebvre^11^, Malcolm R. Sears^11^, Padmaja Subbarao^10^, Lars Bode^4^, Meghan B. Azad^8,9^, Qingling Duan^1,2^

##### ^1^School of Computing, Queen’s University, Kingston, Ontario, Canada; ^2^Department of Biomedical and Molecular Sciences, Queen’s University, Kingston, Ontario, Canada; ^3^Department of Clinical Epidemiology and Biostatistics, McMaster University, Hamilton, Ontario, Canada; ^4^Department of Pediatrics and Larsson-Rosenquist Foundation Mother-Milk-Infant Center of Research Excellence, University of California San Diego, La Jolla, California, USA; ^5^Division of Allergy and Immunology, Department of Pediatrics, University of British Columbia, Vancouver, British Columbia, Canada; ^6^Department of Pediatrics, Child and Family Research Institute and British Columbia Children’s Hospital, Vancouver, British Columbia, Canada; ^7^Department of Pediatrics, University of Alberta, Edmonton, Alberta, Canada; ^8^Manitoba Developmental Origins of Chronic Diseases in Children Network (DEVOTION), Children’s Hospital Research Institute of Manitoba, Winnipeg, Manitoba, Canada; ^9^Department of Pediatrics and Child Health, University of Manitoba, Winnipeg, Manitoba, Canada; ^10^Department of Pediatrics, Hospital for Sick Children and University of Toronto, Toronto, Ontario, Canada; ^11^Department of Medicine, McMaster University, Hamilton, Ontario, Canada; ^12^Pathology and Molecular Medicine, McMaster University, Hamilton, Ontario, Canada

###### **Correspondence:** Qingling Duan (qingling.duan@queensu.ca)

*Allergy, Asthma & Clinical Immunology* 2019, **15**(**Suppl 2**):A6

**Background:** Human milk oligosaccharides (HMOs) are abundant in human breastmilk; however, they are absent from most infant formulas. HMOs shape the growth of infant gut microbiota and contribute to immune system development. Maternal secretor status and environmental factors influence HMO synthesis and concentration. While specific genetic variants in fucosyltransferase (*FUT*) genes are known to determine maternal secretor status, it remains unknown if other genetic factors modulate HMO concentrations in breastmilk [1]. The aim of this study was to investigate the association of genetic variants with the concentration of HMOs in breastmilk.

**Methods:** Our study involved a subgroup of 1206 Canadian mothers from the Canadian Healthy Infant Longitudinal Development (CHILD) birth cohort. Breastmilk samples were collected 3–4 months postpartum. A total of 19 HMOs were analyzed by high-performance liquid chromatography-mass spectrometry (HPLC–MS); secretor status was determined from the presence of 2′-fucosyllactose [2]. Genotyping data were obtained using the Illumina HumanCoreExome BeadChip and imputations were conducted using *IMPUTE2* based on sequence data from the 1000 Genomes Project. We performed a genome-wide association study (GWAS) of maternal secretor status (logistic regression) and of each HMO concentration (linear regression), which included 6 million imputed variants (minor allele frequency > 0.05). The functional effect prediction tool *SnpEff* was used for annotation of the associated single-nucleotide polymorphisms (SNPs) to determine their potential coding or regulatory functions.

**Results:** We identified significant associations for multiple loci in chromosome 19 (within the region encoding *FUT2*) with 16 HMO concentrations as well as maternal secretor status. In addition, several variants on chromosome 3 were correlated with 6′-sialyllactose (*P* = 3.92 × 10^−9^) and disialyllacto-*N*-hexaose (*P* = 1.51 × 10^−8^); the most significant SNP in this region was a genetic variant located downstream of the β-galactoside α-2,6-sialyltransferase 1 (*ST6GAL1)* gene.

**Conclusions:** Common genetic variations are strongly correlated with HMO concentrations. In addition to observing expected associations for genetic variants in the *FUT2* locus on chromosome 19, we identified several novel associations with variants on chromosome 3. Future work in our lab will investigate the genetic factors modulating other breastmilk components, and determine the long-term impact on infant health and childhood asthma.

**Acknowledgements:** This work was supported by AllerGen NCE Inc., Research Manitoba and CIHR.

**References**

1. Bode L. Human milk oligosaccharides: every baby needs a sugar mama. Glycobiology. 2012;22:1147–62.
2. Azad MB, Robertson B, Atakora F, Becker AB, Subbarao P, Moraes TJ, Mandhane PJ, Turvey SE, Lefebvre DL, Sears MR. Human milk oligosaccharide concentrations are associated with multiple fixed and modifiable maternal characteristics, environmental factors, and feeding practices. J Nutr. 2018.

### A7 Early life patterns of respiratory-related health services use in a birth cohort

#### Wenjia Chen^1,2^, Padmaja Subbarao^3,4,5^, Rachel E. McGihon^1^, Laura Y. Feldman^1^, Jingqin Zhu^1,2^, Wendy Lou^6^, Andrea S. Gershon^1,2,6,7^, Kawsari Abdullah^1^, Theo J. Moraes^3,4^, Aimée Dubeau^3^, Malcolm R. Sears^5^, Diana L. Lefebvre^5^, Stuart E. Turvey^8^, Piush J. Mandhane^9^, Meghan B. Azad^10^, Teresa To^1,2,6^

##### ^1^Child Health Evaluative Sciences, The Hospital for Sick Children, Toronto, Canada; ^2^Institute for Clinical Evaluative Sciences, Toronto, Canada; ^3^Translational Medicine and Division of Respiratory Medicine, The Hospital for Sick Children, Toronto, Canada; ^4^Department of Pediatrics, University of Toronto, Canada; ^5^Department of Medicine, McMaster University, Hamilton, Canada; ^6^Dalla Lana School of Public Health, University of Toronto, Toronto, Canada; ^7^Sunnybrook Health Sciences Centre, Toronto, Canada; ^8^Department of Pediatrics, BC Children’s Hospital, Vancouver, Canada; ^9^Department of Pediatrics, University of Alberta, Edmonton, Canada; ^10^Department of Pediatrics & Child Health, Children’s Hospital Research Institute of Manitoba, University of Manitoba, Winnipeg, Canada

###### **Correspondence:** Wenjia Chen (chwenjia@gmail.com)

*Allergy, Asthma & Clinical Immunology* 2019, **15**(**Suppl 2**):A7

**Background:** Knowledge of the pattern of respiratory-related health care utilization in early childhood may help identify children at high risk of respiratory morbidities. This study aimed to identify distinctive patterns of respiratory-related health services use (HSU) between birth and 3 years of age.

**Methods:** We conducted a longitudinal analysis on a sub-cohort enrolled in the Toronto site of the Canadian Healthy Infant Longitudinal Development (CHILD) birth cohort study in 2009–2012. We included 729 mother and child pairs of this sub-cohort who were linked to Ontario health administrative databases (2009–2016) using unique personal health card numbers. Based on the individual trajectories of hospitalization, emergency department (ED) and physician office visits for respiratory conditions and total healthcare costs, we performed a cluster analysis to identify distinct groups of children who followed a similar pattern of respiratory-related HSU between birth and 3 years of age. Finally, we compared the distributions of early respiratory symptoms and health characteristics across HSU groups, and examined group-specific future healthcare costs between 3 and 5 years of age.

**Results:** Two statistically significant distinct HSU pattern groups were identified. The majority (N = 678, frequency weight = 0.905) showed a pattern of low and stable respiratory care utilization (Low HSU) while the remainder (N = 51, weight = 0.095) showed a pattern of high and varying utilization (High HSU) (Fig. 1). From 0 to 3 years of age, the Low- and High-HSU groups differed in their mean trajectories of total healthcare costs ($783 per 6 months decreased to $114, versus $1796 to $177, respectively). Compared to Low-HSU, the High-HSU group was associated with a constant risk of respiratory-related hospitalizations and early high ED utilization and physician visits for respiratory problems. The two groups differed significantly in the timing of wheezing (late onset in Low-HSU versus early in High-HSU), future total costs (stable versus increased), prevalence of atopy and parental socioeconomic status.

**Fig. 1:**
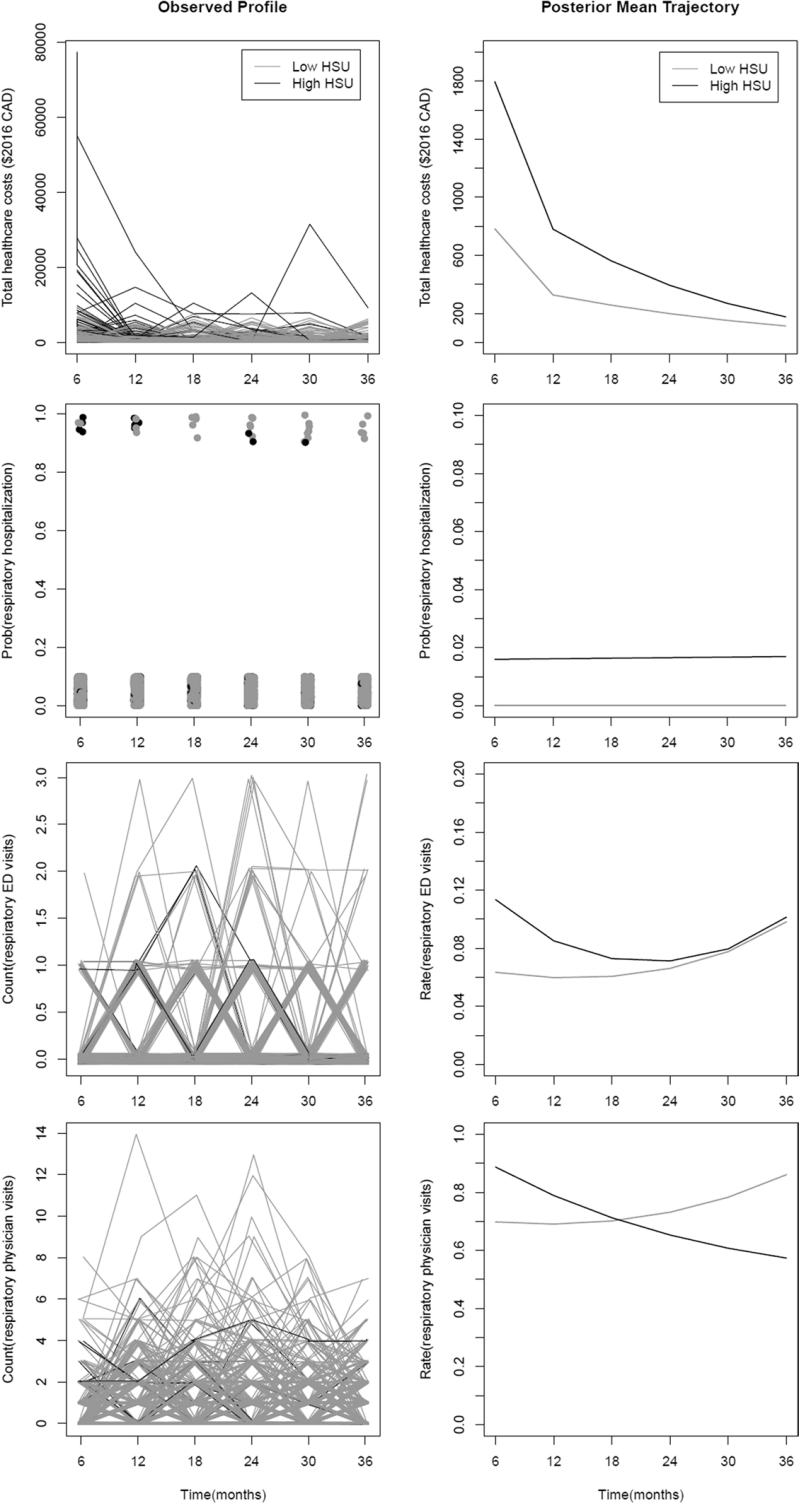
Distinct patterns of respiratory-related HSU between 0 and 3 years of age. Right panel shows the model-estimated posterior mean trajectory of each pattern. Left panel shows the observed longitudinal profiles of each pattern

**Conclusions:** We identified distinct patterns of respiratory-related HSU in early life that may be associated with subsequent respiratory-related morbidity. These patterns may help identify at risk children in a large population and inform health resource allocation.

### A8 Genetic risk score analysis to identify polygenic variations for predicting recurrent wheeze in the Canadian Healthy Infant Longitudinal Development Study

#### Jihoon Choi^1^, Christoffer Dharma^2^, Ruixue Dai^3^, Amel Lamri^4^, Amirthagowri Ambalavanan^1^, Sonia S. Anand^2^, Guillaume Pare^5^, Diana L. Lefebvre^2^, Stuart E. Turvey^6^, Piush J. Mandhane^7^, Allan B. Becker^8^, Meghan B. Azad^8^, Theo J. Moraes^3,9^, Malcolm R. Sears^2^, Padmaja Subbarao^3,9,10^, Qingling Duan^1,11^

##### ^1^Department of Biomedical and Molecular Sciences, Queen’s University, Kingston, Ontario, Canada; ^2^Department of Medicine, McMaster University, Hamilton, Ontario, Canada; ^3^The Hospital for Sick Children, Toronto, Ontario, Canada; ^4^Department of Clinical Epidemiology and Biostatistics, McMaster University, Hamilton, Ontario, Canada; ^5^Pathology and Molecular Medicine, McMaster University, Hamilton, Ontario, Canada; ^6^Division of Allergy and Immunology, Department of Pediatrics, University of British Columbia, BC, Canada; ^7^Division of Pediatric Respiratory Medicine, University of Alberta, Alberta, Canada; ^8^Department of Pediatrics and Child Health, University of Manitoba, Manitoba, Canada; ^9^Department of Paediatrics, University of Toronto, Toronto, Ontario, Canada; ^10^Division of Respirology, Department of Medicine, McMaster University, Hamilton, Ontario, Canada; ^11^School of Computing, Queen’s University, Kingston, Ontario, Canada

###### **Correspondence:** Qingling Duan (qingling.duan@queensu.ca)

*Allergy, Asthma & Clinical Immunology* 2019, **15**(**Suppl 2**):A8

**Background:** Earlier studies estimated that genetic factors contribute to 55–74% of asthma heritability, of which only a small fraction may be explained by known genetic loci [1]. We hypothesized that the missing heritability lies partially within the effects of polygenic variations, which are alleles with small effect sizes that standard genome-wide association studies (GWAS) are underpowered to detect. Using genomics data from the Canadian Healthy Infant Longitudinal Development Study (CHILD; N = 3455) [2], we investigated the aggregated effect posed by a novel set of polygenic variations with modifiable exposures on risk of recurrent wheeze during early childhood.

**Methods:** Genome-wide single nucleotide polymorphism data were ascertained from mother–child pairs of the CHILD Study using the Illumina HumanCoreExome BeadChip. After quality control assessments and imputations, 22 million variants from 2830 children were included for analysis. Our primary outcome was recurrent wheeze from age 2 to 5 years, which is strongly correlated with childhood asthma. In our genetic risk score (GRS) analysis, we used summary statistics from the largest asthma GWAS to date [3], selecting significantly associated SNPs (p < 5 × 10^−8^) followed by pruning of variants in linkage disequilibrium (LD; r^2^ > 0.6). We tested the combined effect of asthma-associated variants with recurrent wheeze in the CHILD cohort to identify a set of risk variants that is predictive of recurrent wheeze. This was done through forward selection of variants by their strength of association, while using coefficients from the summary statistics as weights.

**Results:** We identified a novel set of 19 variations that effectively predicted recurrent wheeze in the CHILD cohort (Fig. 1a). In addition, we observed that breastfeeding at 12 months, a modifiable exposure variable, was protective against wheeze, particularly among children with lower genetic risk (Fig. 1b).

**Fig. 1:**
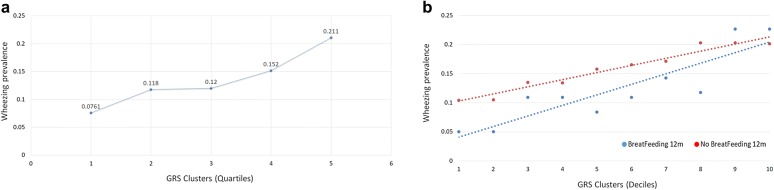
**a** Changes in wheeze prevalence by the deciles of genetic risk score estimated based on 19 risk variants identified in this study. Increase in wheeze prevalence is strongly associated with increase in genetic risk score (pval: 1.38e−10). **b** Comparison between breastfed and non-breastfed group at 12 months show that wheeze prevalence is higher among those with lower genetic risk vs. those who were breastfed. However, the beneficial effects of breastfeeding are not evident among those subjects with the highest genetic risk

**Conclusions:** Our results show that both polygenetic factors and environmental exposures contribute to recurrent wheeze in children as young as age 2, which is associated with asthma diagnosis later in childhood. On-going analyses include examining other asthma-related phenotypes such as longitudinal lung function and allergen sensitization, as well as environmental variables such as pet ownership and nutrition.

**Acknowledgements:** Pre-processing of genotype data was done by G. Pare, S.S. Anand and colleagues at McMaster University. Computations were performed with support provided by the Centre for Advanced Computing (CAC) at Queen’s University. The CAC is funded by: The Canada Foundation for Innovation, the Government of Ontario, and Queen’s University. J.C. receives funding from the Canadian Institutes of Health Research.

**References**

1. Vicente CT, Revez JA, Ferreira MAR. Lessons from ten years of genome-wide association studies of asthma. Clin Transl Immunol. 2017;6:e165.
2. Subbarao P, Anand SS, Becker AB, Befus AD, Brauer M, Brook JR, Denburg JA, HayGlass KT, Kobor MS, Kollmann TR, et al. The Canadian Healthy Infant Longitudinal Development (CHILD) Study: examining developmental origins of allergy and asthma. Thorax. 2015;70:998–1000.
3. Demenais F, Margaritte-Jeannin P, Barnes KC, Cookson WOC, Altmuller J, Ang W, Barr RG, Beaty TH, Becker AB, Beilby J, et al. Multiancestry association study identifies new asthma risk loci that colocalize with immune-cell enhancer marks. Nat Genet. 2018;50:42–53.

### A9 The effects of participant engagement and knowledge translation in a pediatric longitudinal research study: Insights from the Vancouver site of the CHILD study

#### Tamara Coffin^1^, Lauren Muttucomaroe^1^, Linda Warner^1^, Maureen Mooney^1^, Mary Beckingham^1^, Diana Lefebvre^2^, Padmaja Subbarao^3^, Malcolm Sears^2^, Meghan Azad^4^, Stuart Turvey^1^

##### ^1^Department of Pediatrics, Faculty of Medicine, The University of British Columbia, BC Children’s Hospital, Vancouver, British Columbia, Canada; ^2^Department of Medicine, McMaster University, Hamilton, Ontario, Canada; ^3^Department of Pediatrics, Hospital for Sick Children, University of Toronto, Toronto, Ontario, Canada; ^4^Department of Pediatrics & Child Health and Community Health Sciences, University of Manitoba, Winnipeg, Manitoba, Canada

###### **Correspondence:** Tamara Coffin (tamara.coffin@bcchr.ca)

*Allergy, Asthma & Clinical Immunology* 2019, **15**(**Suppl 2**):A9

**Background:** Participant engagement in clinical research is critical in optimizing health outcomes by providing benefits for the participants and families. These benefits range from improved quality and efficiency of care to general improvement in population health [1]. Different forms and levels of engagement include consultation, partnerships, organizational design and policy making [1]. This abstract will focus on the Vancouver site’s experience, as part of a nation-wide initiative.

**Research question:** Will participant engagement strategies maximize retention rates at the CHILD Study Vancouver site?

**Methods:** The CHILD Study uses knowledge translation (KT) strategies as a tool in participant engagement, which also enhances effective retention strategies [2]. These include sending birthday cards and regular newsletters, and keeping the CHILD website current. The CHILD Study has developed a nation-wide initiative around participant engagement facilitated by the Knowledge Mobilization Stakeholder Advisory Committee. At the Vancouver site, participant-wide feedback surveys, the establishment of focus groups and a town hall meeting encouraged family engagement and increased KT. The formation of a participant advisory council (PAC) allows for collaboration and a partnership with the study team.

**Results:** The creation of focus groups encouraged parents to provide both positive and negative feedback during the planning stage of the 8-year visit. The ‘Town Hall’ celebration marked the end of the 5-year visits and provided a space to share research and for participants to discuss common ideas with other families and the entire CHILD Study team. The goal of PACs is to create a study environment meaningful to parents and ultimately the children as they grow. Parent voices generated feedback to help co-develop key indicators for study success. The PAC meetings helped shape the structure and flow of the 8-year visits. The feedback surveys provided a more representative opinion of the participants, with a 66% response rate. This participant engagement strategy has been a contributing factor in the retention rates of 95.7%, 93.2%, and 93.0% at 1, 3, and 5 years, respectively. Overall, these patient engagement and KT strategies have humanized research and empowered the families within their care.

**Conclusions:** The PAC demonstrates an effective participant engagement and KT strategy that will help shape the 11 and 14-year visits. A longer term goal is to eventually involve the children as equal participant stakeholders and practice engagement with the pediatric participants themselves.

**References**

1. Carman KL, Dardess P, Maurer M, Sofaer S, Adams K, Bechtel C, Sweeney J. Patient and family engagement: a framework for understanding the elements and developing interventions and policies. Health Affairs. 2013;32(2):223–31.
2. Zook PM, Jordan C, Adams B, Visness CM, Walter M, Pollenz K, D’Agostino J. Retention strategies and predictors of attrition in an urban pediatric asthma study. Clin Trials. 2010;7(4):400–10.

### A10 Evaluating new strategies towards reducing the allergenicity of peanuts using nuclear magnetic resonance (NMR) spectroscopy

#### Casey G. Cohen, Wei Zhao, Bertrand J. Jean-Claude, Bruce D. Mazer

##### Research Institute of the McGill University Health Centre, Montreal, Quebec, Canada

###### **Correspondence:** Casey G. Cohen (casey.cohen2@mail.mcgill.ca)

*Allergy, Asthma & Clinical Immunology* 2019, **15**(**Suppl 2**):A10

**Background:** Peanut allergy is considered the most severe of all food allergies as it is the leading cause of fatal anaphylaxis. An estimated 1–2% of the North American population suffers from peanut allergy. Previous studies suggested that the allergenicity of peanuts is significantly increased in roasted peanuts when compared with raw [1]. Our project aimed to develop alternative processing methods to decrease the allergenicity of peanuts. We therefore undertook a nuclear magnetic resonance (NMR) approach to establish small molecule signatures of mixtures derived from roasted and raw peanuts.

**Methods:** Advanced Glycation End products (AGEs) are considered to be the main cause of increased allergenicity in peanuts following roasting. We first used High-Resolution Magic Angle Spinning (HR-MAS) and solution ^1^H NMR to take snapshots of the carbohydrate signatures of the peanuts under different conditions. Peanuts were ground into a paste and dissolved in hexane to remove the lipid content, and then both untreated peanut and defatted peanut were analyzed by HR-MAS ^1^H NMR. Raw and roasted peanuts were soaked in double distilled water and the resulting solutions were characterized by ^1^H NMR. Protein extracts from raw, roasted (150 °C, 30 min) and boiled (100 °C, 2 h in water) peanuts were used to quantify IgE binding via competitive ELISA using serum samples from peanut-sensitive patients.

**Results:** Differences between raw and roasted whole peanuts in the solid state could not be detected by HR-MAS NMR due to the dominance of the triglyceride peaks in the spectrum. Defatting of the peanut prior to HR-MAS NMR analysis revealed significant differences between the small molecule profiles of both raw and roasted peanut. In the peanut-soaked solutions, significant differences were also observed in the sugar pattern, with sucrose clearly being the dominant species in the raw peanut, but with glucose being dominant in the roasted peanut. Competitive IgE binding assay of the extracts revealed no difference in IgE binding between raw, roasted and boiled peanuts.

**Conclusions:** The results suggest that NMR spectroscopy is a useful tool for determining small molecule profile differences between different states of the peanut. However, in contrast to other findings, our results indicate no relevant difference in IgE binding between conditions of non-thermally (raw) and thermally (roasted and boiled) processed peanuts.

**Acknowledgements:** This work was supported by AllerGen NCE Inc. (the Allergy, Genes and Environment Network), a member of the Networks of Centres of Excellence Canada program.

**Reference**

1. Maleki SJ, Chung SY, Champagne ET, Raufman JP. The effects of roasting on the allergenic properties of peanut proteins. J Allergy Clin Immunol. 2000;106(4): 763–8.

### A11 Early-life gut microbiome dysbiosis in childhood atopic dermatitis

#### Chelsea Cutler^1^, Hind Sbihi^1^, Darlene Dai^1^, Charisse Petersen^1^, Rozlyn C.T. Boutin^1^, Malcolm R. Sears^2^, Padmaja Subbarao^3^, Theo J. Moraes^3^, Allan B. Becker^4^, Meghan B. Azad^4^, Piush J. Mandhane^5^, B. Brett Finlay^1^, Stuart E. Turvey^1^

##### ^1^University of British Columbia, Vancouver, B, Canada; ^2^McMaster University, Hamilton, ON, Canada; ^3^Hospital for Sick Children, Toronto ON, Canada; ^4^University of Manitoba, Winnipeg, MB, Canada; ^5^University of Alberta, Edmonton, AB, Canada

###### **Correspondence:** Stuart E. Turvey (sturvey@bcchr.ca)

*Allergy, Asthma & Clinical Immunology* 2019, **15**(**Suppl 2**):A11

**Background:** Understanding connections between the gut microbiome and immunity continues to be an important area of study in allergic disorders. The ‘atopic march’ paradigm suggests a progression of allergic disorders over time, starting with atopic dermatitis (AD). An infant’s gut microbiome is established throughout the first year of life, impacted by factors such as mode of delivery, breastfeeding, antibiotic use, and introduction of foods^1^. Around 3 years of age, the gut microbiome stabilizes [1, 2]. Infants with a less mature, or less diverse, gut microbiome structure appear to have a higher risk of developing allergic disease later in childhood [2–4]. This suggests deliberate therapeutic modification of the gut microbiome may offer protection against development of allergic disease. However, before developing strategies to modify the microbiome, it is necessary to know the composition of the gut microbiome in children with and without atopic dermatitis. This study aims to compare the early-life gut microbiome composition between preschool children with atopic dermatitis and healthy controls.

**Materials and methods:** To assess the early-life gut microbiome, we analysed 1736 stool samples from 1000 infants involved in the Canadian Healthy Infant Longitudinal Development (CHILD) Study. Stool samples were collected at age 3 months (n = 857) and 1 year (n = 879). Clinical assessments were performed at ages 1, 3 and 5 years, which included an assessment of AD by an experienced health-care professional using the UK Working Party criteria. This study provides a comprehensive analysis of metadata and16S rRNA gene sequencing from the stool samples.

**Preliminary results:** Confirming the known maturation of the gut microbiome with age, we found significant differences in alpha diversity (p < 2.2e−16) when comparing 3-month stool samples to 1-year stool samples. There was significantly more *Bifidobacterium* (p = 2.57e−06) and significantly less Bacteroidetes (p = 6.22e−10) and Firmicutes (p = 1.09e−15) in 3-month stools than 1-year stools. Children diagnosed with atopic dermatitis at age 5 years had significantly lower diversity in both 3-month (n = 125, p = 0.03) and 1-year samples (n = 126, p = 0.03) compared to healthy controls (3-month samples n = 550, 1-year samples n = 540).

**Conclusions:** Significant differences in diversity are seen in the gut microbiome of children with AD and healthy children. Identifying if abundances of specific bacteria differ between healthy children and those with AD is our next step. Characterizing the early-life gut microbiome of children who develop AD is a crucial component in creating live biotherapeutics designed to prevent or treat AD and related allergic diseases.

**References**

1. Tanaka M, Nakayama J. Development of the gut microbiota in infancy and its impact on health in later life. Allergol Int. 2017;66(4):515–22.
2. Wopereis H, Oozeer R, Knipping K, Belzer C, Knol J. The first thousand days—intestinal microbiology of early life: establishing a symbiosis. Pediatr Allergy Immunol. 2014;25(5):428–38.
3. Stokholm J, Blaser MJ, Thorsen J, et al. Maturation of the gut microbiome and risk of asthma in childhood. Nat Commun. 2018;9(1):141.
4. Forno E, Onderdonk AB, McCracken J, et al. Diversity of the gut microbiota and eczema in early life. Clin Mol Allergy. 2008;6:11.

### A12 Early-life antibiotic exposure, the gut microbiome, and the risk of childhood asthma: data from the CHILD cohort study

#### Darlene Dai^1,2^, Hind Sbihi^1,2^, Geoffrey L. Winsor^3^, Leah Stiemsma^4^, Rozlyn C.T. Boutin^5^, Charisse Petersen^5^, Chelsea J. Cutler^1^, Malcolm R. Sears^6^, Padmaja Subbarao^7,8^, Theo J. Moraes^7,8^, Allan B. Becker^9^, Meghan B. Azad^9^, Piush J. Mandhane^10^, Fiona S. Brinkman^3^, B. Brett Finlay^5^, Stuart E. Turvey^1,2^

##### ^1^BC Children’s Hospital Research Institute, Vancouver, BC, Canada; ^2^Department of Pediatrics, Faculty of Medicine, University of British Columbia, Vancouver, BC, Canada; ^3^Department of Molecular Biology and Biochemistry, Faculty of Health Sciences, Simon Fraser University, Burnaby, BC, Canada; ^4^Pepperdine University, Malibu, CA, USA; ^5^Department of Biochemistry and Molecular Biology, Faculty of Medicine, University of British Columbia, Vancouver, BC, Canada; ^6^Department of Medicine, Faculty of Health Sciences, McMaster University, Hamilton, ON; ^7^Department of Paediatrics, University of Toronto, Toronto, ON, Canada; ^8^Respiratory Medicine, the Hospital for Sick Children, Toronto, ON, Canada; ^9^Department of Pediatrics & Child Health and Community Health Sciences, University of Manitoba, Winnipeg, MB, Canada; ^10^Department of Pediatrics, Faculty of Medicine and Dentistry, University of Alberta, Edmonton, AB, Canada

###### **Correspondence:** Darlene Dai (darlene.dai@bcchr.ca)

*Allergy, Asthma & Clinical Immunology* 2019, **15**(**Suppl 2**):A12

**Background:** Many early-life environmental factors of Westernized lifestyle are risk factors for asthma and support a common theme of decreased early-life microbial exposure, which may contribute to hypersensitivity diseases in childhood [1]. Several of these factors (e.g. antibiotics) are potentially modifiable, yet have not been addressed despite the continued rise in asthma prevalence [2] and mounting evidence in support of exposure to beneficial microbes in infancy [3]. Here, we examine the associations between early-life antibiotic use, the structure of the gut microbiome, and risk of childhood asthma.

**Methods:** We studied 2644 children from the Canadian Healthy Infant Longitudinal Development (CHILD) Study, who were clinically assessed for asthma at age 5 years. In an asthma-enriched subset (n = 1000), gut microbiota was defined by 16SrRNA sequencing of stool samples collected at 3 and 12 months of age. Conditional logistic regressions (stratified for study center) were performed to evaluate the association between systemic antibiotic use in the first 12 months of life and the diagnosis of definite asthma at age 5 years. Generalized linear mixed effect models were applied to define associations between microbiome diversity and asthma. All analyses were adjusted for sex, parity, mode of delivery, birth weight, birth season, breastfeeding, parental atopy and ethnicity.

**Results:** 25.1% of the children were born by caesarean section and 19.9% received systemic antibiotics in the first year of life. 103 children who received antibiotics for respiratory indications were excluded to reduce the risk of reverse causality. Antibiotics before age 1 year (adjusted odds ratio [aOR]: 1.8, p = 0.006), birth by C-section without labor (aOR compared to vaginal: 1.74, p = 0.034), 1 year gut microbiota alpha diversity (aOR: 0.64, p = 0.023) and atopy of mother (OR: 2.04, p = 0.022) were significantly associated with a diagnosis of definite asthma at age 5 years. Gut microbiome diversity at age 1 year was significantly associated with antibiotic use (coefficient = − 0.25, p < 0.001), having an older sibling (coefficient = 0.21, p < 0.001) and breastfeeding at age 6 months (coefficient = − 0.2, p = 0.009 and − 0.28, p = 0.003, for non-exclusive and exclusive respectively).

**Conclusion:** Systemic antibiotic use in the first year of life is associated with both reduced gut microbial diversity at age 1 year and increased risk of asthma diagnosis at age 5 years. Lower microbial diversity can be attributed beneficial (breastfeeding) and deleterious (antibiotics) associations.

Many risk factors identified are modifiable through evidence-based actions, including decreasing early-life antibiotic use, facilitating vaginal delivery, and supporting early exposure to beneficial microbes.

**References**

1. Stiemsma LT, Reynolds LA, Turvey SE, Finlay BB. The hygiene hypothesis: current perspectives and future therapies. ImmunoTargets Ther. 2015;4:143–57.
2. Masoli M, Fabian D, Holt S, Beasley R. Global Initiative for Asthma (GINA) Program. The global burden of asthma: executive summary of the GINA Dissemination Committee Report. Allergy. 2004;59.
3. Stiemsma LT, Turvey SE. Asthma and the microbiome: defining the critical window in early life. Allergy Asthma Clin Immunol. 2017;13:3.

### A13 Using latent class analysis to identify childhood wheeze phenotypes from birth to age 5 years

#### Ruixue Dai^1^, Maxwell M. Tran^1^, Theo J. Moraes^1^, Wendy Y.W. Lou^2^, Allan B. Becker^3^, Piush J. Mandhane^4^, Stuart E. Turvey^5^, Malcolm R. Sears^6^, Padmaja Subbarao^1^

##### ^1^Department of Pediatrics, University of Toronto & Hospital for Sick Children, Toronto, Ontario, Canada; ^2^Dalla Lana School of Public Health, University of Toronto, Toronto, Ontario, Canada; ^3^Department of Pediatrics & Child Health, University of Manitoba, Winnipeg, Manitoba, Canada; ^4^Department of Pediatrics, University of Alberta, Edmonton, Alberta, Canada; ^5^Department of Pediatrics, University of British Columbia, Vancouver, British Columbia, Canada; ^6^Department of Medicine, McMaster University, Hamilton, Ontario, Canada

###### **Correspondence:** Padmaja Subbarao (padmaja.subbarao@sickkids.ca)

*Allergy, Asthma & Clinical Immunology* 2019, **15**(**Suppl 2**):A13

**Background:** Past birth cohort studies have derived longitudinal wheeze phenotypes that may account for heterogeneity in atopy, asthma, and lung function [1–3]. We used a data-driven approach to identify wheeze phenotypes from birth to age 5 years in the Canadian Healthy Infant Longitudinal Development (CHILD) Study, a multicenter, general population-based birth cohort.

**Methods:** Data on wheeze were prospectively collected at 9 time points between birth and age 5 years. Wheeze was defined by a positive parent response to, “In the last 3/6/12 months, has your child had a wheezing noise coming from his/her chest either with or without a cold?” Latent class analysis was used to derive phenotypes based on the longitudinal prevalence of wheeze. Weighted logistic regression models were used to examine associations between risk factors and phenotypes, and between phenotypes and clinical outcomes including atopy (positive skin prick test to ≥ 1 allergen) and diagnosed asthma at 3 and 5 years.

**Results:** Using data from 3154 children with wheeze data at ≥ 2 time points, five classes were identified: no wheeze (71.50%), transient-early (6.31%), late-onset (9.73%), intermediate-onset (9.64%), and persistent wheeze (2.82%). Compared to no wheeze, persistent wheeze had the strongest associations with maternal asthma (OR 3.33, 95% CI 2.19–5.06) and prenatal smoke exposure (OR 2.21, 95% CI 1.36–3.61). Late-onset and persistent wheeze were both significantly associated with atopy at age 5 years (OR 2.42, 95% CI 1.83–3.19; OR 1.88, 95% CI 1.14–3.12, respectively). Persistent wheeze had the strongest association with asthma at ages 3 (OR 71.59, 95% CI 39.41–130.05) and 5 years (OR 46.15, 95% CI 25.36–83.99).

**Conclusions:** Five wheeze phenotypes were identified in the CHILD Study to age 5 years: no wheeze, transient-early, intermediate-onset, late-onset, and persistent wheeze. Persistent wheeze had the strongest associations with asthma at ages 3 and 5 years.

**Acknowledgements:** The authors are grateful to the families who participated in the study, CHILD Study staff and investigators, and the Allergy, Genes and Environment (AllerGen) Network of Centres of Excellence.

**References**

1. Martinez F, Wright A, Taussig L, et al. Asthma and wheezing in the first six years of life. N Engl J Med. 1995;332(3):133–8.
2. Henderson J, Granell R, Heron J, et al. Associations of wheezing phenotypes in the first 6 years of life with atopy, lung function and airway responsiveness in mid-childhood. Thorax. 2008;63(11):974–80.
3. Savenije OE, Granell R, Caudri D, et al. Comparison of childhood wheezing phenotypes in 2 birth cohorts: ALPSAC and PIAMA. J Allergy Clin Immunol. 2011;127(6):1505–12.

### A14 Toll-like receptor 2 deficiency alters breast milk components and impacts the development of oral tolerance in mice

#### Bassel Dawod^1^, Alexander Edgar^2^, Jean S. Marshall^1,2^

##### ^1^Department of Pathology, Dalhousie University, Halifax, NS, Canada; ^2^Department of Microbiology & Immunology, Dalhousie University, Halifax, NS, Canada

###### **Correspondence:** Bassel Dawod (Dawod@dal.ca)

*Allergy, Asthma & Clinical Immunology* 2019, **15**(**Suppl 2**):A14

**Background:** The role of breastfeeding in the development of allergic diseases is controversial. Immunomodulatory mediators in breast milk include: cytokines, chemokines, growth factors, microbiota, and soluble receptors. Soluble toll-like receptor-2 (sTLR2), capable of regulating the innate immune system, is found in breast milk but its role in oral tolerance development is still unknown.

**Hypothesis:** We hypothesise that mothers deficient in TLR2 do not support the normal development of oral tolerance in infants due to changes in breast milk components.

**Methods:** A murine cross-fostering model was designed to evaluate the impact of maternal TLR2 and breast milk-associated factors on tolerance development. Pups from crosses of male TLR2^−/−^ with female wild type mice and male wild type crossed with female TLR2^−/−^ mice were divided into two groups, such that half of each litter remained with its biological mother while the other half was fostered by a mother of the alternate genotype. The pups were fed 20 µg/ml ovalbumin (day 10–17) to induce oral tolerance, assessed by measuring serum anti-OVA IgE responses following i.p. OVA immunisation and levels of T-regulatory cells (Tregs) in mucosal lymphoid tissues after weaning. The levels of key immune mediators in milk from of TLR2^−/−^ and wild type mothers was also examined.

**Results:** Feeding of pups by TLR2 deficient mothers impacted the normal development of oral tolerance to OVA. This was marked by elevations in anti-OVA IgE (*P *= 0.0065) in serum, reduced Tregs (*P *= 0.0477), and increased intestinal permeability, regardless of their in utero exposure to maternal TLR2. TLR2 deficiency also impacted the milk content of immunomodulatory cytokines.

**Conclusions:** Our results confirm an important role for TLR2 in the development of oral tolerance in early life, via a breast milk-dependent mechanism that may involve altered intestinal permeability.

**Funding source:** Supported by CIHR and AllerGen NCE

### A15 When and how anaphylaxis cases reach the emergency department: findings from the Cross-Canada anaphylaxis study

#### Sofianne Gabrielli^1^, Ann E Clarke^2^, Judy Morris^3^, Harley Eisman^4^, Jocelyn Gravel^5^, Rodrick Lim^6^, Sharon Lee^7^, Jennifer Gerdts^8^, Sébastien La Vieille^9,10^, Xun Zhang^11^, Moshe Ben-Shoshan^1^

##### ^1^Division of Pediatric Allergy and Clinical Immunology, Department of Pediatrics, McGill University Health Centre, Montreal, Quebec, Canada; ^2^Division of Rheumatology, Department of Medicine, Cummings School of Medicine, University of Calgary, Calgary, Alberta, Canada; ^3^Department of Emergency Medicine, Sacré-Coeur Hôpital, Montreal, Quebec, Canada; ^4^Department of Emergency Medicine, Montreal Children’s Hospital, McGill University Health Centre, Montreal, Quebec, Canada; ^5^Division of Emergency Medicine, Department of Pediatrics, Centre hospitalier universitaire Sainte-Justine, Montreal, Quebec, Canada; ^6^Division of Pediatrics and Emergency Medicine, Children’s Hospital at London Health Science Centre, London, Ontario, Canada; ^7^Executive Director, Allergy/Asthma Information Association Canada, Toronto, Ontario, Canada; ^8^Executive Director, Food Allergy Canada, Toronto, Ontario, Canada; ^9^Food Directorate, Health Canada, Ottawa, Ontario, Canada; ^10^Département sciences des aliments, Faculté des sciences de l’agriculture et de l’alimentation, Université Laval, Québec City, Québec, Canada; ^11^Centre for Outcomes Research and Evaluation, Research Institute of McGill University Health Centre, Montreal, Quebec, Canada

###### **Correspondence:** Sofianne Gabrielli (sofiannegabrielli@gmail.com)

*Allergy, Asthma & Clinical Immunology* 2019, **15**(**Suppl 2**):A15

**Background:** Anaphylaxis is a life-threatening allergic reaction requiring prompt management. Data on time interval and transportation methods to the Emergency Department (ED) for patients with anaphylaxis is scarce. We aimed to assess the percentage of patients arriving to the ED in less than 1 h and the percentage of individuals transported by ambulance and aimed to estimate associated factors.

**Methods:** Between 2011 and 2018, children presenting to four EDs in two Canadian provinces (Quebec and Ontario) with anaphylaxis were recruited as part of the Cross-Canada Anaphylaxis Registry. A standardized data form documenting symptoms and triggers of anaphylaxis was collected. Consenting patients completed an additional questionnaire querying on time and way of arrival to the ED. Multivariate logistic regression was used to estimate factors associated with arrival time and transport to the ED.

**Results:** Over a 7-year period, 421 patients with anaphylaxis were recruited from four centers in Ontario and Quebec. The majority of patients reached the ED in less than 1 h and almost half were driven to the ED by a family member (Table 1). In Quebec compared to Ontario, more cases reached the ED after 1 to 3 h (difference: 8% [95% CI 2%, 15%]) and more patients were driven by ambulance (difference: 10% [95% CI − 2%, 22%]). Arrival to the ED in less than 1 h was associated with reactions to peanuts (adjusted Odds Ratio (aOR) 1.12 [95% CI 1.03, 1.23]) while adjusting for age, sex, reaction location, asthma, reaction severity, and use of epinephrine auto-injector/antihistamines prior to arrival to the ED. Transport to the ED by ambulance was more likely when reactions occurred at school (aOR 1.18 [95% CI 1.05, 1.33]) and in cases treated with epinephrine auto-injector prior to ED arrival (aOR 1.36 [95% CI 1.23, 150] while adjusting for age, sex, reaction severity, and asthma.

**Table 1:** Characteristics of pediatric patients transported to the ED with anaphylaxis

Variable (%, 95% CI)	N = 421
Province	
Quebec	79.1% (74.8%, 82.8%)
Ontario	20.9% (17.2%, 25.2%)
Prospective	79.6%
Age at reaction (median, IQR)	4.5 (1.5, 10.6)
Sex (% males)	62.2% (57.4%, 66.8%)
Trigger	
Food	84.1% (73.5%, 80.7%)
Venom	4.3% (0.4%, 7.6%)
Drug	6.2% (2.2%, 9.3%)
Other	6.2% (2.2%, 9.3%)
Unknown	8.6% (4.3%, 11.5%)
Location of reaction	
Home	55.8% (51.0%, 60.8%)
School/daycare	20.2% (15.5%, 25.3%)
Work	0% (0.0%, 5.1%)
Restaurant	4.2% (0.0%, 9.3%)
Other	17.5% (12.8%, 22.6%)
Unknown	2.5% (0.0%, 7.6%)
Time to reach healthcare facility	
Less than 1 h	86.9% (84.0%, 90.1%)
Between 1 and 3 h	10.4% (7.4%, 13.6%)
More than 3 h	1.7% (0.0%, 4.9%)
Unknown	1.0% (0.0%, 4.2%)
Brought to healthcare facility by	
Ambulance	40.8% (35.9%, 46.1%)
Family member	48.9% (44.1%, 54.2%)
Taxi	2.0% (0.0%, 7.3%)
Walked	0.7% (0.0%, 6.0%)
Other	3.9% (0.0%, 9.2%)
Unknown	3.7% (0.0%, 9.0%)

**Conclusions:** The majority of anaphylaxis cases arrive to the ED within 1 h of symptoms. Differences between the two provinces in arrival time and use of ambulance may be related to differences in catchment population given the larger metropolitan areas served by the Quebec centers.

### A16 Detection of blood biomarkers of the late-phase asthmatic response in sputum

#### Daniel He^1,2^, India Bayly^1,2^, Amrit Singh^1,2^, Roma Sehmi^3^, Gail M. Gauvreau^3^, Scott J. Tebbutt^1,2,4^

##### ^1^Centre for Heart Lung Innovation, University of British Columbia, Vancouver, British Columbia, V6Z1Y6, Canada; ^2^Prevention of Organ Failure (PROOF) Centre of Excellence, Vancouver, British Columbia, V6Z2K5, Canada; ^3^Department of Medicine, McMaster University, Hamilton, Ontario, L8N3Z5, Canada; ^4^Division of Respiratory Medicine, Department of Medicine, University of British Columbia, Vancouver, British Columbia, V5Z1M9, Canada

###### **Correspondence:** Daniel He (daniel.he@hli.ubc.ca)

*Allergy, Asthma & Clinical Immunology* 2019, **15**(**Suppl 2**):A16

**Background:** Allergic asthma is characterized by bronchoconstriction in response to an allergen, resulting in a drop in FEV_1_. Half of allergic asthmatic patients exhibit the early-phase asthmatic response (EAR) occurring ~ 30 min post-exposure, whereas the other half develop a late-phase asthmatic response (LAR) ~ 3–8 h post-exposure in addition to the EAR. Both responses are recapitulated in the allergen inhalation challenge (AIC) model, which is used to assess allergic airway disease. Our group has previously identified a set of blood-based biomarkers of gene expression that can predict whether an asthmatic individual will be an early responder, ER (EAR only), or a dual responder, DR (EAR and LAR), at baseline pre-allergen challenge [1]. In the current study, we aimed to determine whether the genes of our blood biomarker panel could be detected in sputum samples of asthmatic individuals undergoing an AIC.

**Methods:** Mild, atopic asthmatics (n = 5, all DRs) underwent an AIC. Sputum samples were collected at 0 h (pre-challenge) and at 7 and 24 h post-challenge, and sputum sample RNA was stabilized with TRIzol and extracted using the RNeasy Mini Kit (Qiagen). Relative expression levels of 180 genes were measured using a custom nCounter Elements asthma biomarker assay (NanoString Technologies). Data analysis was conducted using R, and a statistical significance threshold of 0.05 was used after correction for multiple hypothesis-testing with Benjamini–Hochberg false discovery rate (FDR).

**Results:** All measured genes in our sputum samples were detectable above the background threshold set by our blood-based assay, indicating that genes in blood that discriminate DRs from ERs at baseline can be detected in sputum. Gene expression analysis revealed that 41 genes were differentially expressed (FDR < 0.05) at 7 h post-challenge compared to baseline, the most significant of which was the upregulation of *CXCL1*, a regulator of mast cell chemotaxis (Table 1). Notably, when comparing gene expression 24 h post-challenge against baseline, we identified 19 differentially expressed genes (FDR < 0.05), 14 of which were also differentially expressed at 7 h post-challenge, suggesting the effects of the AIC persist at the gene expression level at least a day after initial allergen exposure.

**Table 1:** Top 5 differentially expressed genes at 7 h after initial allergen exposure

Gene	Description	Log_2_ fold change	*p* value	FDR
*CXCL1*	Chemokine (C-X-C motif) ligand 1	2.03	1.7 × 10^−7^	1.8 × 10^−5^
*CISH*	cytokine-inducible SH2-containing protein	2.31	2.3 × 10^−7^	1.8 × 10^−5^
*CCR2*	C–C chemokine receptor type 2	1.90	4.4 × 10^−6^	2.3 × 10^−4^
*LILRA1*	leukocyte immunoglobulin-like receptor A1	2.10	9.1 × 10^−6^	3.6 × 10^−4^
*CD300LB*	CD300 molecule like family member B	1.21	1.4 × 10^−5^	4.2 × 10^−4^

**Conclusions:** Predictive blood biomarkers of the LAR can be detected in sputum samples and are differentially expressed upon allergen exposure. Further investigation of sputum samples obtained from individuals exhibiting only the EAR during an AIC may provide valuable insights into the mechanisms driving these phenotypes.

**Reference**

1. Singh A, Shannon CP, Kim YW, Yang CX, Balshaw R, Cohen Freue GV, Gauvreau GM, FitzGerald JM, Boulet LP, O’Byrne PM, Tebbutt SJ. Novel blood-based transcriptional biomarker panels predict the late-phase asthmatic response. Am J Respir Crit Care Med. 2018;197(4):450–62.

### A17 Engaging knowledge users: usability testing of the Canadian Anaphylaxis Emergency Plan

#### Abeer Hegazi^1^, Susan Waserman^1^, Ernie Avilla^1^, Monika Kastner^2^

##### ^1^Division of Clinical Allergy and Immunology, Department of Medicine, McMaster University, Canada; ^2^Knowledge Translation and Implementation, North York General Hospital, Toronto, Canada

###### **Correspondence:** Abeer Hegazi (abeer.hegazi@medportal.ca)

*Allergy, Asthma & Clinical Immunology* 2019, **15**(**Suppl 2**):A17

**Background:** There are no published studies on the usability of Anaphylaxis Emergency Plans (AEP). Most have not had methodologically rigorous usability evaluations to reveal problems and errors, nor have they been tested on relevant knowledge end-users. The objectives of the study were to: (1) conduct a usability evaluation of the Canadian Society of Allergy and Clinical Immunology (CSACI) AEP; and (2) assess whether the AEP helps with the appropriate emergency response to a severe allergic reaction.

**Methods:** *Population:* Study inclusion criteria: (1) adult patients/caregivers of children with known allergies (age ≥ 18); (2) children (age < 18 years) at risk of anaphylaxis who are able to self-administer an EAI; and (3) school staff (i.e. teachers). Teacher recruitment was a convenience sample within one school board. *Setting:* Usability sessions took place at the Simulation Laboratory at McMaster University. *Process:* Participants were asked to demonstrate the use of the AEP using a simulation man in the context of two scenarios: Case A presented with symptoms of headache and itchy rash on the back; and Case B presented with abdominal pain, mouth/tongue swelling, difficulty breathing, and vomiting. The sessions were 60 min, audio- and video-taped and transcribed verbatim. Participants were interviewed using an open-ended questionnaire and Likert-type questions about the format, features, and other qualities of the AEP.

**Results:** Sixteen participants agreed to participate: 5 children, 7 parents, and 4 school staff. For Case A, participants completed the scenario at a MEAN time of 151.7 s with four participants (25%) used EAI unnecessarily. For Case B, participants completed the scenario at a MEAN time of 120.9 s with three participants (19%) failed to identify anaphylaxis.

Overall satisfaction of the AEP was 60–65% with parents being the least satisfied. 78% of participants were able to identify anaphylaxis symptoms. Most participants stated that a bigger font and a simpler sequence of instructions are needed, as well as list symptoms by severity and number of symptoms or systems involved to help with decision making regarding epinephrine use.

Some participants stated that including pictures can facilitate accuracy and timely actions during such a stressful event.

**Conclusion:** Understanding the usability factors that affect end-user knowledge and use of AEP are critical for their development.

Based on this feedback, our ultimate goal is to optimize the current AEP and retest its usability.

**Acknowledgements:** This work was supported by AllerGen NCE Inc. and Food Allergy Canada

### A18 A precision cut intestinal slice model for studies in food allergy

#### Lisa Hung^1,2^, Gary Yu^1^, Helena Obernolte^3^, Yun Hye Kim^1^, Hoon-Ki Sung^1,4^, Eva Untersmayr-Elsenhuber^5^, Katrine Lindholm Bøgh^6^, Alexander Eggel^7^, Katherina Sewald^3^, Iram Siddiqui^8^, Gino Somers^4,8^, Priscilla Chiu^9^, Thomas Eiwegger^1,2,10^

##### ^1^Translational Medicine Program, Research Institute, The Hospital for Sick Children, Toronto, M5G 1X8, Canada; ^2^Department of Immunology, University of Toronto, Toronto, M5S 1A1, Canada; ^3^Department of Preclinical Pharmacology and In-Vitro Toxicology, Fraunhofer ITEM, 30625 Hannover, Germany; ^4^Department of Laboratory Medicine and Pathobiology, University of Toronto, Toronto, M5S 1A1, Canada; ^5^Gastrointestinal Immunology, Medical University of Vienna, 1090 Vienna, Austria; ^6^Research Group for Food Allergy, National Food Institute, Technical University of Denmark, 2800 Kgs Lyngby, Denmark; ^7^Department of Rheumatology, Immunology and Allergology, University Hospital of Bern, 3010 Bern, Switzerland; ^8^Department of Paediatric Laboratory Medicine, The Hospital for Sick Children, Toronto, M5G 1X8, Canada; ^9^Division of Pediatric General and Thoracic Surgery, The Hospital for Sick Children, Toronto, M5G 1X8, Canada; ^10^Division of Immunology and Allergy, Food Allergy and Anaphylaxis Program, The Hospital for Sick Children, Toronto, M5G 1X8, Canada

###### **Correspondence:** Thomas Eiwegger (thomas.eiwegger@sickkids.ca)

*Allergy, Asthma & Clinical Immunology* 2019, **15**(**Suppl 2**):A18

**Background:** There is currently no food allergy model available that can reflect the complexity of gastrointestinal (GI) tissue, including the immune cells and the functionality of the enteric nervous system. Consideration of these aspects is crucial to obtaining an accurate representation of the cellular mechanisms that occur in the gut during an allergic reaction. An ex vivo tissue model that includes these features would be especially useful for the development of new treatments and to further understand the cause and progression of this disorder. Precision cut intestinal slices (PCIS) are mini-models of gut tissue that maintain the intestinal microenvironment, multicellularity and function of the organ. They can be generated from many different animals, including humans, and can therefore represent all areas of the gut across multiple species. Through a passive sensitization strategy, PCIS may be used to test potential therapeutics for food allergy prior to clinical trials. The purpose of this project was to develop a novel PCIS model that allows for the study of acute IgE-mediated allergic reactions in the GI tract of rats, mice and humans.

**Methods:** Gut tissue from rats, mice and human donors were extracted, washed, embedded in agarose and cut into 400 µm thick slices. The resulting PCIS were cultured in an oxygenated incubator and assessed for maintenance of viability over time. PCIS were passively sensitized using serum from allergic individuals from their respective species and stimulated with the relevant allergen at different concentrations or serotonin as a positive control. Allergen specific IgE-crosslinking and subsequent mediator release resulted in visible, quantifiable smooth muscle contractions as a readout.

**Results:** Gut tissue viability from rats, mice and humans was maintained up to 12 h. PCIS generated from non-allergic Brown Norway rats and BALB/c mice passively sensitized with serum from Ara h 2 (peanut) sensitized rats or OVA sensitized mice displayed allergen specific smooth muscle contractions upon peanut extract or OVA stimulation, respectively. This pattern was also observed in human gut tissue sensitized with sera from peanut allergic patients and stimulated with peanut extract.

**Conclusions:** We have developed a novel gut tissue-based model for food allergy that shows acute, allergen specific reactions in three different species. The PCIS model is unique in that it incorporates the complexity and functionality of intestinal tissue. Human PCIS may be used as a pre-clinical model to evaluate the effect of novel therapies on the allergic response or to study mechanisms of desensitization or tolerance development.

### A19 Bacteria and rhinovirus induce interleukin-17C release to promote CXCL1 release and neutrophil chemotaxis

#### Kyla C. Jamieson^1^, Suzanne Traves^1^, Curtis Dumonceaux^1,2^, Richard Leigh^1,2^, David Proud^1^

##### ^1^Department of Physiology & Pharmacology, University of Calgary, Calgary, Canada; ^2^Department of Medicine, University of Calgary, Calgary, Canada

###### **Correspondence:** Kyla C. Jamieson (kyla.jamieson@ucalgary.ca)

*Allergy, Asthma & Clinical Immunology* 2019, **15**(**Suppl 2**):A19

**Background:** Bacterial and viral respiratory infections are common triggers of asthma and chronic obstructive pulmonary disease (COPD) exacerbations. Human rhinovirus (HRV) is the dominant viral pathogen identified, and non-typeable *Haemophilus influenzae* (NTHI) is among the most common bacterial species detected. Asthma and COPD patients who have bacteria-virus co-infections have more severe exacerbations with increased length of hospitalization [1, 2]. The airway epithelium defends against these pathogens with several mechanisms including pro-inflammatory and anti-microbial cytokine release. Interleukin (IL)-17C is produced by epithelial cells in COPD patients and in response to NTHI [3]; however, there are no data assessing how rhinovirus infections affect IL-17C. We hypothesize that the epithelium releases IL-17C in response to HRV + NTHI infections as a protective anti-microbial mechanism, and that this response is disrupted in COPD.

**Methods:** Primary human bronchial epithelial cells (HBECs) isolated from normal human lungs, or HBECs from bronchial brushings of healthy non-smokers, smokers with normal lung function (healthy smokers), and patients with confirmed COPD, were treated with NTHI and/or HRV. HBECs were exposed to IL-17C to assess its functional role. Protein and mRNA levels were measured using ELISA and qRT-PCR, respectively. Neutrophils were isolated from blood of healthy donors and chemotactic properties of HBEC supernatants were assessed using a modified Boyden chamber.

**Results:** Bacteria-HRV co-exposure induces synergistic IL-17C release from HBECs through NFκB and p38 signalling and RIG-I/MDA5 recognition of viral replication products. Microbial-induced IL-17C is attenuated by cigarette smoke extract and in cells from healthy smokers. Conversely, cells from COPD patients have an enhanced microbial-induced IL-17C response compared to non-smokers and healthy smokers. IL-17C induces epithelial release of CXCL1, and supernatants from IL-17C-stimulated HBECs induce neutrophil chemotaxis. Further, IL-17C contributes to NTHI + HRV-induced CXCL1 release and neutrophil chemotaxis.

**Conclusions:** Bacteria-rhinovirus co-infections result in a robust and synergistic IL-17C response from the airway epithelium, which acts to promote CXCL1 release and neutrophil recruitment. Acute and chronic smoke exposure reduce IL-17C release, except in COPD patients. Differential IL-17C responses to NTHI-HRV co-infections suggest that further studies are required to determine if IL-17C is a predictive biomarker of COPD in smokers, or if the response is a consequence of the inflammatory environment.

**Acknowledgements:** This work was supported by the Alberta Lung Association, University of Calgary’s Eyes High, and by the Canadian Institute for Health Research (CIHR).

**References**

1. Wark P, Tooze M, Powell H, Parsons K. Viral and bacterial infection in acute asthma and chronic obstructive pulmonary disease increases risk of readmission. Respirology. 2013;18(6):996–1002.
2. Papi A, Bellettato CM, Braccioni F, Romagnoli M, Casolari P, Garamori G, Fabbri LM, Johnston SL. Infections and airway inflammation in chronic obstructive pulmonary disease severe exacerbations. Am J Respir Crit Care Med. 2006;173(10):1114–21.
3. Pfeifer P, Voss M, Wonnenberg B, Hellberg J, Seiler F, Lepper PM, Bischoff M, Langer F, Schafers HJ, Menger MD, Bals R, Beisswenger C. IL-17C is a mediator of respiratory epithelial innate immune response. Am J Respir Cell Mol Biol. 2013; 48(4):415–21.

### A20 Investigating the diagnostic potential of selected urinary metabolites in asthma and COPD using an integrated LC–MS/MS platform

#### Mona M. Khamis^1^, Hanan Awad^2^, Kevin Allen^1^, Anas El-Aneed^1^, Darryl J. Adamko^3^

##### ^1^College of Pharmacy and Nutrition, University of Saskatchewan, Saskatoon, SK, Canada; ^2^Calgary Laboratory Services, Alberta Health Services, Calgary, AB, Canada; ^3^Department of Pediatrics, College of Medicine, University of Saskatchewan, Saskatoon, SK, Canada

###### **Correspondence:** Mona M. Khamis (mmh882@mail.usask.ca)

*Allergy, Asthma & Clinical Immunology* 2019, **15**(**Suppl 2**):A20

**Background:** The clinical presentation of asthma and chronic obstructive pulmonary disease (COPD) sometimes overlap despite their pathological differences. In some patients, doctors rely on history and therapy trials for diagnosis [1]. There is a need for a better objective test that can be routinely applied in an outpatient clinic. Metabolomics is the study of small molecules created by diseases. Our previous untargeted ^1^H-NMR metabolomics analysis suggested 50 urine metabolites as candidate biomarkers for differentiating between asthma and COPD [2]. Compared to ^1^H-NMR, liquid chromatography-tandem mass spectrometry (LC–MS/MS) is more robust for accurate metabolite quantification [3]. We have spent the last 4 years developing three novel LC–MS/MS methods to verify the usefulness of these biomarkers in the diagnosis of asthma and COPD [4–6].

**Methods:** Based on their structures, the metabolites (n = 40) were divided into 3 subgroups and quantified using FDA- and EMA-validated LC–MS/MS methods [4–8]. Differential isotope labeling was used for groups 1–2, while group 3 contains miscellaneous metabolites and was quantified using stable isotopes [4–6]. Urine samples from asthma (n = 43) and COPD (n = 36) subjects were analyzed for groups 1–3 and normalized to creatinine. Statistical separation of groups was performed [SIMCA, partial least square-discriminant analysis (PLS-DA)]. Additional 75 samples are being analyzed for use as a validation set.

**Results:** A PLSA-DA model was constructed using 10 asthma and 10 COPD patients as training sets (non-blinded samples) (R^2^Q^2^ = 0.923, 0.867). Remaining samples were blinded and analyzed using the model. Blinded asthma samples (23 out of 33) were diagnosed with acceptable accuracy (70%). However, the metabolomic profile of several blinded COPD patients demonstrated a pattern similar to asthma (50% accuracy). We reviewed the clinical data for these patients and found that many did not have a clear diagnosis of COPD but had features of asthma instead, suggesting an issue of misdiagnosis.

**Conclusion:** This project has created robust methods for metabolite quantification. Using these methods, we suggest there is a metabolome differentiating asthma and COPD. Further validation is currently ongoing using additional urine samples and saved clinical data, as the presented sample size is still small in comparison to the number of independent variables (i.e. metabolites). Data generated from 154 patients will be available for discussion in the upcoming AllerGen Poster Competition. The additional samples are expected to increase the statistical power, allowing for better assessment of the model’s accuracy and the identification of the most significant metabolites contributing to it.

**References**

1. Chang J, Mosenifar Z. Differentiating COPD from asthma in clinical practice. J Intensive Care Med. 2007;22:300–9.
2. Adamko DJ, Nair P, Mayers I, Tsuyuki RT, Regush S, Rowe BH. Metabolomic profiling of asthma and chronic obstructive pulmonary disease: a pilot study differentiating diseases. J Allergy Clin Immunol. 2015; 136:571–80.e573.
3. Khamis MM, Adamko DJ, El-Aneed A. Mass spectrometric based approaches in urine metabolomics and biomarker discovery. Mass spectrometry reviews. 2015.
4. Awad H, Allen K, Adamko DJ, El-Aneed A. Detection and quantification of 17 organic acid metabolites excreted in the urine of respiratory illness patients using a novel LC–MS/MS method. In: The 21st International Mass Spectrometry Conference (IMSC); Toronto, ON, Canada. 2016
5. Khamis MM, Adamko DJ, Purves RW, El-Aneed A. Quantitative determination of potential urine biomarkers of respiratory illnesses using new targeted metabolomic approach. Analytica Chimica Acta. 2018.
6. Khamis MM, Adamko DJ, El-Aneed A. Development of a validated LC–MS/MS method for the quantification of 19 endogenous asthma/COPD potential urinary biomarkers. Analytica Chimica Acta. 2017;989:45–58.
7. EMA. European Medicines Aagency, Committee for Medicinal Products for Human Use (CHMP), Guidelines on bioanalytical method validation. 2011.
8. US-FDA. Food and Drug Administration, FDA Guidance for Industry:Bioanalytical Method Validation, DRAFT GUIDANCE. US Department of Health and Human Services,FDA, Center for Drug Evaluation and Research, Rockville, MD, USA. 2018. https://www.fda.gov/downloads/drugs/guidances/ucm070107.pdf.

### A21 Modulation of cAMP levels for potentiation of long-acting beta-agonist and glucocorticoids in human airway epithelial cells

#### Yechan Kim^1^, Vincent Hou^1^, Jenny P. Nguyen^1^, Ryan D. Huff^2^, Markus Heller^3^, Mark D. Inman^1^, Jeremy A. Hirota^1,2,4^

##### ^1^Firestone Institute for Respiratory Health, McMaster University, Hamilton, Canada; ^2^Division of Respiratory Medicine, University of British Columbia, Vancouver, Canada; ^3^Centre for Drug Research and Development, Vancouver, Canada; ^4^Department of Biology, University of Waterloo, Waterloo, Canada

###### **Correspondence:** Yechan Kim (kimy44@mcmaster.ca)

*Allergy, Asthma & Clinical Immunology* 2019, **15**(**Suppl 2**):A21

**Background:** Combination long-acting beta-agonist (LABA) and glucocorticoids (GCS) therapies represent the dominant strategy for controlling lung inflammation in asthma. Pharmacological interventions that increase intracellular cAMP beyond existing combination LABA/GCS are likely to be beneficial for the management of difficult-to-control asthmatics that are hypo-responsive to mainstay therapy [1]. PDE4 inhibition is one method of increasing intracellular cAMP levels by preventing the breakdown of cAMP. We have also demonstrated that human airway epithelial cells (HAECs) express ATP Binding Cassette Transporter C4 (ABCC4), which functions to transport cAMP from the cytosol to the extracellular compartment [2]. We aimed to determine if ABCC4 and PDE4 inhibition can potentiate LABA/GCS anti-inflammatory responses in HAECs in a cAMP-dependent mechanism validating the pursuit of cAMP-elevating agents for asthma.

**Methods:** To determine baseline inflammatory responses, HAECs were exposed to increasing concentrations of inflammatory stimuli (TNF-α, IL-1β, and Poly I:C) and ELISAs were performed for GM-CSF, IL-6, and IL-8. Using select inflammatory stimuli concentrations, HAECs were pre-treated with fivefold concentrations of LABA (Formoterol) and fixed GCS (Budesonide) concentration, and vice versa, followed by downstream cytokine analysis by ELISA. With the effect of LABA/GCS therapy on inflammatory stimuli established, we performed studies with ABCC4 inhibitors (MK-571, Ceefourin-1, and CDRD14), PDE4 inhibitors (Roflumilast, Rolipram, and Cilomilast), and combination ABCC4 ± PDE4 inhibitors (Ceefourin-1/CDRD14 and Roflumilast) in the presence of LABA/GCS and inflammatory stimuli.

**Results:** The addition of ABCC4 inhibitors to LABA/GCS showed no further attenuation of cytokine release in all 3 stimuli and 3 cytokines when compared to LABA/GCS alone. The addition of PDE4 inhibitors to LABA/GSCS showed further attenuation for Rolipram (p < 0.0001) and Roflumilast (p < 0.0001) for Poly I:C induced GM-CSF. The addition of combination ABCC4 + PDE4 inhibitors to LABA/GCS showed further attenuation for Roflumilast + Ceefourin-1 for Poly I:C induced IL-6 (p ≤ 0.01) and IL-8 (p < 0.01). In addition, IL-1β induced IL-8 showed a further attenuation in release in both Roflumilast + Ceefourin-1 (p < 0.0001) and Roflumilast + CDRD14 (p < 0.0001).

**Conclusions:** We demonstrated that intracellular cAMP-elevating agents in combination with LABA/GCS can potentiate the attenuation of inflammatory cytokine release in HAECs. Our findings complement our recent demonstration that ABCC4 inhibition potentiates LABA/GCS anti-inflammatory responses by enhancing the upregulation of anti-inflammatory genes and further attenuating cytokine production in response to proinflammatory stimuli. Our results form the basis for a drug development program aimed at developing novel interventions that may be an effective add-on therapy for the treatment of asthma.

**References**

1. Giembycz MA, Newton R. Potential mechanisms to explain how LABAs and PDE4 inhibitors enhance the clinical efficacy of glucocorticoids in inflammatory lung diseases. F1000Prime Rep. 2015;7. 10.12703/p7-16.
2. Huff RD, Rider CF, Yan D, Newton R, Giembycz MA, Carlsten C, et al. Inhibition of ABCC4 potentiates combination beta agonist and glucocorticoid responses in human airway epithelial cells. J Allergy Clin Immunol. 2017.

### A22 Early lung function changes associated with wheezing phenotypes in the CHILD Study

#### Zihang Lu^1,2,#^, Christopher J. Olesovsky^1,3,#^, Melanie Emmerson^1^, Krzysztof Kowalik^1^, Meghan B. Azad^4,5^, Aimée Dubeau^1^, Per Gustafsson^6^, Diana L. Lefebvre^7^, Stuart E. Turvey^8^, Allan B. Becker^4^, Piush J. Mandhane^9^, Felix Ratjen^1^, Wendy Lou^2^, Theo J. Moraes^1^, Malcolm R. Sears^7^, Padmaja Subbarao^1^

##### ^1^Department of Pediatrics, and Translational Medicine, SickKids Research Institute, The Hospital for Sick Children, Toronto, Ontario, Canada; ^2^Dalla Lana School of Public Health, University of Toronto, Toronto, Canada; ^3^Faculty of Medicine, University of Toronto, Toronto, Ontario, Canada; ^4^Department of Pediatrics and Child Health, University of Manitoba, Winnipeg, MB, Canada; ^5^Children’s Hospital Research Institute of Manitoba, Winnipeg, MB, Canada; ^6^Department of Pediatrics, Central Hospital, Skövde, Sweden; ^7^Department of Medicine, Faculty of Health Sciences, McMaster University, Hamilton, Ontario, Canada; ^8^Department of Pediatrics, Child & Family Research Institute, BC Children’s Hospital, University of British Columbia, Vancouver, BC, Canada; ^9^Department of Pediatrics, University of Alberta, Edmonton, Alberta, Canada

###### **Correspondence:** Padmaja Subbarao (padmaja.subbarao@sickkids.ca)

^#^These authors contributed equally to the work

*Allergy, Asthma & Clinical Immunology* 2019, **15**(**Suppl 2**):A22

**Background:** The trajectory of lung function in infancy prior to development of asthma is controversial. Methodologies applicable to any age are helpful in tracking early life lung function changes associated with wheezing disorders. This study aims to determine trajectories of lung function associated with early life wheezing phenotypes.

**Methods:** A sub-cohort of Toronto participants in the Canadian Healthy Infant Longitudinal Development (CHILD) Study underwent repeated pulmonary function testing from infancy using multiple breath washout and forced expiratory maneuvers methodologies. Three wheezing phenotypes were defined by symptoms over the first 3 years of life: transient wheezers (symptoms in the first 24 months only), recurrent wheezers (≥ 2 episodes by 3 years) and non-wheezers.

**Results:** Lung function was tested in infancy and 3 years in 43 transient wheezers, 42 recurrent wheezers and 95 non-wheezers. In infancy, lung clearance index (LCI) was significantly worse in transient compared to recurrent wheezers (mean difference (MD) = 0.47; 95% CI 0.07 to 0.86; p = 0.02) and non-wheezers (MD = 0.54; 95% CI 0.19 to 0.88; p = 0.002). These differences fully resolved by age three. At 3 years of age, LCI was significantly worse in recurrent wheezers compared to non-wheezers (MD: 0.52; 95% CI 0.25 to 0.80; p < 0.001) and transient wheezers (MD = 0.39; 95% CI 0.06 to 0.7; p = 0.02). Spirometry data provided complementary information. During infancy, recurrent wheezers had similar FEV_t_/FVC (FEV_0.5_/FVC for infancy and FEV_0.75_/FVC for preschool-age) z-scores to non-wheezers, whereas transient wheezers had significantly lower FEV_t_/FVC z-scores compared to non-wheezers (MD = − 0.76; 95% CI − 1.42 to − 0.11; p = 0.02). However, by 3 years of age recurrent wheezers were significantly lower than non-wheezers (MD = − 0.53; 95% CI − 0.93 to − 0.14, p = 0.01), whereas there were no significant differences between transient wheezers and non-wheezers.

**Conclusions:** The lung function of recurrent wheezers was similar to non-wheezers in the first year of life but the trajectory significantly differed with lower function at age three.

**Acknowledgements:** This work was supported by the following funding institutions: CIHR, AllerGen NCE, Don & Debbie Morrison, SickKids Foundation.

### A23 Di-butyl phthalate (DBP) worsens allergen-induced lung function decline and alters lower airway innate immunology in crossover human study

#### Danay Maestre-Batlle^1^, Ryan D. Huff^1^, Carley Schwartz^1^, Anette K. Bølling^2,#^, Christopher Carlsten^1,#^

##### ^1^Respiratory Medicine, University of British Columbia, Vancouver, British Columbia, Canada; ^2^Norwegian Institute of Public Health, Oslo, Norway

###### **Correspondence:** Christopher Carlsten (christopher.carlsten@ubc.ca)

^#^Equal contributors

*Allergy, Asthma & Clinical Immunology* 2019, **15**(**Suppl 2**):A23

**Background:** Phthalates are plastic softeners used in commercial products that leak from their carrier plastic and become widespread environmental contaminants [1]. Epidemiological studies suggest an association between exposure to phthalates and respiratory diseases like asthma [2]. DBP, a phthalate found in high concentrations in indoor air, has shown toxicity and inflammatory potential in vitro [3]. We hypothesize that DBP inhalation prior to allergen inhalation will affect lung function as well as recruitment and activation of immune cells in the lower airway.

**Methods:** A controlled, double-blinded, crossover study enrolled 16 allergen-sensitized participants. Recruitment was balanced for airway hyper-responsiveness status (AHR = methacholine provocative concentration causing a 20% fall in FEV_1_ (PC_20_) ≤ 16 mg/ml), allergen inhaled (house dust mite (HDM), birch or grass,) and exposure order. Participants were exposed to control air or DBP (120 µg/m^3^) for 3 h, immediately followed by an inhaled allergen challenge. The area under the curve (AUC), measured as FEV_1_% drop from baseline (3 h post-allergen challenge, versus 0 h) was calculated. A methacholine test was performed the following day. Pulmonary function tests and sample collection (blood, nasal and urine) occurred at several time-points, spanning from before and up to 20 h post-exposure. Bronchoalveolar lavage was acquired 24 h post-exposure. Data were analyzed using a linear mixed effects model.

**Results:** Exposure to DBP augmented the inhaled allergen response, as the AUC increased with DBP compared to control air (p = 0.032, Fig. 1). Non-AHR participants were the main drivers of the DBP effect on the AUC (p = 0.012) and other pulmonary function endpoints. For example, DBP exposure decreased next-day methacholine PC_20_ (p = 0.057) and FEV_1_ 20 h post-exposure (p = 0.018) in non-AHR participants, but not in the full dataset. The type of allergen inhaled after DBP exposure also influenced the AUC effect (HDM p = 0.004, birch p = 0.10, grass p = 0.91) although there was no significant interaction effect. Moreover, DBP exposure increased the recruitment of total macrophages (p = 0.05) and their expression of CD206 (p = 0.05), a pattern recognition receptor that aids phagocytosis and antigen presentation. Within the total macrophage population, the presence of M2 increased (p = 0.09) and M1 decreased (p = 0.08) with DBP exposure. The number of Th1 cells was reduced (p = 0.07) and expression of CD4 on T helpers increased (p = 0.07). AHR did not modify the cellular lower airway response but the type of allergen did.

**Fig. 1:**
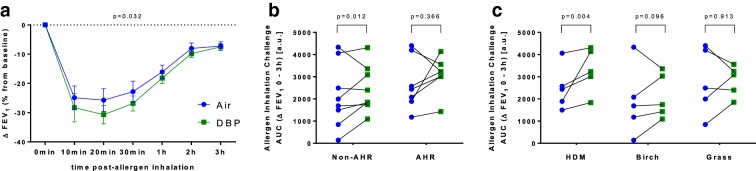
Effect of DBP in response to an inhaled allergen challenge. Linear mixed effects model analysis showing **a** overall DBP effect (comparing AUCs*), **b** role of airway hyper-responsiveness and** c** role of specific allergen. *The AUCs reflects the area between each curve and the dotted line

**Conclusions:** DBP exposure affects allergen-induced lung function, as well as recruitment and activation of lower airway immune cells

**References**

1. Schettler T, Skakkebæk NE, De Kretser D, et al. Human exposure to phthalates via consumer products. Int J Androl. 2006;29:134–9.
2. Deutschie T, Reiter R, Butte W, et al. A controlled challenge study on Di(2-ethylhexyl) phthalate (DEHP) in house dust and the immune response in human nasal mucosa of allergic subjects. Environ Health Perspect. 2008;116:1487–93.
3. Li L, Li HS, Song NN, et al. The immunotoxicity of dibutyl phthalate on the macrophages in mice. Immunopharmacol Immunotoxicol. 2013;35:272–81.

### A24 Overall risk analysis and probability assessment on the presence of allergen traces of milk in dark chocolate in Canada

#### Emilie Manny^1,2^, Sébastien La Vieille^1^, Samuel Godefroy^1,2^

##### ^1^Faculty of Food and Agricultural Sciences, *Université Laval*, Quebec, Quebec, Canada; ^2^Institute of Nutrition and Functional Foods (INAF), *Université Laval*, Quebec, Quebec, Canada

###### **Correspondence:** Emilie Manny (emilie.manny.1@ulaval.ca)

*Allergy, Asthma & Clinical Immunology* 2019, **15**(**Suppl 2**):A24

**Background:** The occurrence of precautionary allergen labeling (PAL) of foodstuffs sold in Canada is increasing [1], reducing the food on offer to allergic consumers [2]. In previous studies, only few surveyed products contained detectable traces of the allergens mentioned in PALs [3]. Also, it was shown that some allergic consumers would adopt risky behavior and eat foodstuffs without paying attention to PALs, potentially resulting in adverse reactions. However, additional studies have shown that the occurrence of milk traces in dark chocolate was higher than other allergen occurrences in other types of food products [4]. The aim of this Canadian study is to undertake a risk assessment simulation based on the presence of milk in dark chocolate with PAL concerning milk.

**Methods:** Dark chocolate products with PAL were analysed for the possible presence of milk. ELISA kits from r-biopharm (RIDASCREEN^®^FAST Milk) were used for the detection and quantification of milk proteins in dark chocolate. The kit was validated prior to use to ensure milk detection before and after processing.

The allergic risk associated with the consumption of milk traces in dark chocolate products will be estimated using the “consumption estimates per eating occasion” data held in the Canadian Community Health Survey (CCHS-2015). The occurrence of adventitious allergens in foods and the dose–response relationship will be estimated with a deterministic and probabilistic approach similar to those already published in the literature [5].

**Results:** So far, 52 dark chocolate products with PAL have been investigated for their milk content and 47 (90.4%) were positive. The range of milk protein content spans from 2.5 to 6231 mg/kg (ppm). The mean of all suggested servings (about 40 g) will be simulated and compared with the mean consumption of dark chocolate in the CCHS-2015 survey. Monte Carlo Simulations on the possibility of getting an allergic reaction will be run using @Risk and SAS software with this acquired data and the known prevalence of milk allergy in Canada.

**Conclusion:** More data must be acquired to undertake risk assessments on the probability of occurrence of allergic reactions occurring in Canadians consuming products with PALs. Based on the results of this study, and of studies on other allergens and occurrence data, guidelines on allergen management practices for the food industry will be proposed.

**Acknowledgements:** This research is financially supported by AllerGen. The authors are thankful to the Canadian Food Inspection Agency for providing guidance and training, and r-biopharm and Morinaga for their support.

**References**

1. Robertson ON, Hourihane JO, Remington BC, Baumert JL, Taylor SL. Survey of peanut levels in selected Irish food products bearing peanut allergen advisory labels. Food Addit Contam Part A. 2013;30(9):1467–72.
2. Hefle SL, Furlong TJ, Niemann L, Lemon-Mule H, Sicherer S, Taylor SL. Consumer attitudes and risks associated with packaged foods having advisory labeling regarding the presence of peanuts. J Allergy Clin Immunol. 2007;120(1):171–6.
3. Ford LS, Taylor SL, Pacenza R, Niemann LM, Lambrecht DM, Sicherer SH. Food allergen advisory labeling and product contamination with egg, milk, and peanut. J Allergy Clin Immunol. 2010;126(2):384–5.
4. Crotty MP, Taylor SL. Risks associated with foods having advisory milk labeling. J Allergy Clin Immunol. 2010;125(4):935–7.
5. Rimbaud L, Heraud F, La Vieille S, Leblanc C, Crepet A. Quantitative risk assessment relating to adventitious presence of allergens in food: a probabilistic model applied to peanut in chocolate. Risk Anal. 2010;30(1):7–19.

### A25 Infant sleep duration is associated with gut microbiota composition in the Canadian Healthy Infant Longitudinal Development (CHILD) study

#### Brittany A. Matenchuk^1^, Theodore Konya^2^, Sukhpreet K. Tamana^1^, Wendy Y.W. Lou^2^, Diana L. Lefebvre^3^, Malcolm R. Sears^3^, Allan B. Becker^4^, Meghan B. Azad^4^, Theo J. Moraes^5^, Stuart E. Turvey^6^, Padmaja Subbarao^5^, James A. Scott^2^, Anita L. Kozyrskyj^1^, Piush J. Mandhane^1^

##### ^1^Department of Pediatrics, University of Alberta, Edmonton, AB, Canada; ^2^Dalla Lana School of Public Health, University of Toronto, Toronto, Canada; ^3^Department of Medicine, McMaster University, Hamilton, Canada; ^4^Department of Pediatrics & Child Health, Children’s Hospital Research Institute of Manitoba, University of Manitoba, Winnipeg, MB, Canada; ^5^Department of Pediatrics, Hospital for Sick Children, University of Toronto, Toronto, ON, Canada; ^6^Department of Pediatrics, Child & Family Research Institute, BC Children’s Hospital, University of British Columbia, Vancouver, BC, Canada

###### **Correspondence:** Anita L. Kozyrskyj (kozyrsky@ualberta.ca), Piush J. Mandhane (mandhane@ualberta.ca)

*Allergy, Asthma & Clinical Immunology* 2019, **15**(**Suppl 2**):A25

**Background:** Sleep and the gut microbiome are intimately linked—as little as a few days of sleep deprivation leads to overgrowth of the intestinal microbiome. In infancy, both sleep and the gut microbiota play an integral role in the development of the immune system and subsequent health throughout life. The objective of this study was to investigate the impact of sleep duration on infant gut microbiota composition while controlling for factors known to influence the infant gut microbiota.

**Methods:** A substudy was conducted on 437 infants with sleep and microbiome data whose mothers were enrolled at the Edmonton site of the CHILD birth cohort. Infant sleep duration at 3 months was assessed using the Brief Infant Sleep Questionnaire (BISQ). Infant gut microbiota were profiled using 16S rRNA sequencing from faecal samples collected at 3 months of age. Birth records and maternal report were the source of the covariate measures such as birth mode and breastfeeding status. Nonparametric statistical testing and logarithmic regression modelling was used to examine the relationship between sleep and abundance of gut microbial taxa. MaAsLin was utilized to determine which microbial taxa were associated with shorter infant sleep while adjusting for covariates. Linear regression was conducted on arcsine square root transformed gut bacterial relative abundance.

**Results:** Sixty-one percent of infants met the National Sleep Foundation recommendation of ≥ 14 h. Sleep duration (as a continuous measure) was negatively associated with the relative abundance of *Clostridium* (Spearman Rho: − 0.10, p = 0.03). Short sleepers (< 14 h per 24-h period) were more likely to be colonized with *Enterococcus* (76.5% vs. 65.2%, p = 0.01; *Exact*) and *Clostridium* (80.6% vs. 67.4%, p < 0.01) and less likely to be colonized with *Erwinia* (5.9% vs. 12.7%, p = 0.02) than infants obtaining ≥ 14 h of sleep. Additionally, arcsine square root transformed *Lachnospiraceae* was positively associated with continuous sleep duration in exclusively breastfed (β: − 0.07, 95% CI − 0.12; − 0.02;p = 0.01) and in infants born vaginally without exposure to maternal antibiotics (β: − 0.07, 95% Confidence Interval [CI]: − 0.13; − 0.01;p = 0.02) following adjustment. *Erwinia* was the only bacterial taxa identified by MaAsLin to be associated with short sleep following adjustment for metadata (beta-coefficient: − 0.00065, p = 0.016).

**Conclusions:** We found the relative abundances of gut bacteria which are associated with infant weight gain, namely *Lachnospiraceae* in breastfed and vaginally born infants without IAP, and *Enterobacteriaceae* in infants born by emergency CS, to be positively associated with infant sleep duration at 3 months of age. These bacterial taxa have also been associated with inflammation and may be a marker of increased inflammation in infants who sleep longer at this time point.

**Acknowledgements:** The authors would like to acknowledge the CHILD study participants and Investigators, without whom this research would not be possible.

### A26 Modernization of birth: impact on *Clostridioides difficile (C. difficile*) colonization in the gut microbiota of Canadian infants at 3 months of age

#### Cara A. McLean^1^, Radha S. Chari^1^, Bonita Lee^2^, Nadia M Lizcano,^3^ Meghan B. Azad^4^, Allan B. Becker^4^, Piushkumar J. Mandhane^2^, Malcolm R. Sears^5^, Stuart E. Turvey^6^, Theo J. Moraes^7^, Padmaja Subbarao^7^, James A. Scott^3^, Anita L. Kozyrskyj^1,2,8^

##### ^1^Department of Obstetrics & Gynecology, University of Alberta, Edmonton, Alberta, Canada; ^2^Department of Pediatrics, University of Alberta, Edmonton, Alberta, Canada; ^3^Dalla Lana School of Public Health, University of Toronto, Toronto, Ontario, Canada; ^4^Department of Pediatrics and Child Health, University of Manitoba, Winnipeg, Manitoba, Canada; ^5^Department of Medicine, McMaster University, Hamilton, Ontario, Canada; ^6^Department of Pediatrics, University of British Columbia, Vancouver, British Columbia, Canada; ^7^Department of Pediatrics, University of Toronto, Toronto, Ontario, Canada; ^8^School of Public Health, University of Alberta, Edmonton, Alberta, Canada

###### **Correspondence:** Cara A. McLean (mclean3@ualberta.ca)

*Allergy, Asthma & Clinical Immunology* 2019, **15**(**Suppl 2**):A26

**Background:** Medical interventions during childbirth are increasing, with cesarean section (CS) delivery exceeding recommended rates by 13% in Canada. CS has been associated with gut dysbiosis in early life. Infants who bypass the beneficial maternal bacterial inoculation provided during vaginal birth have been found to be commonly colonized by opportunistic bacteria such as *C. difficile*, but factors leading to colonization remain unknown. This study aimed to determine the impact of labour interventions on the colonization of *C. difficile* at 3 months of age.

**Methods:** This was a prospective cohort study utilizing data on 1477 mother–infant pairs from the Canadian Healthy Infant Longitudinal Development (CHILD) population-based birth cohort. Labour interventions (i.e., caesarean delivery, anesthetics and drugs to stimulate labor such as oxytocin, carbetocin, prostaglandins), and maternal and infant covariates were collected from hospital charts or maternal questionnaires. *C. difficile* was detected in infant fecal samples collected at 3–4 months of age using quantitative polymerase chain reaction and classified as present/absent. Logistic regression models were run to determine whether labour interventions were associated with *C. difficile* colonization, and adjusted for covariates.

**Results:** Almost one-third of infants were colonized with *C. difficile* at 3 months of age. This varied by birth method; *C. difficile* rates were 28%, 31%, 41% and 38% in vaginal birth with maternal intrapartum antibiotic prophylaxis (IAP), vaginal birth no IAP, emergency CS and elective CS, respectively. In unadjusted analysis, the risk of colonization with *C. difficile* was significantly increased with emergency CS and elective CS compared to vaginal birth with no IAP (OR 1.76, 95% CI 1.27–2.44 p = 0.001 and OR 1.55, 95% CI 1.06–2.26 p = 0.024, respectively). Following adjustment for maternal gravida status, birthweight, anaesthetic and oxytocin use during delivery, hospital length-of-stay, maternal ethnicity and age, prenatal depression, postnatal smoking and breastfeeding, the association remained significant for infants born by emergency CS (aOR 1.72, 95% CI 1.15–2.55 p = 0.007). Oxytocin-like drugs and anesthetics were used in 47% and 77% of all births, respectively. After stratification for these drugs, the increased risk of *C. difficile* in infants born by emergency CS compared to vaginal birth with no IAP remained significant only for infants whose mothers received anesthetics and oxytocin-like drugs during delivery (aOR 1.85, 95% CI 1.21–2.83 p = 0.004).

**Conclusions:** Emergency cesarean delivery was significantly associated with *C. difficile* colonization during infancy and this did not appear to be related to labour induction or anaesthesia. Colonization with this bacterium has been linked to the development of atopic disease.

### A27 Airway anti-MARCO antibodies impair macrophage bacterial efferocytosis in severe eosinophilic asthma

#### Manali Mukherjee^1^, Joseph Chon^2^, Mark Sorin^1^, Katherine Radford^1^, Terence Ho^1^, Paige Lacy^3^, Dawn M.E. Bowdish^2^, Parameswaran K. Nair^1^

##### ^1^St. Joseph’s Healthcare and McMaster University, Department of Medicine, Hamilton, Ontario, Canada; ^2^Pathology and Molecular Medicine, McMaster Immunology Research Centre, McMaster University, Department of Medicine, Ontario, Canada; ^3^Alberta Respiratory Centre, University of Alberta, Edmonton, Alberta, Canada

###### **Correspondence:** Manali Mukherjee (mukherj@mcmaster.ca)

*Allergy, Asthma & Clinical Immunology* 2019, **15**(**Suppl 2**):A27

**Background:** Increased levels of sputum autoantibodies (aAbs) were reported in the airways of severe eosinophilic asthmatics with recurrent infective bronchitis [1]. We hypothesized that autoantibody-mediated macrophage (Mφ) dysfunction could contribute to impaired host defense.

**Methods:** Sputum anti-eosinophil peroxidase IgG levels were used as a marker of airway autoimmunity. Immunoprecipitated immunoglobulins (IP-Igs) from sputa with high (n = 4), low/no (n = 4) aAb titers and healthy (n = 3) were incubated with peripheral blood monocytes for 24 h, and assessed for cytokine release (Eve Technologies, Alberta). Monocyte-derived Mφs (MDMs) were primed with IP-Igs (5 µg/5 × 10^5^ Mφ) for 30 min at 37 °C and further incubated with *S. pneumoniae P1547* to test their phagocytic ability as per standard protocol of measuring colony-forming units [2]. Finally, Ip-Ig reactivity against non-permeabilized Mφs was tested using immunofluorescence (IF), and macrophage receptor with collagenous structure (MARCO) was assessed as one of the possible target antigen (ELISA).

**Results:** Sputum with high aAb titers allowed significant release of pro-inflammatory cytokines from monocytes viz, tumour-necrosis factor alpha, IL-6, IL-1ß and granulocyte macrophage-colony stimulating factor (P < 0.05). As evident in Fig. 1, there was an increased detection of anti-MARCO (IgG) titers up to 1:8 titer in the sputa of eosinophilic asthmatics with recurrent infections (n = 19) compared to those without (n = 23) (P < 0.001), further confirmed by IF. Compared to untreated, MDMs primed with sputum IP-Igs with detectable (n = 6) and low/nil anti-MARCO IgGs (n = 4) compromised bacterial uptake by 39 ± 15% and 10 ± 7% respectively (P = 0.004). There was no effect on innate killing capacity.

**Fig. 1:**
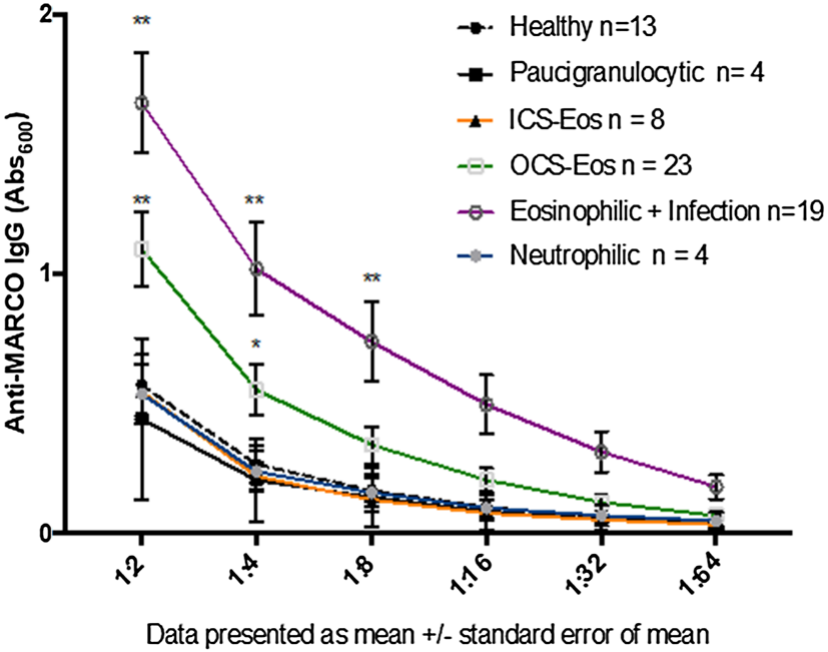
Sputum anti-MARCO IgG titers in different sub-groups of asthmatics stratified based on airway inflammation. **Indicates significant difference. Two ANOVA with Tukey’s multiple correction

**Conclusions:** We report that presence of sputum aAbs against Mφ proteins, in particular scavenger receptors, could impede effective host defense and lead to recurrent infective bronchitis in eosinophilic asthmatics.

**Acknowledgements:** This work is supported by Canadian Institutes of Health Research (CIHR). Presenting author MM is supported by postdoctoral fellowships from CIHR and Canadian Asthma Allergy and Immunology Foundation/AllerGen NCE.

**Refeences**

1. Mukherjee M, Bulir DC, Radford K, Kjarsgaard M, Huang CM, Jacobsen EA, Ochkur SI, Catuneanu A, Lamothe-Kipnes H, Mahony J et al. Sputum autoantibodies in patients with severe eosinophilic asthma. J Allergy Clin Immunol. 2018;141(4):1269–79.
2. Novakowski KE, Loukov D, Chawla V, Bowdish DME. Bacterial binding, phagocytosis, and killing: measurements using colony forming units. In: R. Botelho, editor. Phagocytosis and phagosomes: methods and protocols. New York: Springer New York. 2017. p. 297–309.

### A28 Inexpensive air quality monitoring for the evaluation of urban infrastructure interventions

#### Peter Murphy^1^, Carson Clark^1^, Cheol-Heon Jeong^1^, Jeffrey Brook^1^, David Kuperman^2^, Greg Evans^1^

##### ^1^SOCAAR (Southern Ontario Center for Atmospheric Aerosol Research)–University of Toronto, Toronto, Ontario, M5T 3A1, Canada; ^2^City of Toronto, Toronto, Ontario, Canada

###### **Correspondence:** Peter Murphy (pete.murphy@mail.utoronto.ca)

*Allergy, Asthma & Clinical Immunology* 2019, **15**(**Suppl 2**):A28

**Background:** The King Street Pilot intervention was implemented November 11, 2017, in Toronto, Canada. This City of Toronto traffic policy restricted vehicles from passing through intersections in one of Toronto’s busiest traffic corridors to improve movement on King Street. We aim to determine the efficacy of our inexpensive air quality monitor, AirSENCE, in the evaluation of air impact of the King Street intervention.

**Methods:** AirSENCE devices were deployed 9 days before the beginning of the King Street Pilot intervention on the north and south ends of Metro Hall Toronto (140 m apart), 3 m above and 3 m from the road. Two units were also deployed to street cars for mobile sampling, allowing us to study both King and Wellington Streets. AirSENCE records voltages of multiple inexpensive sensors at 2 min resolution, and wirelessly transmits to a database at the University of Toronto.

By running the two devices alongside Ministry of the Environment reference monitors, October 2017 and February 2018, a linear regression was formed to calibrate raw voltage values to pollutant concentrations. The following pollutants (R squared to reference) were measured; carbon monoxide (0.91), nitrogen monoxide (0.95), and pm2.5 (0.98). Diurnal, weekly, and monthly trends, and comparisons between peak hours were developed.

**Results:** The AirSENCE devices revealed temporal and spatially related differences. Variations in the concentrations of some pollutants on King Street were seen over the duration of the study. For example, the concentration of nitric oxide on King Street was found to decrease by 36% between December and June. Spatial differences were also detected. For example, carbon monoxide was found to have a 48% increase during afternoon peak hours on Wellington, which was not reflected on the King Street Intervention site.

**Conclusions:** The major throughway King Street has lower afternoon rush hour peaks after the implementation of the King Street Pilot intervention when compared to the parallel Wellington Street.

**Acknowledgements:** This work is a collaboration between SOCAAR (Southern Ontario Center for Atmospheric Aerosol Research) and AUG Signals. This work was supported by AllerGen NCE Inc. (the Allergy, Genes and Environment Network), a member of the Networks of Centres of Excellence Canada program.

### A29 Assessing early life exposure to phthalates in the CHILD Study

#### Garthika Navaranjan^1^, Huan Shu^2,3^, Tim K. Takaro^4^, Amanda J. Wheeler^5,6^, Meghan B. Azad^7^, Allan B. Becker^7^, Ruixue Dai^8^, Miriam L. Diamond^1^, Shelley A. Harris^1,9^, Diana L. Lefebvre^10^, Zihang Lu^8^, Piush J. Mandhane^11^, Kathleen McLean^12^, Theo J. Moraes^1,8^, James A. Scott^1^, Padmaja Subbarao^1,8^, Stuart E. Turvey^13^, Malcolm R. Sears^10^, Jeffrey R. Brook^1,14^

##### ^1^University of Toronto, Toronto, ON, Canada; ^2^Stockholm University, Stockholm, Sweden; ^3^Karlstad University, Karlstad, Sweden; ^4^Simon Fraser University, Burnaby, BC, Canada; ^5^Australian Catholic University, Melbourne, VIC, Australia; ^6^University of Tasmania, Hobart, TAS, Australia; ^7^University of Manitoba, Winnipeg, MB, Canada; ^8^Hospital for Sick Children, Toronto, ON, Canada; ^9^Cancer Care Ontario, Toronto, ON, Canada; ^10^McMaster University, Hamilton, ON, Canada; ^11^University of Alberta, Edmonton, AB, Canada; ^12^BC Centre for Disease Control, BC, Canada; ^13^University of British Columbia, Vancouver, BC, Canada; ^14^Environment Canada, Toronto, ON, Canada

###### **Correspondence:** Garthika Navaranjan (Garthika.navaranjan@mail.utoronto.ca)

*Allergy, Asthma & Clinical Immunology* 2019, **15**(**Suppl 2**):A29

**Background:** Phthalates, used as plasticizers, are ubiquitous in the indoor environment, and because of their endocrine-disrupting properties have been banned or restricted in products such as children’s toys. Epidemiological studies have yielded inconsistent associations between phthalate exposure and the development of childhood asthma, possibly related to the exposure window, as few studies have examined exposure during infancy. The Canadian Healthy Infant Longitudinal Development (CHILD) Study provides the opportunity to study phthalate effects on asthma more closely. Our goal was to characterize exposure to phthalates during infancy and early childhood, utilizing CHILD samples and questionnaire data, to inform future epidemiological analyses.

**Methods:** Detailed questionnaires were serially administered documenting the home environment and maternal and infant behaviours. House dust, a known reservoir for phthalates released from products, was collected at 3 months in the most used living room and child’s bedroom. Chemical analyses of dust for six phthalates in 120 homes have been completed. CHILD urine samples collected at ages 3, 12 and 36 months from the first 1578 children have been analyzed for eight phthalate metabolites, reflecting different types of phthalates used in products common in homes. Geometric mean (GM) concentrations for each phthalate metabolite were calculated at each age. Trends with age and factors that may influence urinary phthalate concentrations were examined using mixed models. Associations between phthalate metabolite concentrations in urine, reflecting short-term exposure, and phthalates in house dust (when available), reflecting longer term exposure, were assessed using Spearman correlation.

**Results:** Urine results indicated widespread exposure to phthalates during early life. The highest urinary metabolite concentration was for MBP at all ages (GM: 15–32 ng/mL). Concentrations of all phthalate metabolites significantly increased from 3 to 36 months. Statistically significant differences in concentrations were observed for all phthalate metabolites by household income (higher concentrations in the lowest income category). Significant positive correlations were observed between BzBP in dust from either room and the corresponding metabolite (MBzP) in urine at all time points (*ρ *= 0.28–0.55, p < 0.05).

**Conclusions:** Children have a range of exposure to phthalates during early life. Urinary phthalate metabolite concentrations were highest in low income homes, and concentrations of BBzP in house dust were correlated with its urinary metabolite at all ages. Future work will characterize phthalate exposure from house dust in an additional 750 CHILD subjects as a measure of longer-term exposure in a case-cohort study assessing the association of phthalates with development of childhood asthma, including gene-environment interactions.

### A30 An overview of silicone rubber passive air sampler for phthalates and flame retardants

#### Joseph O. Okeme^1^, Congqiao Yang^2^, Atousa Abdollahi^2,3^, Linh V. Nguyen^1^, Maria Lorenzo Martinez^4^, Suman Dhal^2^, Liisa M. Jantunen^3,2^, J. Mark Parnis^5^, Miriam L. Diamond^1,2^

##### ^1^University of Toronto Scarborough, Department of Physical and Environmental Science, 1265 Military Trail Toronto, ON, M1C 1A4, Canada; ^2^Department of Earth Sciences, 22 Russell Street, University of Toronto, Toronto, ON, M5S 3B1, Canada; ^3^Air Quality Processes Research Section, Environment and Climate Change Canada, 6248 Eighth Line Egbert, ON, L0L 1N0, Canada; ^4^Food and Environmental Safety Research Group (SAMA-UV), Faculty of Pharmacy, University of Valencia, Av. Vicente Andrés, s/n 46100 Burjassot, Valencia Spain; ^5^Chemical Properties Research Group, Department of Chemistry, Trent University, Peterborough, Ontario, Canada

###### **Correspondence:** Joseph O. Okeme (joe.okeme@mail.utoronto.ca)

*Allergy, Asthma & Clinical Immunology* 2019, **15**(**Suppl 2**):A30

**Background:** Exposure to phthalates and other semi-volatile organic compounds (SVOCs) has been associated with adverse health concerns including asthma. To assess exposure in large scale studies, we need more efficient, easily used and inexpensive methods compared to traditional approaches such as active air sampling. Here, we provide an overview of how we have advanced the use of commonly available silicone rubber or polydimethylsiloxane (PDMS) and other passive air samplers (PAS) for improving estimates of exposure to phthalates and other SVOCs in stationary and personal indoor air situations.

**Methods:** First, we calibrated indoors against low volume active air samplers (LV-AAS) using the following PAS: unsheltered PDMS, styrene divenylbenzene (XAD) in a mesh pocket housing, and polyurethane foam (PUF) in a partial shelter. Second, we measured PDMS-air partition coefficients using GC retention times and estimated the capacity of PDMS to sample 76 SVOCs. Third, we used PDMS and partially-sheltered PUF to measure air concentrations of phthalates, brominated flame retardants (BFRs) and organophosphate esters (OPEs) in 51 Canadian homes. Fourth, we calibrated a PDMS brooch as personal PAS and tested its ability to measure personal air concentrations using participants from a convenience sample.

**Results:** Our findings in order of the methods are as follows:

1. PDMS had a higher mass transfer coefficient or uptake rate normalized to PAS surface area relative to the other PAS types.
2. PDMS equilibrium capacity ranged from ~ 1 day to ~ 500 years depending on a compound’s volatility.
3. We found no statistical difference between PDMS- and PUF-derived air concentrations when both were deployed in 51 homes. However, PUF had a higher detection frequency for particle-phase compounds. The greater particle capture may be, in part, because PUF has cavities for trapping particles, unlike PDMS which has a smooth surface.
4. PDMS brooch worn for ~ 1 work day (8 h) captured phthalates and OPEs but not BFRs which had 1000 times lower concentrations. Measurement of BFRs will require a longer deployment time to achieve analytical detection.

**Conclusion:** Silicone rubber or PDMS is being used increasingly as a passive sampler for estimating exposure to phthalates and a wide range of SVOCs. The data generated by these studies provide a foundation for interpreting results on SVOC exposures derived from deploying silicone rubber as stationary air samplers, brooches or wristbands.

### A31 A novel approach to examining multiple indoor exposures in early childhood on the inflammatory pathway and risk of intermediate outcomes for childhood asthma in the Canadian Healthy Infant Longitudinal Development (CHILD) birth cohort

#### Jaclyn Parks^1^, Tim K. Takaro^1^, Martine Vrijheid^2^. Xavier Basagaña^2^, Allan B. Becker^3^, James A. Scott^4^, Diana L. Lefebvre^5^, Wendy Lou^4^, Piush J. Mandhane^6^, Stuart E. Turvey^7^, Padmaja Subbarao^4,8^, Malcolm R. Sears^5^, Jeffrey R. Brook^2,9^

##### ^1^Simon Fraser University, Vancouver, Canada; ^2^ISGlobal, Barcelona, Spain; ^3^University of Manitoba, Winnipeg, Canada; ^4^University of Toronto, Toronto, Canada; ^5^McMaster University, Hamilton, Canada; ^6^University of Alberta, Edmonton, Canada; ^7^University of British Columbia, Vancouver, Canada; ^8^Hospital for Sick Children, Toronto, Canada; ^9^Environment Canada, Toronto, Canada

###### **Correspondence:** Tim K. Takaro (ttakaro@sfu.ca)

*Allergy, Asthma & Clinical Immunology* 2019, **15**(**Suppl 2**):A31

**Background:** Oxidizing chemicals found in the indoor environment, particularly consumer products, can cause inflammation leading to respiratory illness. The CHILD birth cohort is a national study examining how environmental exposures interact with genetics, immune system, microbiome and other personal characteristics to impact the risk of developing asthma and allergies.

**Methods:** 2700 of 3455 children born into the CHILD cohort were included in this analysis. A comprehensive suite of indoor exposures with potential inflammatory properties (e.g. mold and moisture, cleaning products and other household chemicals, and pests) in infancy were combined into an Indoor Environmental Exposure Index (IEEI). Exposures were evaluated against asthma-related outcomes of recurrent wheeze, atopy and diagnosed asthma at age 3 years. Three machine learning approaches were also used to calculate variable importance. An intersection of important variables (variables deemed important by all three models) was generated and a mean was calculated from each of the individual normalized scores. The individual exposures were grouped by IEEI domains and their normalized importance scores were averaged for each domain group.

**Results:** In logistic regression (adjusted for sex, parental atopy, income, ethnicity, and tobacco exposure) the IEEI at age 3 months was associated with a significantly higher risk of asthma and wheeze + atopy at 3 years of age in both continuous and interquartile comparisons, but not with atopy alone (see Table 1).

**Table 1:** Logistic regression results of the IEEI against outcomes at 3 years of age

IEEI as continuous variable			IEEI comparing upper to lower quartile	
Outcome	Odds ratio	95% conf. int.	Odds ratio	95% conf. int.
Asthma	1.39*	(1.03–1.86)	1.98*	(1.02–4.12)
Atopy	1.12	(0.94–1.33)	1.37	(0.91–2.07)
Wheeze + Atopy	1.56*	(1.03–2.38)	3.53*	(1.23–12.7)

*p-value ≤ 0.05

The machine learning approaches revealed similar exposure groups contributing to the outcomes, dominated by exposures to tobacco smoke, cleaning products, plastic foam, mould, other volatile chemicals and new furniture.

**Conclusions:** Infants with wheeze, and wheeze with atopy are at higher risk for developing asthma later in life. Our findings indicate that ubiquitous indoor exposures in the first 3 months of life may contribute to higher risk of wheeze and atopy, which may ultimately lead to asthma later in life. Given the longitudinal nature of CHILD, we can add exposure periods at later ages and track these relationships with more definitive asthma development.

**Acknowledgements:** This work is presented on behalf of the Canadian Healthy Infant Longitudinal Development (CHILD) Study.

### A32 Cotinine and environmental tobacco smoke in early life: An analysis from the Canadian Healthy Infant Longitudinal Development (CHILD) Study

#### Jaclyn Parks^1^, Kathleen McLean^2^, Jeffrey R. Brook^3^, Stuart E. Turvey^4^, Piush J. Mandhane^5^, Allan B. Becker^6^, Padmaja Subbarao^3,7^, Anita L. Kozyrskyj^5^, Meghan B. Azad^6^, Theo J. Moraes^7^, Diana L. Lefebvre^8^, Malcolm R. Sears^8^, James Scott^3^, Tim K. Takaro^1^

##### ^1^Faculty of Health Sciences, Simon Fraser University; ^2^BC Centers for Disease Control, Vancouver, Canada; ^3^Dalla Lana School of Public Health, University of Toronto, Toronto, Canada; ^4^Department of Pediatrics, University of British Columbia, Vancouver, Canada; ^5^Department of Pediatrics, University of Alberta, Edmonton, Canada; ^6^Department of Pediatrics & Child Health, University of Manitoba, Winnipeg, Canada; ^7^Department of Pediatrics, University of Toronto & Hospital for Sick Children, Toronto, Canada; ^8^Department of Medicine, McMaster University, Hamilton, Canada

###### **Correspondence:** Tim K. Takaro (ttakaro@sfu.ca)

*Allergy, Asthma & Clinical Immunology* 2019, **15**(**Suppl 2**):A32

**Introduction:** Asthma is the most common chronic disease of childhood, with environmental tobacco smoke a known important exposure. Cotinine is the most commonly used biomarker of tobacco smoke [3]. This study from the Canadian Healthy Infant Longitudinal Development (CHILD) birth cohort examines biomarkers of nicotine metabolites used to quantify environmental tobacco smoke exposure, and their ability to be validated by questionnaire responses related to smoking. The low level of detection and young study population allows the CHILD study to add valuable knowledge to this area of tobacco smoke exposure.

**Methods:** We examined urine samples taken at 3–4 months of age to measure cotinine (n = 986), and *trans*-3′-hydroxycotinine (3HC) (n = 1002). Following deconjugation, samples were analyzed by liquid chromatography-atmospheric pressure chemical ionization tandem mass spectrometry with a 0.03 ng/ml level of detection for each analyte. We explored their relationships to reported tobacco smoke exposure during pregnancy and in early life using logistic regression.

**Results:** After correcting for urine dilution, the geometric mean levels were 0.085 ng/mL for cotinine and 0.20 ng/mL for 3HC. 66% and 88% of infants with no reported early life smoke exposure had detectable levels of cotinine and 3HC, respectively. Only 3% reported tobacco smoke exposure during pregnancy and 5% reported household exposure during pregnancy or early life up to 3 months. The mean corrected cotinine concentrations in children whose mother smoked during pregnancy was 7.46 ng/mL, compared to 0.118 ng/mL in children whose mother did not. Questionnaire-based models incompletely predicted concentrations, explaining 43.4%, and 41.0% of the variance in cotinine, and 3HC levels, respectively.

**Conclusions:** These results suggest that tobacco smoke questionnaire models may not accurately predict cotinine concentrations at very low levels of detection. Tobacco smoke exposure models should use a combination of questionnaire and biomarker data to more accurately assess risk. Studies from the early 2000s found that while diet, particularly vegetables, can be a contributor of nicotine, the cotinine levels from diet were insignificant in relation to tobacco smoke exposure [2]. Rates of smoking during pregnancy have dropped significantly since then and detection levels have improved [1, 4, 5]. The role of diet as a contributor to cotinine concentrations [2] needs further exploration to inform the use of future cotinine research.

**Acknowledgements:** This work is presented on behalf of the Canadian Healthy Infant Longitudinal Development (CHILD) Study.

**Conflicts of interest:** None to declare.

**Funding sources:** Canadian Institutes of Health Research (CIHR), Allergy, Genes and Environment Network of Centers of Excellence (AllerGen NCE Inc.), and Asthma Canada.

**References**

1. Al-Sahab B, Saqib M, Hauser G, Tamim H. Prevalence of smoking during pregnancy and associated risk factors among Canadian women: a national survey. BMC Pregnancy Childbirth. 2010;10:24. 10.1186/1471-2393-10-24
2. Bramer S, Kallungal B. Clinical consideration in study designs that use cotinine as a biomarker. Biomarkers. 2003;8(3–4):187–203. 10.1080/13547500310012545
3. Centres for Disease Control and Prevention. Biomonitoring summary: cotinine. 2016; CAS No. 486-56-6. https://www.cdc.gov/biomonitoring/cotinine_biomonitoringsummary.html. Accessed 13 Nov 2017.
4. Connor SK, McIntyre L. The socio-demographic predictors of smoking cessation among pregnant women in Canada. Can J Public Health. 1999;90(5):352–5
5. Cui Y, Shooshtari S, Forget A, Clara I, Cheung K. Smoking during pregnancy: findings from the 2009–2010 Canadian Community Health Survey. PLoS ONE. 2014;9(1):e84640. 10.1371/journal.pone.0084640

### A33 Human rhinovirus infection of bronchial epithelial cells upregulates PGE2 production to modulate fibroblast-to-myofibroblast transition

#### Diana Pham^1,2^, Cora Kooi^1,2^, David Proud^2^, Richard Leigh^1,2^

##### ^1^Department of Medicine, University of Calgary, Calgary, Alberta, Canada; ^2^Department of Physiology and Pharmacology, University of Calgary, Calgary, Alberta, Canada

###### **Correspondence:** Diana Pham (diana.pham@ucalgary.ca)

*Allergy, Asthma & Clinical Immunology* 2019, **15**(**Suppl 2**):A33

**Background:** Fibroblast-to-myofibroblast transition (FMT) is a major contributor to airway remodeling in asthma. This phenomenon promotes the deposition of extracellular matrix (ECM) proteins in the lamina reticularis and subepithelial region, resulting in airway remodeling. Human rhinovirus (HRV) infection has been implicated in the pathogenesis of airway remodeling, in that HRV infection of human bronchial epithelial (HBE) cells results in the upregulation of growth factors involved in airway remodeling [1]. In previous experiments, we demonstrated that supernatants from HRV-infected HBE cells attenuated α-smooth muscle actin (α-SMA) production in human bronchial fibroblasts (HBF), and we now hypothesized that several mediators, including prostaglandin E_2_ (PGE_2_), are upregulated in HRV-infected HBE cell supernatants that modulate α-smooth muscle actin (α-SMA) production, as a marker of FMT [2]. The aim of this study was to investigate the role of HRV-infected HBE cells in upregulating PGE_2_ which, in turn, might modulate fibroblast differentiation.

**Methods:** HBE cells and HBF cells were isolated from non-transplanted normal human lungs, and infected with HRV-16 in a basal medium for 24 h. Supernatants were collected and added to HBF cells, alone and in combination with 3 ng/ml TGFβ1 (positive control) or PGE_2_ (10^−9^M–10^−6^M) for 48 h. HBF cells were analyzed for α-SMA immunostaining, and HBE cell supernatants were assessed for PGE_2_ production.

**Results:** HBF cells treated with HRV-infected HBE supernatants showed a significant attenuation in α-SMA protein expression compared to HBF cells treated with non-infected HBE supernatants (p ≤ 0.05). HRV-infected HBE supernatants were also able to inhibit TGFβ1-induced α-SMA protein (p ≤ 0.001), and PGE_2_ was upregulated in supernatants from HRV-infected HBEs. Moreover, HBF cells treated with increasing concentrations of PGE_2_ showed a progressive decrease in α-SMA protein expression.

**Conclusion:** Supernatants from HRV-infected epithelial cells attenuate TGFβ1-induced fibroblast α-SMA expression. HRV infection of HBF cells alone does not cause this attenuation; our data suggest that PGE_2_ upregulation in HRV-infected HBE supernatants may be responsible for this α-SMA inhibition. A better understanding of anti-fibrotic mechanisms may prove beneficial in developing therapies to prevent or treat airway remodeling in asthma.

**References**

1. Leigh R, Oyelusi W, Wiehler S, Koetzler R, Zaheer RS, Newton R, et al. Human rhinovirus infection enhances airway epithelial cell production of growth factors involved in airway remodeling. J Allergy Clin Immunol. 2008;121(5):1238–45.
2. Penke LRK, Huang SK, White ES, Peters-Golden M. Prostaglandin E2 inhibits a-smooth muscle actin transcription during myofibroblast differentiation via distinct mechanisms of modulation of serum response factor and myocardin-related transcription factor-A. J Biol Chem. 2014;289(24):17151–62.

### A34 Infant Th1 and Th2 immune activity and 18-month atopic dermatitis risk

#### Kharah M. Ross^1^, Gerald F. Giesbrecht^2^, Nicole Letourneau^1^

##### ^1^Faculty of Nursing, University of Calgary, Calgary, AB, T3B 2X9, Canada; ^2^Department of Paediatrics, University of Calgary, Calgary, AB, T3B 6A8, Canada

###### **Correspondence:** Kharah M. Ross (kharah.ross@ucalgary.ca)

*Allergy, Asthma & Clinical Immunology* 2019, **15**(**Suppl 2**):A34

**Background**: Risk for atopic dermatitis (AD), a common inflammatory skin disease characterized by itchiness and rash, could be driven in part by imbalance between the Th1 and Th2 adaptive branches of the immune system; specifically, a Th2 overbalance, relative to Th1 [e.g., 1–4]. Studies in this area have focused on differences in Th1 and Th2 cell populations and inflammatory markers produced by stimulated Th1 or Th2 cells in vitro [e.g., 5–11], with less known about the role of peripheral inflammatory markers as indicators of AD risk. Furthermore, those studies that do investigate peripheral inflammatory markers consider those markers separately [e.g., 12, 13], rather than modelling general Th1 or Th2 activity across subsets of markers. The purpose of this study was to test whether Th1 or Th2 indices, and Th1:Th2 balance, calculated from inflammatory markers measured in peripheral blood at 3 months of age, predicted risk for AD at 18 months of age.

**Methods:** Our sample consisted of 96 children, recruited as part of the Alberta Pregnancy Outcomes and Nutrition (APrON) Study. Mothers were recruited during pregnancy, and infants were assessed at 3 and 18 months postpartum. A multiplexing instrument was used to assay 11 inflammatory markers from blood samples collected at 3 months. Indices representing the Th1 and Th2 branches of the adaptive immune system were calculated by log-transforming, standardizing, and then averaging values of interferon (IFN)-γ, interleukin (IL)12p70 and IL2, and IL10, IL13, IL4 and IL5, respectively. The Th1:Th2 balance was modelled by calculating a Th1–Th2 interaction term by multiplying the indices together. At 18 months, mothers reported whether an AD diagnosis had been made by a physician. Considered covariates were maternal demographics and asthma diagnosis, gestational length, and child sex.

**Results:** Logistic regression models were used to test odds of AD diagnosis from Th1 and Th2 indices, and their interaction, controlling for covariates. Higher Th2 index values at 3 months predicted greater odds of AD diagnosis at 18 months, *b(SE) *= 1.5(0.65), *p *= 0.02, *OR *= 4.4. Neither the Th1 index nor the Th1–Th2 interaction term predicted AD diagnosis, *p*’s > 0.32.

**Conclusions:** Peripheral Th2 activity at 3 months predicted greater risk for an AD diagnosis at 18 months. Th1 activity was not associated with AD risk, nor the balance of the Th1 and Th2 indices. These findings have implications for understanding early indicators of AD risk, and the role of immune function in AD etiology.

**Acknowledgements:** The authors thank the APrON Study Team.

**References**

1. Eyerich K, Novak N. Immunology of atopic eczema: overcoming the Th1/Th2 paradigm. Allergy. 2013;68(8):974–82.
2. Grewe M, et al. A role for Th1 and Th2 cells in the immunopathogenesis of atopic dermatitis. Immunol Today. 1998;19(8):359–61.
3. Kidd P. Th1/Th2 balance: the hypothesis, its limitations, and implications for health and disease. Altern Med Rev. 2003;8(3):223–46.
4. Brandt EB, Sivaprasad U. Th2 cytokines and atopic dermatitis. J Clin Cell Immunol. 2011;2(3).
5. Thepen T, et al. Biphasic response against aeroallergen in atopic dermatitis showing a switch from an initial TH2 response to a TH1 response in situ: an immunocytochemical study. J Allergy Clin Immunol. 1996;97(3):828–37.
6. Chen L, et al. Early up-regulation of Th2 cytokines and late surge of Th1 cytokines in an atopic dermatitis model. Clin Exp Immunol. 2004;138(3):375–87.
7. Kanda N, et al. The skin fungus-induced Th1- and Th2-related cytokine, chemokine and prostaglandin E2 production in peripheral blood mononuclear cells from patients with atopic dermatitis and psoriasis vulgaris. Clin Exp Allergy. 2002;32(8):1243–50.
8. Magnan AO. Assessment of the Th1/Th2 paradigm in whole blood in atopy and asthma increased IFN- γ –producing CD8+ T cells in asthma. Am J Respir Crit Care Med. 2000;161.
9. Nilsson C, et al. Low numbers of interleukin-12-producing cord blood mononuclear cells and immunoglobulin E sensitization in early childhood. Clin Exp Allergy. 2004;34(3):373–80.
10. Prescott SL, et al. Neonatal interleukin-12 capacity is associated with variations in allergen-specific immune responses in the neonatal and postnatal periods. Clin Exp Allergy. 2003;33:566–72.
11. Smart JM, Kemp AS. Increased Th1 and Th2 allergen-induced cytokine responses in children with atopic disease. Clin Exp Allergy. 2002;32:796–802.
12. Shimada Y, Takehara K, Sato S. Both Th2 and Th1 chemokines (TARC/CCL17, MDC/CCL22, and Mig/CXCL9) are elevated in sera from patients with atopic dermatitis. J Dermatol Sci. 2004;34(3):201–8.
13. Piancatelli D, et al. Total IL-12 levels are increased in paediatric atopic dermatitis: correlations with age and disease severity. Int J Immunopathol Pharmacol. 2008;21(2):359–65.

### A35 Multiscale factors and early asthma incidence in the CHILD Study: Using Elastic Net regularization to integrate neighborhood level, individual level and gut microbiota sequencing data

#### Hind Sbihi^1,2^, Darlene Dai^1,2^, Rozlyn C.T. Boutin^3^, Charisse Petersen^3^, Chelsea J. Cutler^1^, Malcolm R. Sears^4^, Padmaja Subbarao^5,6^, Theo J. Moraes^5,6^, Allan B. Becker^7^, Meghan B. Azad^7^, Piush J. Mandhane^8^, B. Brett Finlay^3^, Stuart E. Turvey^1,2^

##### ^1^BC Children’s Hospital Research Institute, Vancouver, BC, Canada; ^2^Department of Pediatrics, Faculty of Medicine, University of British Columbia, Vancouver, BC, Canada; ^3^Department of Biochemistry and Molecular Biology, Faculty of Medicine, University of British Columbia, Vancouver, BC, Canada; ^4^Department of Medicine, Faculty of Health Sciences, McMaster University, Hamilton, ON, Canada; ^5^Department of Paediatrics, University of Toronto, Toronto, ON, Canada; ^6^Respiratory Medicine, the Hospital for Sick Children, Toronto, ON, Canada; ^7^Department of Pediatrics & Child Health and Community Health Sciences, University of Manitoba, Winnipeg, MB, Canada; ^8^Department of Pediatrics, Faculty of Medicine and Dentistry, University of Alberta, Edmonton, AB, Canada

###### **Correspondence:** Hind Sbihi (hind.sbihi@ubc.ca)

*Allergy, Asthma & Clinical Immunology* 2019, **15**(**Suppl 2**):A35

**Background:** In the Canadian Healthy Infant Longitudinal Development (CHILD) cohort, we investigated methods to integrate risk factors either known, modifiable (e.g., air pollution) or with putative effects (e.g., gut microbiota dysbiosis) on asthma incidence. In particular, we tackled the issue of high-dimensionality with rich correlation structure and often modest sample sizes [1, 2].

**Methods:** Stool samples collected at 3 and 12 months from an asthma-enriched CHILD subset (n = 1000) were profiled with 16S rRNA sequencing. Mothers’ built environment during pregnancy and in the first year of life was assessed at all residential addresses. Satellite measures of greenness (normalized difference vegetation index) and land-use regression models were used to derive individual estimates of nitrogen dioxide (NO_2_), a marker of traffic-related air pollution (TrAP). Data were collected on intrinsic risk factors, including parental asthma status, mode of delivery, parity, socio-economic factors, breastfeeding and antibiotic use. For feature selection we used elastic net, which is a regularization approach [2]. The best regularization parameter was selected based on leave-one-out cross validation (AUC > 0.6). The selected features were then entered in a conditional logistic regression to estimate the relative risks of asthma.

**Results:** In this subset, 10% (103) of children were diagnosed with asthma at age 3 years. Children with asthma were more likely born by Caesarean section, had a lower gestational age-adjusted birth weight, and a shorter duration of exclusive breastfeeding. Asthmatic children had higher TrAP exposure both in utero and during the first year of life when compared with non-asthmatic children (median NO_2_: 13.4 ppb and 11.6 ppb vs. 10.8 ppb and 9.1 ppb; p < 0.001 for both). Based on a random forest model trained on microbiome data from healthy children, we computed the gut maturity index [3], which represents the relative rate of change in “microbial age” and found a statistical difference between healthy (mean (sd) = 0.29 (1.06)) and asthmatic children (0.97 (1.37)). From a total of 104 features, the following 6 variables were selected using this unbiased approach (in decreasing order of importance): mode of delivery, parental ethnicity, antibiotic exposure after 3 months, gut maturity index, gut microbiome diversity (Faith’s PD) at 12 months, and TrAP exposure in utero.

**Conclusion:** Elastic net regressions offer the possibility of integrating factors that operate at varying scales, from within host to within neighborhood, and with different risk effects. When considered at the population level, addressing these factors could empower both significant large-scale preventative interventions as well as targeted individual measures.

**References**

1. Burbank AJ, Sood AK, Kesic MJ, Peden DB, Hernandez ML. Environmental determinants of allergy and asthma in early life. J Allergy Clin Immunol. 2017;140(1):1–12.
2. Kirpich A, Ainsworth EA, Wedow JM, Newman JRB, Michailidis G, McIntyre LM. Variable selection in omics data: a practical evaluation of small sample sizes. PLoS ONE. 2018;13(6):e0197910.
3. Stokholm J, Blaser MJ, Thorsen J, Rasmussen MA, Waage J, Vinding RK, et al. Maturation of the gut microbiome and risk of asthma in childhood. Nat Commun. 2018;9(1):141.

### A36 When does exposure matter for childhood allergic disorders? Temporal trends in greenness in four Canadian cities

#### Hind Sbihi^1,2^, Lorien Nesbitt^3^, Perry Hystad^4^, Jeff R. Brook^5^, Malcolm R. Sears^6^, Padmaja Subbarao^7,8^, Theo J. Moraes^7,8^, Piush J. Mandhane^9^, Allan B. Becker^10^, Meghan B. Azad^10^, Matilda Van den Bosch^3^, Stuart E. Turvey^1,2^

##### ^1^BC Children’s Hospital Research Institute, Vancouver, BC, Canada; ^2^Department of Pediatrics, Faculty of Medicine, University of British Columbia, Vancouver, BC, Canada; ^3^School of Population and Public Health, Faculty of Medicine, University of British Columbia, Vancouver, BC, Canada; ^4^College of Public Health and Human Sciences, Oregon State University, Portland, OR, USA; ^5^Dalla Lana School of Public Health, University of Toronto, Toronto, ON, Canada; ^6^Department of Medicine, Faculty of Health Sciences, McMaster University, Hamilton, ON, Canada; ^7^Department of Paediatrics, University of Toronto, Toronto, ON, Canada; ^8^Respiratory Medicine, the Hospital for Sick Children, Toronto, ON, Canada; ^9^Department of Pediatrics, Faculty of Medicine and Dentistry, University of Alberta, Edmonton, AB, Canada; ^10^Department of Pediatrics & Child Health and Community Health Sciences, University of Manitoba, Winnipeg, MB, Canada

###### **Correspondence:** Hind Sbihi (hind.sbihi@ubc.ca)

*Allergy, Asthma & Clinical Immunology* 2019, **15**(**Suppl 2**):A36

**Background:** Urban vegetation plays an important role in reducing the harmful effects incurred through the process of urbanization: it removes harmful pollutants from the air and provides cooling during extreme heat events [1]. Further, mounting evidence shows inconsistent links to childhood allergic outcomes [2]. These contradictory results may be due to the methods chosen to assess greenness exposures. Here, we aim to test the temporal stability of greenness exposures in order to generate best estimates in the context of epidemiological studies of childhood health outcomes.

**Methods:** We evaluated changes in greenness at the home addresses of all participants recruited into the Canadian Healthy Infant Longitudinal Development (CHILD) birth cohort and followed up to age 5 years old (n = 3054). Greenness was evaluated using various data sources between 2008 and 2015—dates representing the period between conception and age 5 for most CHILD participants—including Normalized Difference Vegetation Index (NDVI)-based MODIS satellite images, proximity measures, and street-level measures. In order to pinpoint relevant time periods for examining the effect of greenness exposure on allergic outcomes, exposure-responses were examined for various time periods: in utero, first year of life, and lifetime cumulative exposure.

**Results:** All cities showed distinct greenness patterns over time for assigned NDVI in a 500 m buffer around the residential addresses of CHILD participants. However, from 2010 onward increasing median values were observed, except for Edmonton. For each year between 2008 and 2015, the spread of NDVI values varied distinctly for each CHILD study site with Toronto and Winnipeg having the lowest interquartile ranges (Winnipeg: [0.05–0.09]; Toronto: [0.04–0.06]). Adjusting for the season at time of birth, there were no statistically significant associations with asthma diagnosed at age 5, whereas the odds of atopic rhinitis at age 3 (Odds Ratio (OR): 0.86, p-val = 0.005) and sensitization outcomes (OR = 0.88, p-val = 0.04 at age 1 and OR = 0.87, p-val = 0.04 at age 3) were reduced following one interquartile increase of perinatal exposure to greenness.

**Conclusion:** We find that greenness is associated with allergic outcomes but not asthma, when using NDVI as a metric for greenness. These results warrant the following investigations: (i) are these associations robust for other metrics?; (ii) what are the determinants of temporal change in greenness?; and (iii) how do different time windows of exposure impact trajectories of sensitization?

**References**

1. Fong KC, Hart JE, James P. A review of epidemiologic studies on greenness and health: updated literature through 2017. Curr Environ Health Rep. 2018;5(1):77–87.
2. Tischer C, Gascon M, Fernandez-Somoano A, Tardon A, Materola AL, Ibarluzea J, et al. Urban green and grey space in relation to respiratory health in children. Eur Respir J. 2017;49(6):1502112.

### A37 An interactive environment for visualizing multi-OMIC integrative models

#### C. P. Shannon^1,2^, J. Ye^1,2^, A. Singh^1,2^, S. J. Tebbutt^1,2,3^

##### ^1^Centre for Heart Lung Innovation, UBC, Vancouver, British Columbia, Canada; ^2^Prevention of Organ Failure (PROOF) Centre of Excellence, UBC, Vancouver, British Columbia, Canada; ^3^Division of Respiratory Medicine, Department of Medicine, UBC, Vancouver, British Columbia, Canada

###### **Correspondence:** C. P. Shannon (casey.shannon@hli.ubc.ca)

*Allergy, Asthma & Clinical Immunology* 2019, **15**(**Suppl 2**):A37

**Background:** Technological advances now allow for simultaneous profiling of many biological compartments, such as the genome, transcriptome, proteome, and metabolome. Traditional analytical approaches may ignore the interactions that exist between these OMIC compartments. There is great interest in applying novel, integrative analytical frameworks to such data. Integrative multi-OMIC models can be complex and difficult to interpret, however. We developed a software application that allows researchers to interactively explore and visualize the key relationships that exist in their multi-OMIC data, as identified by the multi-OMIC integrative framework, DIABLO (Data Integration Analysis for Biomarker discovery using Latent cOmponents). This application was applied to an AllerGen Clinical Investigator Collaborative asthma study.

**Hypothesis:** Integrative multi-OMIC models, such as those fit by DIABLO, may provide novel perspective and greater insights when compared to traditional single-OMIC approaches. Exploration of such models through interactive visualizations can improve their interpretability and facilitate hypothesis generation.

**Methods:** With the decision to create an interactive web application, software was developed with a simple, visually pleasing user interface along with the corresponding backend server code. The application was developed primarily in R using Shiny, a web application framework. The application focuses on DIABLO, a multi-OMIC data integration framework. Some existing plotting functions were modified to allow for the interactive aspect of the application. The asthma dataset consisted of pre- and post-allergen inhalation challenge blood samples from 14 research participants who had mild allergic asthma. Datasets included cell composition, plasma metabolomics and leukocyte transcriptomics.

**Results:** Using a newly developed network visualization to explore interactions between the key molecular and cellular drivers identified by DIABLO to discriminate pre- from post-challenge samples, we determined a set of features including basophil cells, as well as genes and metabolites involved in branched chain amino acids and butanoate metabolism.

**Conclusion and future directions:** Longer-term goals include additional functionality to aid in hypothesis generation, such as the ability to interface with existing biological knowledge repositories; e.g., MSigDB gene set collections, or the InnateDB protein–protein interaction database.

*This project is funded by AllerGen and the National Institute of Allergy and Infectious Diseases.*

### A38 Geographical variation in aeroallergen concentrations across four Canadian cities

#### Cecilia Sierra-Heredia^1^, Ryan Allen^1^, Sarah B. Henderson^2,3^, Frances Coates^4^, Jordan Brubacher^1^, Michelle North^5^, Éric Lavigne^6^, Hind Sbihi^3^, Michael Brauer^3^, Malcolm R. Sears^7^, Jeffrey R. Brook^8,9^, James Scott^9^, Diana L. Lefebvre^7^, Meghan Azad^10^, Allan B. Becker^11^, Piush J. Mandhane^12^, Theo Moraes^13^, Stuart E. Turvey^14^, Padmaja Subbarao^13^, Tim K. Takaro^1^

##### ^1^Faculty of Health Sciences, Simon Fraser University, Burnaby, Canada; ^2^BC Centre for Disease Control, Vancouver, Canada; ^3^School of Population and Public Health, University of British Columbia, Vancouver, Canada; ^4^Aerobiology Research Laboratories, Ottawa, Canada; ^5^Novartis, Toronto, Canada; ^6^Air Health Science Division, Health Canada; ^7^Department of Medicine, Faculty of Health Sciences, McMaster University, Toronto, Canada; ^8^Air Quality Research Division, Environment Canada, Toronto, Canada; ^9^Dalla Lana School of Public Health, University of Toronto, Toronto, Canada; ^10^Department of Pediatrics & Child Health and Community Health Sciences, University of Manitoba, Winnipeg, Canada; ^11^Department of Immunology, Faculty of Medicine, University of Manitoba, Winnipeg, Canada; ^12^Department of of Pediatrics, Faculty of Medicine and Dentistry, University of Alberta, Edmonton, Canada; ^13^Hospital for Sick Children, Department of Paediatrics, Faculty of Medicine, University of Toronto, Toronto, Canada; ^14^Department of Paediatrics, Faculty of Medicine, University of British Columbia, Vancouver, Canada

###### **Correspondence:** Cecilia Sierra-Heredia (sierrahe@sfu.ca)

*Allergy, Asthma & Clinical Immunology* 2019, **15**(**Suppl 2**):A38

**Background:** Pollen grains from anemophilous plants and fungal spores represent an important class of aeroallergens in ambient air. High concentrations of aeroallergens are associated with increased asthma-related emergency department visits and hospitalizations across Canada. Whether exposure in early life leads to the development of asthma is unknown. The geographical distribution of species of plants is dependent on multiple factors, including precipitation, soil characteristics and temperatures. Five “floristic zones” that support the growth of different aeroallergen-producing plants have been defined across Canada. In this study, we describe the geographic variations for the most important aeroallergens during (1) the years in which the Canadian Healthy Infant Longitudinal Development (CHILD) birth cohort participants were born (i.e. 2008–2012) and (2) in the four cities where they live, Vancouver, Edmonton, Winnipeg and Toronto.

**Methods:** We examined the differences among four Canadian cities located in three of the Canadian floristic zones with respect to season length and annual average concentration between 2008 and 2012. These cities and years correspond to the antenatal and perinatal windows of sensitization for the participants in the CHILD birth cohort urban population. Pollen grain and fungal spores were collected with a 10% sampling period over a 24-h period and then aggregated in weekly averages. Aeroallergen season was defined by the dates when the 5% and 95% of the annual total was recorded for each pollen and fungi taxa.

**Results:** Vancouver had the highest cumulative pollen concentration for coniferous and deciduous trees. The concentrations of ascomycetes, basidiomycetes and fungi imperfecti were much higher in Edmonton, Winnipeg and Toronto than Vancouver (Table 1). Consistent with the more moderate climates, Vancouver had a longer season for every group of pollen and spores except grass, where Toronto had the longest season. Toronto had the second longest season for weeds, deciduous and coniferous pollen and the three groups of spores.

**Table 1:** Five-year mean and range of pollen and spore cumulative concentrations (grains/m3)

	Vancouver	Edmonton	Winnipeg	Toronto
Pollens Mean [min, max] (in hundreds)				
Coniferous	210 [126, 364]	20 [10, 39]	32 [20, 56]	36 [19, 52]
Deciduous	218 [110, 308]	53 [29, 108]	136 [95, 180]	131 [90, 172]
Grasses	15 [11, 20]	15 [8, 25]	16 [8, 24]	10 [9–11]
Weeds	8 [7, 9]	14 [10, 17]	29 [23, 37]	31 [28, 34]
Spores Mean [min, max] (in hundreds)				
Ascomycetes	1420 [726, 2132]	1619 [543, 2362]	1558 [1274, 2171]	964 [712, 1246]
Basidiomycetes	1429 [887, 1754]	1170 [389, 1650]	1229 [852, 1500]	1012 [917, 1179]
Fungi imperfecti	1490 [1235, 2050]	2815 [2349, 2824]	3460 [2862, 4534]	1683 [1228, 2080]

**Conclusion:** There was wide variation in aeroallergen concentrations across the CHILD recruitment cities. These geographical differences in concentrations may be important in the development of atopy and asthma. Local climate context is needed when designing public health initiatives related to aeroallergens and research on the development of atopic conditions.

### A39 Estrogen effects on Th2 cell phenotype: key to severe asthma in women?

#### Lauren A. Solomon^1^, Tharsan Kanagalingam^1^, Meerah Vijeyakumaran^1^, Nami Shrestha Palikhe^2^, Harissios Vliagoftis^2^, Lisa Cameron^1,2^

##### ^1^Department of Pathology and Laboratory Medicine, Western University, London, ON, N6A 3K7, Canada; ^2^Pulmonary Research Group, Department of Medicine, University of Alberta, Edmonton, AB, T6G 2R3, Canada

###### **Correspondence:** Lauren A. Solomon (Lsolomo3@uwo.ca)

*Allergy, Asthma & Clinical Immunology* 2019, **15**(**Suppl 2**):A39

**Background:** Allergic asthma is a T helper 2 (Th2) cell-associated inflammatory disease, driven by cytokines such as IL-4, IL-5, and IL-13. Th2 cells express the G-protein-coupled receptor CRTh2, a receptor for prostaglandin D_2_ (PGD_2_) that influences Th2 function and survival. Inhaled glucocorticosteroids are the primary treatment of allergic asthma and improve asthma symptoms by inhibiting Th2 cytokine production and at high levels by killing Th2 cells. Women are more likely than men to have severe asthma and to have symptoms requiring a hospital visit. We observed that severe asthmatic women have more circulating Th2 cells than men with severe asthma, despite taking similar doses of inhaled glucocorticosteroid [1]. These findings lead us to consider a mechanism by which female sex hormones could influence Th2 cell response to glucocorticosteroid.

**Methods:** Using whole-mRNA sequencing, we examined gene expression in primary Th2 cells following exposure to glucocorticosteroids (0.1 µM) in the presence or absence of an estrogen mimic, PPT (10 µM). Gene expression changes were validated by quantitative qPCR. Prostaglandin D_2_ levels in culture supernatant were determined by ELISA.

**Results:** Gene expression in primary Th2 cells was examined following exposure to glucocorticosteroids in the presence or absence of an agonist for estrogen receptor alpha (ERα), PPT. While glucocorticosteroids repressed Th2 cytokines, regardless of addition of PPT, many glucocorticosteroid-mediated effects were suppressed or even counter-acted by PPT. These included increased expression of genes identifying a “pathogenic” Th2 subset characterized by high levels of CRTh2, hPGDS and CD161. Validation by ELISA demonstrated a significant increase in prostaglandin D_2_ in the culture media following treatment with a combination of glucocorticosteroid and PPT compared to glucocorticosteroid alone. Anti-apoptotic genes including BCL2 were also increased by co-treatment with the agonist and glucocorticosteroid. Functional studies are now planned to examine whether the combination of glucocorticosteroid and estrogen treatment results in a feed-forward, pro-survival loop involving PGD_2_-CRTh2 signaling.

**Conclusions:** These findings suggest that the effects of glucocorticosteroids on Th2 cells are influenced by estrogen signaling which, in women, could represent a mechanism driving steroid insensitivity and development of severe asthma.

**Acknowledgements:** This work was supported by AllerGen NCE Inc. (the Allergy, Genes and Environment Network), a member of the Networks of Centres of Excellence Canada program.

**Reference**

1. Palikhe NS, Laratta C, Nahirney D, Vethanayagam D, Bhutani M, Vliagoftis H, Cameron L. Elevated levels of circulating CD4(+) CRTh2(+) T cells characterize severe asthma. Clin Exp Allergy. 2016;46(6):825–36.

### A40 Understanding the interplay between the lung microbiota and pulmonary innate immune cell function

#### Ajitha Thanabalasuriar^1,2^, Tina Sakar^1^, Paul Kubes^1^

##### ^1^University of Calgary Department of Pharmacology and Physiology; ^2^MedImmune/Astrazeneca Infectious Disease Unit

###### **Correspondence:** Paul Kubes (pkubes@ucalgary.ca)

*Allergy, Asthma & Clinical Immunology* 2019, **15**(**Suppl 2**):A40

**Introduction:** The human body is host to numerous microbial communities. An important and often overlooked microbial community exists in the respiratory tract [1, 2]. Like the gut, the lung has a unique resident population of microbes at its mucosal surface. These microbes exist in the upper and lower airways, and are independent of the oral and gut microbiota [2]. We hypothesize that the lung microbiota plays an important role in the development of pulmonary innate immune cells, and “dysbiosis” of the lung microbiota can lead to a perturbed pulmonary innate immune environment.

**Methods:** We use a combination of flow and mass cytometry, and intravital microscopy to characterize the immune cell landscape in the lungs of mice. We use 16S rDNA and bacterial cultures to understand the lung microbiota. We use a brain-tree aerosolization system to target lung microbiota specifically without affecting microbes in the gut.

**Results:** In this study we show that modulating the lung microbiota results in changes to pulmonary immune cells. Specifically, aerosolized antibiotic treatment in neonates modulated pulmonary invariant natural killer T (iNKT) cell numbers in the lungs, increasing them dramatically. This marked increase in iNKT cell numbers persisted once the mice were taken off antibiotic treatment. In fact, the iNKT cell numbers stayed dysregulated well into adulthood. iNKT cells are potent regulators of inflammation in the lung. Dysregulation of iNKT cells can result in overt inflammation and the development of fibrosis and/or susceptibility to bacterial infections such as *Streptococcus pneumoniae* infections [3, 4].

**Conclusion:** As we learn more about the lung microbiota it is clear that in chronic lung disease patients, such as individuals with Asthma or Chronic Obstructive Pulmonary Disease, the lung microbiota is dysregulated. Moderating pulmonary immune cell function with inhaled probiotics may be a promising new therapy that could possibly prevent the development of chronic diseases, such as asthma in children. On the other hand, targeting the clearance of ‘bad bacteria’ in the lungs of chronic lung disease patients, such COPD patients, may reprogram the host immune system to relieve overt inflammation and decrease the severity of disease.

**References**

1. Barford KK, Vrankx K, Mirsepasi-Lauridsen C, Hansen SJ, Hougaard SK, Larsen TA, Puwenhand CA, Krogfelt AK. The murine lung microbiome changes during lung inflammation and intranasal vancomysin treatment. Open Microbiol J. 2015;9:167–79.
2. Mathur S, Fuchs A, Bielicki J, Van Den Anker J, Sharland M. Antibiotic use for community-acquired pneumonia in neonates and children: WHO evidence review. Paediatr Int Child Health. 2018;38:S66–S75.
3. Thanabalasuriar A, Neupane AS, Wang J, Krummel MF, Kubes P. iNKT cell emigration out of the lung vasculature requires neutrophils and monocyte-derived dendritic cells in inflammation. Cell Rep. 2016;16:3260–72.
4. Kawakami K, et al. Critical role of Valpha14+ natural killer T cells in the innate phase of host protection against *Streptococcus pneumoniae* infection. Eur J Immunol. 2003;33:3322–30.

### A41 PD-L1^+^ regulatory B cells increase during milk oral immunotherapy

#### Bahar Torabi^1^, Marieme Dembele^2^, Duncan Lejtenyi^2^, Ingrid Baerg^3^, Edmond S. Chan^3^, Moshe Ben-Shoshan^4^, Bruce D. Mazer^2,4^

##### ^1^Department of Pediatrics, University of British Columbia, Vancouver, BC, Canada; ^2^The Research Institute of the McGill University Health Centre, Montreal, QC, Canada; ^3^Division of Allergy and Immunology, Department of Pediatrics, BC Children’s Hospital, Vancouver, BC, Canada; ^4^Division of Pediatric Allergy and Clinical Immunology, Montreal Children’s Hospital, Montreal, QC, Canada

###### **Correspondence:** Bahar Torabi (bahar.torabi@mail.mcgill.ca)

*Allergy, Asthma & Clinical Immunology* 2019, **15**(**Suppl 2**):A41

**Background:** Regulatory B cells (Bregs) have been implicated in venom immunotherapy, non-IgE-mediated food allergies, and autoimmune diseases [1–3]. No studies have examined the correlation between Bregs and IgE-mediated milk allergy, nor have the action of Bregs been examined in the treatment of food allergies with oral immunotherapy (OIT). Programmed death-ligand 1 (PD-L1) is one of the surface molecules described on Bregs [4]. We aimed to determine if children with IgE-mediated milk allergy who have successfully reached the end of escalation phase of milk OIT have an increase in Bregs.

**Methods:** Peripheral blood mononuclear cells were isolated from plasma of milk-allergic children undergoing milk OIT, at baseline and at the end of escalation phase (200 ml). Cells were cultured for 72 h in various conditions and stained for CD19, CD27, CD38, CD5, CD24, PD-L1, and intracellular IL-10. Statistical analysis was done using the Wilcoxon matched-pairs signed rank test.

**Results:** Bregs were analyzed in 9 patients at baseline and end of escalation phase (200 ml time point). There was a significant increase in 7 out of 9 patients in the percentage of CD19intPD-L1+ and CD19intPD-L1+ CD38+ populations at the end of escalation phase in 3 conditions: CpG-B/anti-IgM/IgG/αCD40, αCD40/IL4/IL21, and αCD40/IL4/IL21 plus milk proteins (Fig. 1).

**Fig. 1:**
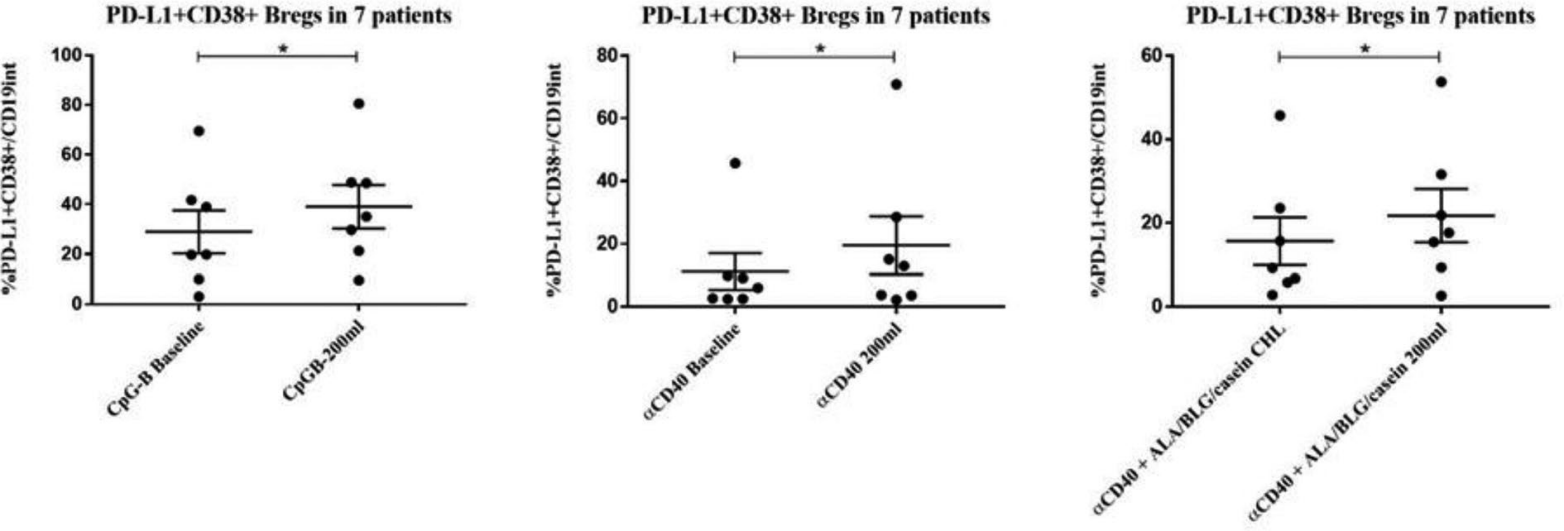
PD-L1^+^CD38^+^Bregs at baseline and 200 ml in 3 conditions in 7 subjects showing an increase, *p < 0.05

The majority (89.16%, 95% CI 81.21–95.56%) of the CD19intPD-L1+ population in all conditions were CD38+ cells. The 2 subjects demonstrating a decrease in the PD-L1 population had a higher baseline percentage in all conditions. They also ingested the highest cumulative volume of milk during their challenge compared to the 7 other patients (89.4 ml and 149.4 ml vs median 14.4 ml).

**Conclusion:** PD-L1+ Bregs increase during milk OIT and may be part of the mechanism of successful desensitization in children. This population of Bregs could play a role in other allergic diseases as well. Further assessment with a larger sample size is required.

**Acknowledgements:** This project was supported by the Richard and Edith Strauss Clinical Fellowship, AllerGen, GET-FACTS, and McGill University. I would like to acknowledge the members of the Mazer lab at the Meakins-Christie Laboratories, the nursing team at the CIM at the RI-MUHC, the clinical collaboration at BC Children’s Hospital, Ste-Justine, Chicoutimi, and SickKids, as well as the participating patients and their families.

**References**

1. van de Veen W, Stanic B, Yaman G, Wawrzyniak M, Sollner S, Tec S, Akdis DG, Ruckert B, Akdis C, Akdis M. IgG4 production is confined to human IL-10-producing regulatory B cells that suppress antigen-specific immune responses. J Allergy Clin Immunol. 2013;131:1204–12.
2. Duddy M, Niino M, Adatia F, Hebert S, Freedman M, Atkins H, Kim HJ, Bar-Or A. Distinct effector cytokine profiles of memory and naive human B cell subsets and implication in multiple sclerosis. J Immunol. 2007;178(10):6092–9.
3. Noh J, Noh G, Kim HS, Kim AR, Choi WS. Allergen-specific responses of CD19(+)CD5(+)Foxp3(+) regulatory B cells (Bregs) and CD4(+)Foxp3(+) regulatory T cell (Tregs) in immune tolerance of cow milk allergy of late eczematous reactions. Cell Immunol. 2012;274:109–14.
4. Khan AR, Hams E, Floudas A, Sparwasser T, Weaver CT, Fallon PG. PD-L1hi B cells are critical regulators of humoral immunity. Nat Commun. 2015;6:5997.

### A42 A symptom-based algorithm to improve asthma diagnosis in early childhood: the Canadian Healthy Infant Longitudinal Development (CHILD) Study

#### Maxwell M. Tran^1,†^, Ruixue Dai^1,2,†^, Wendy Y.W. Lou^2^, Melanie Emmerson^1^, Christoffer Dharma^3^, Diana Lefebvre^3^, Theo J. Moraes^1^, Allan B. Becker^4^, Elinor Simons^4^, Piush J. Mandhane^5^, Stuart E. Turvey^6^, Malcolm R. Sears^3,‡^, Padmaja Subbarao^1,2,‡^

##### ^1^Department of Pediatrics, The Hospital for Sick Children, Toronto, Canada; ^2^Dalla Lana School of Public Health, University of Toronto, Toronto, Canada; ^3^Department of Medicine, McMaster University, Hamilton, Canada; ^4^Department of Pediatrics & Child Health, University of Manitoba, Winnipeg, Canada; ^5^Department of Pediatrics, University of Alberta, Edmonton, Canada; ^6^Department of Pediatrics, BC Children’s Hospital and The University of British Columbia, Vancouver, Canada

###### **Correspondence:** Maxwell M. Tran (tran.maxwell@gmail.com)

^†^Co-first authors, ^‡^Co-senior authors

*Allergy, Asthma & Clinical Immunology* 2019, **15**(**Suppl 2**):A42

**Background:** Many children experience early childhood wheezing, but remission is common and most do not develop persistent asthma. Our objective was to develop a short, symptom-based algorithm to screen symptomatic 3-year old children to detect those at risk of persistent wheeze at 5 years.

**Methods:** Parents of children participating in the Canadian Healthy Infant Longitudinal Development (CHILD) pregnancy cohort study completed comprehensive health questionnaires at multiple time points. Children underwent structured clinical assessments at ages 1, 3 and 5 years. Diagnoses of asthma by a Study pediatrician were documented at 3 and 5 years.

**Results:** At age 3 years, four operational definitions of asthma were compared: (a) the in-person clinical assessment by an experienced Study pediatrician, (b) a parental report of physician-diagnosed asthma, (c) the modified Asthma Predictive Index (mAPI) [1, 2], and (d) a symptom-based algorithm using questionnaire data, incorporating nature and frequency of symptoms in the last year and documentation of supporting criteria (emergency department visits, hospitalization, use of asthma medications, and frequent dry cough). Among 2354 children, a classification of “definite asthma by algorithm,” compared to “no asthma by algorithm,” had the strongest associations with study physician-diagnosed asthma at age 5 years (OR 50.4, 95% CI 29.7–89.3) and persistent wheeze to age 5 years (OR 239, 95% CI 114–583). The algorithm identified more cases of definite asthma and more unique cases not identified by the other definitions. Notably, of 79 children with persistent wheeze to age 5 years, the algorithm identified 72 (91.1%), whereas in-person clinical assessment identified 49 (62.0%), and parental report of physician-diagnosed asthma only 33 (41.8%). The mAPI could be applied in 68 of these 79 children, of whom 33 (48.5%) were mAPI positive.

**Conclusions:** A short, simple symptom-based algorithm identified the great majority of children at age 3 years with likely asthma and at higher risk for persistence to age 5 years. The algorithm could be incorporated in electronic medical records to alert physicians to children requiring further evaluation and management of asthma.

**Acknowledgements:** The authors are grateful to the families who participated in this study, the CHILD Study team, Don & Debbie Morrison, and AllerGen NCE Inc. (the Allergy, Genes and Environment Network), a member of the Networks of Centres of Excellence Canada program.

**References**

1. Castro-Rodríguez JA, Holberg CJ, Wright AL, Martinez FD. A clinical index to define risk of asthma in young children with recurrent wheezing. Am J Respir Crit Care Med. 2000;162:1403–6.
2. Guilbert TW, Morgan WJ, Zeiger RS, Bacharier LB, Boehmer SJ, Krawiec M, et al. Atopic characteristics of children with recurrent wheezing at high risk for the development of childhood asthma. J Allergy Clin Immunol. 2004;114(6):1282–7.

### A43 Delayed gut microbiota maturation during infancy is associated with food sensitization in children

#### Hein M. Tun^1,2^, Theodore Konya^3^, Radha Chari^4^, Catherine J. Field^5^, David S. Guttman^6^, Allan B. Becker^7^, Meghan B. Azad^7^, Piush J. Mandhane^1^, Theo J. Moraes^8^, Malcolm R. Sears^9^, Stuart E. Turvey^10^, Padmaja Subbarao^8^, James A. Scott^2^, Anita L. Kozyrskyj^1^

##### ^1^Department of Pediatrics, University of Alberta, Edmonton, AB, Canada; ^2^HKU-Pasteur Research Pole, School of Public Health, University of Hong Kong, Hong Kong; ^3^Dalla Lana School of Public Health, University of Toronto, Toronto, ON, Canada; ^4^Department of Obstetrics and Gynecology, University of Alberta, Edmonton, AB, Canada; ^5^Department of Agricultural, Food & Nutritional Science, University of Alberta, Edmonton, AB, Canada; ^6^Centre for the Analysis of Genome Evolution and Function, University of Toronto, Toronto, ON, Canada; ^7^Department of Pediatrics & Child Health, Children’s Hospital Research Institute of Manitoba, University of Manitoba, Winnipeg, MB, Canada; ^8^Department of Pediatrics, Hospital for Sick Children, University of Toronto, Toronto, ON, Canada; ^9^Department of Medicine, McMaster University, Hamilton, ON, Canada; ^10^Department of Pediatrics, Child & Family Research Institute, BC Children’s Hospital, University of British Columbia, Vancouver, BC, Canada

###### **Correspondence:** Anita L. Kozyrskyj (kozyrsky@ualberta.ca)

*Allergy, Asthma & Clinical Immunology* 2019, **15**(**Suppl 2**):A43

**Background:** Infancy is a critical stage for establishing endogenous gut microbiota, seen as successional changes to microbiota over time [1]. Previous studies have already demonstrated the association between infant gut microbiota and food sensitization in childhood [2–4]. However, the evidence was mainly on microbiota signatures at cross-sectional time points, and an extended question has not yet been answered on whether developmental trajectories of infant gut microbiota predict food sensitization in later childhood. In this study, we determined gut microbial compositional changes over time and tested their association with food sensitization.

**Method:** In a large sub-sample of 1422 infants from the Canadian Healthy Infant Longitudinal Development (CHILD) birth cohort, microbial compositions of infant fecal samples at age 3 months (early infancy) and 12 months (late infancy) were characterized using 16S rRNA sequencing in Illumina MiSeq platform, followed by the partitioning around medoids clustering of Jensen-Shannon divergence distance. Food sensitization was determined by skin prick testing to peanut, egg and milk at ages 1 and 3 years. Covariates were derived from hospital records and standardized questionnaires and used in multivariable model adjustments.

**Results:** Two microbiota clusters were characteristic of early infancy (dominated by Actinobacteria and Proteobacteria) and late infancy (dominated by Bacteroidetes and Firmicutes). A total of 529 infants progressed from the early to the late profile (labelled ‘expected maturation’), 650 infants were found in the late profile at both ages (‘early maturation’) and 220 retained an early profile (‘delayed maturation’). Twenty-two infants with a late profile at 3 months later reverted to an early profile. Compared to ‘expected maturation’, ‘delayed maturation’ was associated with food sensitization at ages 1 (OR 1.8, 95% CI 1.1–2.9) and 3 years (OR 4.4, 95% CI 1.1–17.4), independent of birth mode, exclusive breastfeeding, and maternal overweight, major factors that influence microbial profile change (P < 0.05).

**Conclusion:** Our study provides new evidence that delayed gut microbiota maturation over infancy may represent a risk factor for childhood food sensitization. Microbial biomarkers identified in this study are of potential future use in interventions to protect children against development of food allergies.

**Acknowledgements:** We would like to thank all the families who took part in this study, and the whole CHILD team including the CHILD study investigators.

**References**

1. Backhed F, Roswall J, Peng Y, Feng Q, Jia H, Kovatcheva-Datchary P, et al. Dynamics and stabilization of the human gut microbiome during the first year of life. Cell Host Microbe. 2015;17(5):690–703.
2. Ling Z, Li Z, Liu X, Cheng Y, Luo Y, Tong X, et al. Altered fecal microbiota composition associated with food allergy in infants. Appl Environ Microbiol. 2014;80(8):2546–54.
3. Azad MB, Konya T, Guttman DS, Field CJ, Sears MR, HayGlass KT, et al. Infant gut microbiota and food sensitization: associations in the first year of life. Clin Exp Allergy. 2015;45(3):632–43.
4. Tanaka M, Korenori Y, Washio M, Kobayashi T, Momoda R, Kiyohara C, et al. Signatures in the gut microbiota of Japanese infants who developed food allergies in early childhood. FEMS Microbiol Ecol. 2017;93(8).

### A44 Intestinal fungi impact the gut bacterial microbiome and elicit host immune changes related to atopy

#### Erik van T. Bernardes^1,2^, Isabelle Laforest-Lapointe^1,2^, Shen Y. Tsou^1,2^, Haley Jamison^1,2^, Kathy McCoy^1^, Marie-Claire Arrieta^1,2^

##### ^1^Dept of Physiology & Pharmacology, University of Calgary, Calgary, T2N 4N1, Canada; ^2^Dept of Paediatrics, University of Calgary, Calgary, T2N 4N1, Canada

###### **Correspondence:** Erik van T. Bernardes (evtberna@ucalgary.ca)

*Allergy, Asthma & Clinical Immunology* 2019, **15**(**Suppl 2**):A44

**Background:** Neonatal immunity undergoes important developmental changes that are dependent on microbial colonization post-birth [1]. Early-life gut microbial dysbiosis can lead to immune disregulation and susceptibility to atopy and asthma [2]. We have previously shown that bacterial alterations preceed disease in humans, and demonstrated a casual association between early-life bacterial changes and disease in mice [2]. In a recent prospective infant study, we found fungal dysbiosis to be more strongly associated with subsequent asthma risk [3]. Additionally, we found an association between antibiotic use, fungal overgrowth, and asthma risk [3].

**Objectives and methods:** We aimed to determine the impact of early-life fungal colonization and antibiotic-induced fungal overgrowth on the gut bacteriome, and the role it plays in early-life immune development. We compared cellular and humoral immune parameters in (i) conventional C57BL/6J mice treated with antibiotics and *Candida albicans*, and (ii) germ-free mice colonized with a consortium of (a) ten mouse-derived bacteria [4], (b) six yeast species colonizers of the human and mouse gut, or (c) both. Mice were systemically stimulated with ovalbumin, and we determined chemokine, cytokine, and immunoglobulins in serum, spleen, jejunum, and colon, as well as immune splenic cell types via FACS. Microbial community analysis was performed via 16S rRNA sequencing in fecal samples of mice at different stages of the experiment.

**Results:** Mice colonized with yeasts displayed broad changes across the Th1, Th2, and Th17 arms of the mucosal and systemic immune systems. Antibiotic-treated mice, with or without *C. albicans*, exhibited altered proportion of immune cell subtypes, including reduced proportion of dendritic and IL-10+ cells. The most striking changes attributed to exclusive fungal colonization were an increase in IL6+ dendritic cells, a rise in Th2 cytokines, and elevated serum IgE levels, all of which are related to atopy. Fungal colonization significantly altered the bacterial community structure and trajectory through the experiment. The presence of yeasts changed the proportion of two major bacterial colonizers (*Akkermansia municiphila* and *Bacteroides caecimuris*), known to produce important fermentation byproducts with immunological functions [5, 6]. Ongoing experiments will reveal if these changes modulate allergic inflammation, as well as the ecological consequences of early-life introduction of fungi to a bacterial community in the gut.

**Conclusion:** Our data strongly suggest that fungal-derived signal elicits immune changes related to atopy. This study also suggests that previous gnotobiotic research using only bacteria provide an incomplete portrayal of normal immune development in mice.

**References**

1. Laforest-Lapointe L, Arrieta MC. Patterns of early-life gut microbial colonization during human immune development: an ecological perspective. Front Immunol. 2017;8:788.
2. Arrieta MC et al. Early infancy microbial and metabolic alterations affect risk of childhood asthma. Sci Transl Med. 2015;7(307):307ra152
3. Arrieta MC et al. Associations between infant fungal and bacterial dysbiosis and childhood atopic wheeze in a nonindustrialized setting. J Allergy Clin Immunol. 2018;142(2):424–34.e10.
4. Brugiroux S et al. Genome-guided design of a defined mouse microbiota that confers colonization resistance against *Salmonella enterica* serovar Typhimurium. Nat Microbiol;2016;2:16215.
5. Walker WA, Iyengar RS. Breast milk, microbiota, and intestinal immune homeostasis. Pediatr Res. 2015;77(1–2):220–8
6. de Besten G, et al. The role of short-chain fatty acids in the interplay between diet, gut microbiota, and host energy metabolism. J Lipid Res. 2013; 54(9):2325–40

### A45 Impact of maternal prenatal depression on infant gut microbiota and effect modification by pet ownership

#### Khanh N. Vu^1^, Liane J. Kang^2^, Tim F. Oberlander^3^, Theodore Konya^4^, David S. Guttman^5^, Allan B. Becker^6^, Piushkumar J. Mandhane^2^, Theo J. Moraes^7^, Malcolm R. Sears^8^, Stuart E. Turvey^3^, Padmaja Subbarao^7^, James A. Scott^4^, Anita L. Kozyrskyj^1,2,9^

##### ^1^School of Public Health, University of Alberta, Edmonton, Alberta, Canada; ^2^Department of Pediatrics, University of Alberta, Edmonton, Alberta, Canada; ^3^Department of Pediatrics, University of British Columbia, Vancouver, British Columbia, Canada; ^4^Dalla Lana School of Public Health, University of Toronto, Toronto, Ontario, Canada; ^5^Centre for the Analysis of Genome Evolution and Function, University of Toronto, Toronto, Ontario, Canada; ^6^Department of Pediatrics and Child Health, University of Manitoba, Winnipeg, Manitoba, Canada; ^7^Department of Pediatrics, University of Toronto, Toronto, Ontario, Canada; ^8^Department of Medicine, McMaster University, Hamilton, Ontario, Canada; ^9^Department of Obstetrics & Gynecology, University of Alberta, Edmonton, Alberta, Canada

###### **Correspondence:** Anita L. Kozyrskyj (kozyrsky@ualberta.ca)

*Allergy, Asthma & Clinical Immunology* 2019, **15**(**Suppl 2**):A45

**Background:** Maternal psychological distress during pregnancy is linked to allergic disease development in offspring. Experimental models of stress induce gut microbial dybisosis in young rodents. Clostridial species, which are elevated in human infants following prenatal exposure to pets, increase serotonin production in colonic cells when introduced to germ-free mice. Little is known about the impact of stress on human infant gut microbiota. This study investigated the impact of maternal depressive symptoms (DS) and serotonin reuptake inhibitor (SRI) antidepressant treatment during pregnancy on microbial taxon profiles of 4-month infant stool.

**Methods:** This was a substudy of 1681 term infants from the Canadian Healthy Infant Longitudinal Development (CHILD) birth cohort. From maternal report of prenatal medication use and DS (Center of Epidemiologic Studies Depression Scale), four groups were identified: no DS or SRI use, SRI treatment with low DS levels, DS only, and both DS and SRI use. 16S rRNA sequencing determined microbial taxon profiles in infant stool. The relative abundance of infant gut microbiota at 3–4 months of age was compared across the groups of DS and SRI by multivariable negative binomial model adjusted for breast feeding, infant age and maternal intrapartum/infant antibiotic use. An interaction between maternal DS and prenatal pet exposure was tested.

**Results:** During pregnancy, one quarter of mothers experienced clinically-relevant DS and 4% took SRI antidepressants. Compared to infants of mothers with few DS, infants of mothers with prenatal DS + SRI or with prenatal DS − SRI use had higher median abundance of *Lachnospiraceae* in their gut (2.13 vs 4.62 or 3.38%, respectively). The impact of prenatal DS and/or SRI use on *Lachnospiraceae* abundance was modified by prenatal pet ownership (p_interaction_ = 0.02). In the presence of pets, *Lachnospiraceae* were enriched by 28% and 30% if the mothers had prenatal DS with or without SRI treatment, respectively; and by 47% with SRI treatment but few DS. In the absence of pets, infants of mothers having DS and receiving SRI treatment have 68% lower *Lachnospiraceae* abundance than those of mothers without DS. In the absence of pets and SRI use, there was no difference in *Lachnospiraceae* abundance between fetal/infant exposure to maternal DS versus not.

**Conclusions:** Maternal prenatal DS and/or SRI exposure may increase the abundance of *Lachnospiraceae* (from the *Clostridia* class) in 4-month-old infants but only in the presence of pets.

### A46 Differential increase in cow’s milk protein-specific IgG4/IgE ratio during cow’s milk oral immunotherapy

#### Wei Zhao^1^, Bahar Torabi^1^, Danbing Ke^2^, Duncan Lejtenyi^2^, Casey G. Cohen^1^, Moshe Ben-Shoshan^2^, Bruce D Mazer^1,2^

##### ^1^Division of Experimental Medicine, McGill University, Montreal, Quebec, H3A 0G4, Canada; ^2^Department of Pediatrics, McGill University Health Centre, Montreal, Quebec, H4A 3J1, Canada

###### **Correspondence:** Wei Zhao (wei.zhao4@mail.mcgill.ca)

*Allergy, Asthma & Clinical Immunology* 2019, **15**(**Suppl 2**):A46

**Background:** Cow’s milk allergy (CMA) is defined as an IgE-mediated reaction to cow’s milk protein (CMP) that causes an inflammatory immune response leading to clinical symptoms. There is currently no cure for CMA and the standard treatment is strict avoidance. Therefore, effective therapies are required to alleviate this burden.

Our laboratory is currently leading the first Canadian randomized-controlled trial of milk oral immunotherapy (OIT). The main goal of the trial is to enable children with CMA to tolerate ingestion of cow’s milk without developing allergy symptoms. Our laboratory has shown that CMP-specific immunoglobulin (Ig)E decreases while CMP-specific IgG4 increases at the end of the escalation phase of milk OIT. However, whether the increasing trend for IgG4/IgE ratio is consistent for all CMP components has not been determined.

**Methods:** Sixteen children with confirmed CMA by history, skin prick test, and oral food challenge were studied during a controlled trial of OIT to CMA at the Montreal Children’s Hospital. Serum was collected at specific time points during the escalation phase which ended at 200 ml of cow’s milk. We used enzyme-linked immunosorbent assays to measure the serum IgE and IgG4 specific for CMPs: α-lactalbumin (ALA), β-lactoglobulin(BLG), and casein.

**Results:** In 16 children that underwent milk OIT, the median age was 13 (± 3.61), the average duration of escalation phase was 35 weeks (± 17.8). Serum IgG4/IgE ratio between baseline and the 200 ml dose increased significantly for BLG (p < 0.0001) and casein (p = 0.0067). All the children had increased IgG4/IgE ratio for BLG, while 14 out of 16 children had increased IgG4/IgE ratio for casein. Only 11 out of 16 children had increased IgG4/IgE ratio for ALA.

**Conclusions:** We determined that while IgG4/IgE ratio increase in participants that can consume 200 ml of cow’s milk, this trend is not consistent among the CMP components. This may lead to unforeseen reactions.

**Acknowledgements:** This work was supported by Canadian Institute of Health Research and AllerGen NCE (the Allergy, Genes, and Environment Network). We would like to thank the participants and health professionals involved in the milk OIT trial from the McGill University Health Centre, British Columbia Children Hospital, Centre Hospitalier Universitaire Sainte-Justine, and The Hospital for Sick Children.

## Late-Breaking Category

### LB1 Early life exposure to respiratory viruses and risk of asthma at school age and adolescence

#### Salma Bahreinian^1^, Richard G. Hegele^2, 3^, Wade T.A. Watson^4^, Clare D Ramsey^5, 6^, Edmond S. Chan^7^, Allan B. Becker^1,8^, Elinor Simons^1,6,8^

##### ^1^Section of Allergy and Clinical Immunology, Department of Pediatrics and Child Health, University of Manitoba, Winnipeg, Manitoba, Canada; ^2^Department of Laboratory Medicine and Pathobiology, University of Toronto, Toronto, Ontario, Canada; ^3^Department of Paediatric Laboratory Medicine, The Hospital for Sick Children, Toronto, Ontario, Canada; ^4^Department of Pediatrics, Division of Allergy, IWK Health Centre, Dalhousie University, Halifax, Nova Scotia, Canada; ^5^Department of Internal Medicine, University of Manitoba, Winnipeg, Manitoba, Canada; ^6^Department of Community Health Sciences, University of Manitoba, Winnipeg, Manitoba, Canada; ^7^Division of Allergy and Immunology, Department of Pediatrics, British Columbia Children’s Hospital, University of British Columbia, Vancouver, BC, Canada; ^8^Children’s Hospital Research Institute of Manitoba, Winnipeg, Manitoba, Canada

###### **Correspondence:** Salma Bahreinian (bahreins@myumanitoba.ca)

*Allergy, Asthma & Clinical Immunology* 2019, **15**(**Suppl 2**):LB1

**Background:** Early life exposure to common respiratory viruses has been linked with development of wheezing in preschoolers. The relation between respiratory viruses and persistent asthma in later childhood and adolescence remains controversial.

**Methods:** The Canadian Asthma Primary Prevention Study (CAPPS) recruited 549 infants at birth with asthma or allergies in first-degree relatives. Respiratory symptoms were prospectively recorded. Frozen nasal specimens were obtained at 2 weeks and 4 months of age, and were analysed via reverse-transcription polymerase chain reaction (RT-PCR) for parainfluenza virus, respiratory syncytial virus (RSV), and picornavirus (rhinovirus type A and B/enterovirus). At ages 7 and 15 years, skin prick testing to foods and inhalants was performed and asthma was diagnosed based on clinical assessments by pediatric allergists and methacholine challenges.

**Results:** Asthma was diagnosed in 71/378 (18.8%) and 51/302 (16.9%) of children at ages 7 and 15 years, respectively. A positive nasal swab for at least one virus was associated with parental report of having a cold (but not cough) at 2 weeks (p = 0.001), and both cough and cold at 4 months (p = 0.01 and p = 0.001, respectively). Cough at 2 weeks increased the odds of having asthma at 15 years, independent of the viral swab results (OR 2.87, 95% CI 1.20–6.90). Children with a positive RSV swab at age 4 months had significantly increased odds of asthma at 7 years (OR 2.67, 95% CI 1.01–7.05) but not 15 years (OR 2.35, 95% CI 0.78–7.09). After adjusting for possible confounders, the association remained significant for asthma at 7 years (Table 1). No statistically-significant associations were found with other viruses.

**Table 1:** Association of positive nasal swab for respiratory syncytial virus (RSV) at 4 months of age with asthma at 7 and 15 years

	Asthma with RSV colonization or infection at 4 months of age	
	Model 1 Adjusted OR (95% CI)	Model 2 Adjusted OR (95% CI)
Asthma at age 7 years	4.57* (1.49–14.03)	4.63* (1.48–14.45)
Asthma at age 15 years	3.73* (1.01–13.84)	3.40 (0.84–13.77)

Model 1 is adjusted for gender, parental history of asthma, city of residence (Winnipeg or Vancouver), atopic dermatitis at 1 year and positive skin test to at least one allergen at 7 years

Model 2 is adjusted for gender, parental history of asthma, city of residence (Winnipeg or Vancouver), atopic dermatitis at 1 year, positive skin test to at least one allergen at 7 years and cough at 2 weeks

*Statistically significant (p < 0.05)

**Conclusion:** The link between RSV infection at 4 months and asthma at age 7 years remains suggestive at age 15 years, but is no longer statistically significant. However, cough at age 2 weeks remains a significant indicator of asthma at age 15 years, independent of viral exposures in the first 4 months of life. These results demonstrate that RSV infection or colonization is a risk factor for transient asthma; in keeping with some previous studies [1], but not others [2]. This is the first study to demonstrate that persistent asthma at 15 years may be associated with the presence of cough (with or without a cold) as early as 2 weeks of age. This finding may contribute to early recognition of infants at higher risk of asthma.

**Acknowledgements:** We would like to thank all the members of CAPPS research team.

**References**

1. Rubner FJ, Jackson DJ, Evans MD, Gangnon RE, Tisler CJ, Pappas TE, Gern JE, Lemanske RF Jr. Early life rhinovirus wheezing, allergic sensitization, and asthma risk at adolescence. J Allergy Clin Immunol. 2017;139:501–7.
2. Ruotsalainen M, Hyvarinen MK, Piippo-Savolainen E, Korppi M. Adolescent asthma after rhinovirus and respiratory syncytial virus bronchiolitis. Pediatr Pulmonol. 2013;48:633–9.

### LB2 Phenome-wide association study of genetic variants in known asthma loci in the CHILD Study

#### Ekaterina Dunets^1,2^, Amirtha Ambalavanan^1^, Jihoon Choi^1^, Amel Lamri^3^, Diana L. Lefebvre^4^, Sonia S. Anand^4^, Guillaume Pare^5^, Stuart E. Turvey^6^, Piush J. Mandhane^7^, Allan B. Becker^8^, Meghan B. Azad^8^, Theo J. Moraes^9,10^, Malcolm R. Sears^4^, Padmaja Subbarao^9,10,11^, Qingling Duan^1,2^

##### ^1^Department of Biomedical and Molecular Sciences, Queen’s University, Kingston, Ontario, Canada; ^2^School of Computing, Queen’s University, Kingston, Ontario, Canada; ^3^Department of Clinical Epidemiology and Biostatistics, McMaster University, Hamilton, Ontario, Canada; ^4^Department of Medicine, McMaster University, Hamilton, Ontario, Canada; ^5^Pathology and Molecular Medicine, McMaster University, Hamilton, Ontario, Canada; ^6^Division of Allergy and Immunology, Department of Pediatrics, University of British Columbia, British Columbia, Canada; ^7^Division of Pediatric Respiratory Medicine, University of Alberta, Alberta, Canada; ^8^Department of Pediatrics and Child Health, University of Manitoba, Manitoba, Canada; ^9^Department of Paediatrics, University of Toronto, Toronto, Ontario, Canada; ^10^The Hospital for Sick Children, Toronto, Ontario, Canada; ^11^Division of Respirology, Department of Medicine, McMaster University, Hamilton, Ontario, Canada

###### **Correspondence:** Qingling Duan (qingling.duan@queensu.ca)

*Allergy, Asthma & Clinical Immunology* 2019, **15**(**Suppl 2**):LB2

**Background:** Asthma is a chronic respiratory disease that affects 13% of Canadian children [1]. Genome-wide association studies (GWAS) of asthma have identified numerous genetic associations [1]. While these earlier studies support the role of genetic variability underlying asthma risk, many of these asthma-associated variants have also been identified in studies of other phenotypes such as wheeze and atopy. These associations with multiple phenotypes indicate the possibility for common disease mechanisms or pleiotropic effects of these variants on multiple health outcomes. The aim of this study was to investigate the association of genetic variants in well-known asthma loci with other phenotypes (both related and unrelated) in a single population using an approach known as a phenome-wide association study (PheWAS).

**Methods:** Our study involved 2830 children from the Canadian Healthy Infant Longitudinal Development (CHILD) birth cohort. Primary phenotypic outcomes include atopy, asthma diagnosis, and wheezing episodes (total of 68 phenotypic variables by age 5). Genotyping data was obtained using the Illumina HumanCoreExome BeadChip and imputations were derived using *IMPUTE2* based on sequence data from the 1000 Genomes Project. A comprehensive list of 447 asthma-associated variants from published literature, and their associated linkage disequilibrium variants (LD), were previously compiled in our laboratory and applied in this study [2]. We performed a PheWAS of these single nucleotide polymorphisms (SNPs) in known asthma loci for all 68 phenotypes (linear regression model).

**Results:** We identified several SNPs (in known asthma genes such as *ORMDL3* and *GSTP1*) associated with asthma as well as atopy and recurrent wheeze in the CHILD Study. For example, a well-established asthma SNP, rs8067378, was found to show high association with recurrent wheeze between ages 2–5 years (*P *= 5.35 × 10^−8^) as well as with positive asthma diagnosis at age 5 years (*P *= 6.81 × 10^−4^). These associations suggest a shared genetic predisposition or disease mechanism underlying these related phenotypes.

**Conclusions:** Our study demonstrates that genetic variants previously associated with asthma are also strongly correlated with other related phenotypes in the CHILD Study. We report several novel associations between known asthma variants and recurrent wheeze, as well as atopy in the CHILD Study. Ongoing work in our lab includes the expansion of our PheWAS study to include larger datasets such as the UK Biobank and eMERGE, which consist of tens to hundreds of phenotypes, many of which are unrelated to asthma in order to further investigate the potential for pleiotropic effects of asthma variants.

**Acknowledgements:** This work is supported through funding from the Canadian Institutes of Health Research and the AllerGen NCE Inc. Computations were performed with support provided by the Centre for Advanced Computing (CAC) at Queen’s University. The CAC is funded by: The Canada Foundation for Innovation, the Government of Ontario, and Queen’s University.

**References**

1. Vicente C, Revez J, Ferreira M. Lessons from ten years of genome-wide association studies of asthma. Clin Transl Immunol. 2017;6(12):e165.
2. Neville M, Choi J, Lieberman J, Duan Q. Identification of deleterious and regulatory genomic variations in known asthma loci. Respir Res. 2018;19.

### LB3 Stability of serum precipitins to *Aspergillus fumigatus* for the diagnosis of allergic bronchopulmonary aspergillosis

#### Kevin S.K. Lau^1,2,3^, Chantane Yeung^1,2,3^, Shu Yu Fan^1,2,3^, Chris Carlsten^1,2,3^

##### ^1^Division of Respiratory Medicine, Department of Medicine, University of British Columbia, Vancouver, BC, Canada; ^2^Centre for Heart Lung Innovation, University of British Columbia, Vancouver, BC, Canada; ^3^Chan-Yeung Center for Occupational and Environmental Respiratory Disease, University of British Columbia, Vancouver, BC, Canada

###### **Correspondence:** Kevin S.K. Lau (kevin.lau@ubc.ca)

*Allergy, Asthma & Clinical Immunology* 2019, **15**(**Suppl 2**):LB3

**Background:** Allergic bronchopulmonary aspergillosis (ABPA) is a hypersensitivity and exaggerated immune system response to colonization of the airways by *Aspergillus fumigatus* [1–3]. ABPA occurs in individuals with asthma or cystic fibrosis and is associated with worse outcomes for individuals with these conditions [1–3]. Each year, the province of BC receives over 2600 diagnostic testing requests at a centralized location in Vancouver, requiring specimen collection, storage, and shipment from different clinics across the province. Timely, reliable, and cost-effective testing of *Aspergillus* precipitins is critical in the diagnosis and management of ABPA. At our centre, we analyzed sample stability in varying storage conditions to provide provincial guidelines to improve this routine cost-effective diagnostic testing.

**Methods:** To determine temperature and time stability, 31 serum specimens positive for *Aspergillus fumigatus* precipitins from routine clinical testing were each aliquoted into two conditions and incubated at 4 °C (control) and 37 °C. Samples were repeatedly assayed for precipitins to *Aspergillus fumigatus* via agarose gel double immunodiffusion (AGID) at seven, 14, and 28 days post-incubation. To determine freeze–thaw stability, 39 serum specimens positive and 39 specimens negative for *Aspergillus fumigatus* precipitins from routine clinical testing were randomly selected and the diagnostic results were blinded to the operators. Each specimen was aliquoted into four conditions and incubated at 4 °C (control; one group) or − 20 °C (three groups). − 20 °C samples were thawed at room temperature and immediately refrozen overnight; 4 °C samples were maintained at 4 °C. Samples were assayed for precipitins to *Aspergillus fumigatus* via AGID following none (4 °C), one, two, or three freeze–thaw cycles.

**Results:** Regarding temperature and time stability, median stability time was 47 and 34 days at 4 °C and 37 °C, respectively. Cox proportional-hazards model indicates no statistically significant difference between the two temperature storage conditions (p = 0.188) with a hazard ratio of 1.576 (95% CI 0.8012 to 3.099). Regarding freeze–thaw stability, no indication of serum degradation with regards to *Aspergillus fumigatus* precipitins was found with repeated freeze–thaw cycles as compared to refrigerated storage.

**Conclusions:** The stability of serum precipitins to *Aspergillus fumigatus* was found to be dependent on time, but not temperature and freeze–thaw cycles. As a cost-effective measure, specimens for *Aspergillus fumigatus* precipitins testing should be shipped at ambient temperature and tested within 2 weeks from collection.

**Acknowledgements:** The authors acknowledge Min Hyung Ryu for editorial input.

**References**

1. Greenberger PA. Allergic bronchopulmonary aspergillosis. J Allergy Clin Immunol. 2002;110:685–92.
2. Zander DS. Allergic bronchopulmonary aspergillosis: an overview. Arch Pathol Lab Med. 2005;129:924–8.
3. Agarwal R. Allergic bronchopulmonary aspergillosis. Chest. 2009;135:805–26.

### LB4 Omics Central: a web application for analyzing omics datasets, sharing analyses and building reproducible workflows

#### Amrit Singh^1,2^, Scott J. Tebbutt^2,3,4^

##### ^1^Department of Pathology and Laboratory Medicine, University of British Columbia, Vancouver, Canada; ^2^PROOF Centre of Excellence, Vancouver, British Columbia, Canada; ^3^UBC Centre for Heart and Lung Innovation, Vancouver, Canada; ^4^Department of Medicine, Division of Respiratory Medicine, UBC, Vancouver, Canada

###### **Correspondence:** Amrit Singh (Amrit.Singh@hli.ubc.ca)

*Allergy, Asthma & Clinical Immunology* 2019, **15**(**Suppl 2**):LB4

**Background:** Omics technologies are being widely used by researchers to identify biomarkers of disease. Although the technologies for generating and preprocessing different data types (e.g. genomics, transcriptomics, proteomics) differ, the downstream analytical methods are often the same. We are implementing a web application that provides users with a pipeline of methods including machine-learning algorithms, gene set enrichment analysis, and resources for literature mining. Each aspect of the pipeline is accompanied with various interactive visualizations to maximize interpretability of the results.

**Methods:** This web application is based on a MongoDB, Express.js, React.js and Node.js (MERN) stack, a custom application program interface (API) for data analytics using R (deployed using Docker) and leverages various external APIs such as Enrichr (for gene set enrichment analysis), InnateDB (for protein–protein interactions) and Entrez Utilities (for literature searches). User authentication is performed using Google OAuth 2.0. A data analytics dashboard provides interactive visualizations to aid in the interpretability of various analyses (*e.g.* exploratory data analysis, classification algorithms). The website will be deployed using the cloud application platform Heroku. We used an unpublished asthma dataset (of blood expression profiles of early and dual asthmatic responders) to identify biomarkers of the late-phase asthmatic response and attribute them to potential biological mechanisms.

**Results:** 770 genes related to immune responses were measured using the PanCancer Immune Profiling Panel. Six hundred (89 cell-specific genes and 511 immune response genes) out of the 770 genes were retained after filtering of low abundant transcripts and housekeeping genes. The optimal biomarker panel based on sparse partial least squares discriminant analysis consisted of two components with 115 genes selected per component (a total of 208 genes) with an error rate of 31.9% (AUC = 0.79) based on a nested leave-one-out cross-validation. Top ranked pathways (FDR < 5%), included *CD molecules*, *Innate immune response*, *Adaptive immune response*, *Regulation of immune response*, *Chemokines and receptors* and *Cytokines and receptors.*

**Conclusions:** Omics Central is a graphical user interface that enables researchers to apply a pipeline of data analysis methods in order to identify biomarkers and implicate potential biological mechanisms in their condition of interest. In this study, we use Omics Central to identify a biomarker panel of the late-phase asthmatic response and attribute various biological roles to these biomarkers. Future works include network analysis to incorporate curated relationships between biomarkers, and literature mining to generate additional hypotheses for downstream experiments.

### LB5 Main and interaction effects of genetics and environmental exposures on risk of childhood atopy in the CHILD Study

#### Emma Vanlerberghe^1,2^, Jihoon Choi^1^, Amirtha Ambalavanan^1^, Amel Lamri^3^, Diana L. Lefebvre^4^, Sonia S. Anand^4^, Guillaume Pare^5^, Stuart E. Turvey^6^, Piush J. Mandhane^7^, Allan B. Becker^8^, Meghan B. Azad^8^, Theo J. Moraes^9,10^, Malcolm R. Sears^4^, Padmaja Subbarao^9,10,11^, Qingling Duan^1,2^

##### ^1^Department of Biomedical and Molecular Sciences, Queen’s University, Kingston, Ontario, Canada; ^2^School of Computing, Queen’s University, Kingston, Ontario, Canada; ^3^Department of Clinical Epidemiology and Biostatistics, McMaster University, Hamilton, Ontario, Canada; ^4^Department of Medicine, McMaster University, Hamilton, Ontario, Canada; ^5^Pathology and Molecular Medicine, McMaster University, Hamilton, Ontario, Canada; ^6^Division of Allergy and Immunology, Department of Pediatrics, University of British Columbia, British Columbia, Canada; ^7^Division of Pediatric Respiratory Medicine, University of Alberta, Alberta, Canada; ^8^Department of Pediatrics and Child Health, University of Manitoba, Manitoba, Canada; ^9^Department of Paediatrics, University of Toronto, Toronto, Ontario, Canada; ^10^The Hospital for Sick Children, Toronto, Ontario, Canada; ^11^Division of Respirology, Department of Medicine, McMaster University, Hamilton, Ontario, Canada

###### **Correspondence:** Qingling Duan (qingling.duan@queensu.ca)

*Allergy, Asthma & Clinical Immunology* 2019, **15**(**Suppl 2**):LB5

**Background:** The prevalence of allergies has increased in Canada and globally in recent decades with approximately 7.5% of Canadians self-reported at least one food allergy and over 20% having seasonal allergies [1]. Common symptoms range from mild to severe such as wheezing, vomiting, hives and anaphylactic reactions (from ingested allergens). In this study, we test the hypothesis that both genetic and environmental factors contribute to risk of developing allergies by determining both the main and interaction effects of genes and environmental exposures. Specifically, we conducted genome-wide association studies (GWAS) of atopic outcomes among infants in the Canadian Healthy Infant Longitudinal Development (CHILD) Study, as well as examined the interaction effects of environmental exposures on the prevalence of these outcomes.

**Methods:** Genotype data was obtained for 2830 children using the Illumina HumanCoreExome BeadChip, which included approximately 500,000 single nucleotide variants (50% rare with minor allele frequencies (MAF) < 0.01). These were then used to impute genotypes of nearly 23 million variants based on sequence data from the 1000 Genomes Project using *IMPUTE2* [2, 3]. The primary outcomes of our analysis included positive skin prick tests to food allergens (e.g., milk, eggs and peanuts) and inhalants (e.g., fungus and cat hair) as binary variables. We applied logistic regression analysis using genotypes as an additive model (0, 1, 2) for approximately 6 million common variants (MAF ≥ 0.01) in *PLINK* [4, 5]. Environmental exposures such as breastfeeding duration, pre- and post-natal cigarette exposures and pet ownership were used as interaction terms in our genetic association analysis.

**Results:** Our GWAS of childhood atopic outcomes in Caucasian subjects identified multiple SNPs significantly associated with food allergies and inhalants at age 1, 3 and 5 years. For example, multiple associated SNPs were located in novel genetic regions on chromosomes 6p22.3 and 10p23.1, which are proximal to genes *MYLIP* (encodes Myosin Regulatory Light Chain Interacting Protein) and *NRG3* (encodes Neuregulin 3), respectively. Gene–environment interaction analyses are underway to determine the modifying effects of exposures such as breastfeeding (duration and milk composition from mothers), dog ownership and cigarette smoke on risk of atopy.

**Conclusion:** Our GWAS analysis of atopic outcomes in the CHILD Study identified novel genetics loci associated with these health outcomes. Our ongoing analyses includes all subjects across multiple ethnicities in the CHILD Study, gene-environment interaction analyses as well as rare variants using gene-based analysis.

**Acknowledgements:** This work is supported by funding from AllerGen NCE Inc. and Canadian Institutes of Health Research. Computations were performed with support provided by the Centre for Advanced Computing (CAC) at Queen’s University. The CAC is funded by: The Canada Foundation for Innovation, the Government of Ontario, and Queen’s University.

**References**

1. Soller L, et al. Overall prevalence of self-reported food allergy in Canada. J Allergy Clin Immunol. 2012;130(4):986–8.
2. Howie B, Donnelly P, Marchini J. A flexible and accurate genotype imputation method for the next generation of genome-wide association studies. PLoS Genet. 2009;5(6):e1000529.
3. Howie B, Marchini J, Stephens M. Genotype imputation with thousands of genomes. G3 Genes Genom Genet. 2011;1(6):457–70.
4. Purcell S, Neale B, Todd-Brown K, Thomas L, Ferreira MAR, Bender D, Maller J, Daly MJ, Sham PC. PLINK: a toolset for whole-genome association and population-based linkage analysis. Am J Hum Genet. 2007;81.
5. PLINK1.9, Shaun Purcell, http://pngu.mgh.harvard.edu/purcell/plink/.

### LB6 Pilot study of using deciduous teeth to reflect metal exposure in neonatal periods

#### Yuanyuan Xie^1^, Vanessa R.N. Cruvinel^2^, Greg J. Evans^1,3^, Jeffrey R. Brook^1,3^

##### ^1^Department of Chemical Engineering and Applied Chemistry, University of Toronto, Toronto, ON, Canada; ^2^Department of Public Health of the Faculty of Ceilandia, University of Brasilia, Brasilia, Brazil; ^3^Dalla Lana School of Public Health, University of Toronto, Toronto, ON, Canada

###### **Correspondence:** Greg J. Evans (greg.evans@utoronto.ca), Jeffrey R. Brook (jeff.brook@utoronto.ca)

*Allergy, Asthma & Clinical Immunology* 2019, **15**(**Suppl 2**):LB6

**Background:** Exposure to high levels of toxic metals could lead to adverse health outcomes in the human body [1, 2]. This issue could be more severe when considering the harmful impacts to neonates and children, a susceptible population whose organs are still developing, which means they have a less effective blood–brain barrier and underdeveloped mechanisms to excrete these toxic chemicals [3]. Therefore, it is useful to reveal chemical exposure in the neonatal periods. Metals incorporate into the hydroxyapatite matrix of teeth during the formation processes and cannot be remolded easily afterwards. As teeth have been widely used to indicate long-term metal exposure, and metal levels in teeth have been linked to neurobehavioral outcomes and metal pollution levels [4–6], the CHILD Study in Canada is currently obtaining teeth for future research involving exposure assessment. In addition, with the clearer understanding of the layer-to-layer formation mechanism and the identification of the neonatal line in deciduous teeth, metal exposure can be estimated with a time range from the second trimester to about 1 year after birth.

**Methods:** In this pilot study, the use of deciduous teeth to reflect metal exposure has been explored. The total metal concentrations (Pb, Mn, Cu, Zn, As, Cd, and Ba) in deciduous teeth collected from our co-author in Brazil were determined with an Inductively Coupled Plasma Mass Spectrometry (ICP-MS) after microwave digestion. Furthermore, micro-spatial analysis of metal levels was performed with a Laser Ablation Inductively Coupled Mass Spectrometry (LA-ICP-MS).

**Results:** The results showed that total metal levels in the deciduous teeth ranged from ppb to ppm levels (dry weight). In addition, micro-sampling of metals in enamel and dentine along the enamel-dentine junction revealed significant variations in metal levels. In the first incisor analyzed, the ^208^Pb/^44^Ca values ranged from 3.85 × 10^−6^ to 8.61 × 10^−6^ in enamel and 1.49 × 10^−5^ to 3.89 × 10^−5^ in dentine. For Cr and Cd, most of their values were below the detection limit in enamel. In dentine, ^53^Cr/^44^Ca ranged from 1.16 × 10^−6^ to 3.42 × 10^−6^, and ^111^Cd/^44^Ca ranged from 1.02 × 10^−7^ to 1.02 × 10^−6^.

**Conclusions:** The variations of metal concentrations in different layers of deciduous teeth indicated different metal exposure levels during the formation processes. The combination of total metal concentration and micro-spatial analysis can provide valuable information on both cumulative amount and timing of metal exposure.

**Acknowledgements:** This work was supported by AllerGen NCE Inc. The author would thank Nancy Valiquette in the Department of Dentistry for helping prepare tooth slices and Prof. Zoltan Zajacz in the Department of Geology for the guidance about using LA-ICP-MS. And the author would thank all members of SOCAAR (Sothern Ontario Center for Atmospheric Aerosol Research) for their support.

**References**

1. Valko M, Morris H, Cronin M. Metals, toxicity and oxidative stress. Curr Med Chem. 2005;12(10):1161–208.
2. Bagchi D, Joshi SS, Bagchi M, Balmoori J, Benner EJ, Kuszynski CA, Stohs SJ. Cadmium- and chromium-induced oxidative stress, DNA damage, and apoptotic cell death in cultured human chronic myelogenous leukemic K562 cells, promyelocytic leukemic HL-60 cells, and normal human peripheral blood mononuclear cells. J Biochem Mol Toxicol. 2000;14(1):33–41.
3. Oskarsson A, Palminger Hallen I, Sundberg J, Petersson Grawe K. Risk assessment in relation to neonatal metal exposure. Analyst. 1998;123(1):19–23.
4. Mora AM, Arora M, Harley KG, Kogut K, Parra K, Hernandez-Bonilla D, Gunier RB, Bradman A, Smith DR, Eskenazi B. Prenatal and postnatal manganese teeth levels and neurodevelopment at 7, 9, and 10.5 years in the CHAMACOS cohort. Environ Int. 2015;84:39–54.
5. Arora M, Reichenberg A, Willfors C, Austin C, Gennings C, Berggren S, Lichtenstein P, Anckarsater H, Tammimies K, Bolte S. Fetal and postnatal metal dysregulation in autism. Nat Commun. 2017;8:15493.
6. Appleton J, Lee KM, Sawicka Kapusta K, Damek M, Cooke M. The heavy metal content of the teeth of the bank vole (Clethrionomys glareolus) as an exposure marker of environmental pollution in Poland. Environ Pollut. 2000;110(3):441–9

